# Advances in omics technologies for traditional Chinese medicine in the prevention and treatment of metabolic bone diseases

**DOI:** 10.3389/fphar.2025.1576286

**Published:** 2025-04-11

**Authors:** Wenjun Cai, Lili Jiang, Changwei Zhao, Xiaoling Zhou

**Affiliations:** ^1^ Changchun University of Chinese Medicine, Changchun, China; ^2^ Department of Orthopedics, The Third Affiliated Clinical Hospital of Changchun University of Chinese Medicine, Changchun, China; ^3^ Department of Endocrinology, Changchun Hospital of Chinese Medicine, Changchun, China; ^4^ Department of Orthopedics, The Affiliated Hospital of Changchun University of Chinese Medicine. Changchun, China; ^5^ Department of Geriatrics, The Affiliated Hospital of Changchun University of Chinese Medicine. Changchun, China

**Keywords:** metabolic bone disease, traditional Chinese medicine (TCM), metabolomics, proteomics, transcriptomics

## Abstract

Metabolic bone disease (MBD), as one of the most severe metabolic disorders, remains a focal point and challenge in medical research. Numerous studies have demonstrated the efficacy of Traditional Chinese Medicine (TCM) in preventing and treating MBD. However, the inherent complexity of TCM metabolites poses significant limitations in elucidating their mechanisms of action. The advancement of omics technologies, including metabolomics, proteomics, and transcriptomics, has greatly facilitated research on MBD. These approaches enable the identification of potential biomarkers and the exploration of metabolic pathways and mechanisms underlying TCM interventions for MBD. Evidence indicates that TCM monomers, single botanical drugs, and herbal formulations are effective, safe, and well-tolerated in MBD prevention and treatment. This review summarizes recent applications and key findings of transcriptomics, proteomics, and metabolomics in studying the mechanisms of TCM interventions for MBD. It highlights the role of omics technologies in uncovering relevant metabolites and pathways under TCM treatment, providing valuable insights and clinical references for TCM-based strategies in managing MBD.

## 1 Introduction

Metabolic Bone Disease (MBD) encompasses a group of disorders characterized by abnormalities in bone metabolism, structure, or mineralization, and is clinically significant due to its impact on skeletal health. Clinically, MBD is primarily manifested by abnormal bone turnover rates, accompanied by symptoms such as bone pain, deformities, and fractures. It belongs to a distinct category within metabolic diseases (Seemann et al., 2024). Bone tissue undergoes continuous remodeling, a dynamic process involving daily bone formation and resorption. An adult’s skeleton is entirely renewed approximately every 10 years (Hart et al., 2020). The stability of bone metabolism and bone mass depends on the dynamic balance between osteoblast-mediated bone formation and osteoclast-mediated bone resorption, which is maintained by a normal bone marrow microenvironment. Disruption of this balance leads to MBD, increasing the risk of skeletal abnormalities and fractures. MBD include Osteoporosis (OP), Osteomalacia, Mucopolysaccharidosis, Marfan Syndrome, Paget’s Disease, and Renal Osteodystrophy, and so on (Feng and McDonald, 2011). Among these, OP is the most prevalent form of MBD (Anonymous, 1993). Globally, approximately 18.3% of the population suffers from OP, with a significantly higher incidence in women (23.1%) than in men (11.7%). Current treatment strategies focus on supplementing vitamin D, calcium, estrogen replacement, parathyroid hormone analogs, or bisphosphonates. These interventions enhance bone response, inhibit osteoclast activity, and slow down bone metabolism (Johnston and Dagar, 2020).

In traditional Chinese medicine (TCM), MBD is named as “Guwei,” “Guku,” and “Gubi” based on their clinical manifestations. Its pathogenesis is closely linked to the kidney, bone, and marrow. Classical theories posit that “marrow is generated from kidney essence; sufficient essence leads to abundant marrow, and abundant marrow results in strong bones.” This highlights the intrinsic connection between sufficient kidney essence, abundant marrow, and robust bones. Therefore, the occurrence of bone diseases is closely associated with the functional state of the kidney. The pathogenesis of MBD may involve multiple factors, including insufficient innate endowment, age-related decline in kidney essence, liver stagnation, spleen deficiency, impaired generation of qi and blood, inadequate nourishment of kidney essence and bone marrow, and poor blood circulation. Clinically, TCM employs botanical drugs that nourish the liver and kidney, strengthen the spleen and kidney, and invigorate the tendons and bones. These botanical drugs also promote blood circulation and remove blood stasis, strengthen the kidneys and bones, thereby alleviating clinical symptoms such as weakness, fatigue, and soreness in the lower back and knees.

In recent years, active metabolites of TCM, such as icariin and achyranthes bidentata polysaccharides, have been scientifically validated for their ability to enhance bone mineral density (BMD) and improve bone microstructure (Xue et al., 2016; Zhang et al., 2018). Its mechanisms of action primarily involve regulating metabolite levels, protein expression ratios, restoring metabolite diversity, and modulating gene expression levels, effectively delaying the onset and progression of MBD. Studies have shown that both single botanical drugs and Chinese herbal formula can improve bone turnover processes, optimize bone tissue structure, and reverse declining bone density by regulating serum biochemical indicators. These findings demonstrate the significant role of TCM in regulating bone metabolism, offering new therapeutic strategies for MBD prevention and treatment.

In recent years, as an emerging interdisciplinary field, omics technologies, including proteomics, transcriptomics, metabolomics, and others, have attracted significant scientific interest. Omics technologies enable the systematic profiling of genetic information, transcriptional data, proteins, and metabolites from an entire organism, tissue, organ, or single cell, providing in-depth analysis of the composition and interrelationships of these metabolites. This is highly consistent with the holistic concept of TCM. Metabolomics focuses on the quantitative profiling of all small-molecule metabolites within an organism using techniques such as gas chromatography-mass spectrometry, liquid chromatography-mass spectrometry (LC-MS), and nuclear magnetic resonance. It establishes various mathematical models to identify differential metabolites through statistical methods and conducts bioinformatics analysis to reveal the pathophysiological implications of metabolic changes in the overall system caused by diseases or interventions (Huang R. et al., 2023). Proteomics, based on protein separation techniques, mass spectrometry identification, and bioinformatics, investigates protein functions, structures, and interactions. Commonly used methods include two-dimensional gel electrophoresis, mass spectrometry, and isobaric tags for relative and absolute quantification (Deng et al., 2022). Transcriptomics employs high-throughput RNA sequencing, single-cell sequencing, and microarray technologies to comprehensively characterize all RNA within cells during specific periods, thereby uncovering gene expression patterns and regulatory mechanisms (Chambers et al., 2019). The samples involved in these studies include blood, urine, bone, liver, and kidney tissues, among others (Tao et al., 2017; Li X. et al., 2022).

The rapid development and widespread application of omics technologies have provided new perspectives for exploring the etiology and pathogenesis of MBD, both epistemologically and methodologically. They promote the standardization of syndrome differentiation treatment and provide scientific evidence for analyzing the material basis of TCM and Chinese herbal formula. Omics technologies also provide technical support for revealing the molecular mechanisms of complex drug systems, enabling intervention in diseases at multiple levels and dimensions. With the rapid development of modern systems biology, continuous innovation in proteomics, metabolomics, and transcriptomics, and the refinement of bioinformatics methods, research on TCM for preventing and treating MBD is expected to achieve significant breakthroughs.

Through a systematic review and comprehensive analysis of the literature, this study aims to evaluate the current research status of MBD treatment based on metabolomics, proteomics, and transcriptomics. Furthermore, it seeks to elucidate the underlying mechanisms of action, assess their clinical efficacy, and provide evidence-based recommendations for future research directions.

## 2 Methods

### 2.1 Search strategy

This review searched literature from the establishment of the databases up to December 2024. The databases searched include CNKI, Wanfang, VIP, and PubMed. Key terms used in the search were “metabolomics,” “proteomics,” “transcriptomics,” “metabolic bone disease,” “osteoporosis,” “diabetic bone disease,” “renal osteodystrophy,” “Paget’s disease,” and “TCM.” Both Chinese and English literature were included.

### 2.2 Selection criteria

First, we conducted a title screening of the literature to assess its relevance to the research topic. Subsequently, based on the results of the title screening, we performed a secondary screening by reviewing the abstracts of the papers. Finally, we conducted a full-text read of the selected literature to ensure that its content was consistent with the objectives of the review.

During the screening process, we rigorously applied pre-defined inclusion and exclusion criteria. The inclusion criteria were as follows: studies must focus on omics (e.g., metabolomics, proteomics, transcriptomics), MBDs, TCM, or natural products. Additionally, studies must address the pathophysiological mechanisms of MBDs or the prevention and treatment of these diseases using TCM. Furthermore, studies must explicitly investigate the role of omics technologies in elucidating the mechanisms of TCM for preventing or treating MBDs. The exclusion criteria included: studies with unclear themes, methods, or mechanisms; publications of unreliable or substandard quality; duplicate publications or studies; studies with incomplete or insufficient data, dissertations, or studies for which the full text was inaccessible. To ensure objectivity and reliability in the screening process, two researchers (WC and LJ) independently conducted the literature screening and analysis. Discrepancies were resolved through discussion to reach a consensus, and unresolved issues were adjudicated by a third researcher (CZ). This approach significantly reduced subjective bias and guaranteed the scientific rigor and reliability of the final included literature.

## 3 Pathogenesis of MBD

**FIGURE 1:**
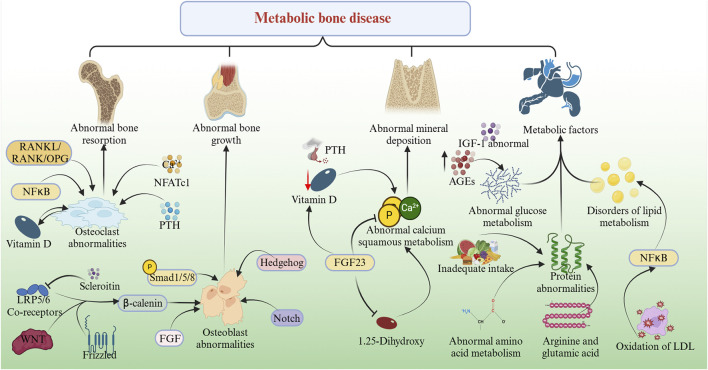
Mechanistic map of metabolic bone disease: The pathogenesis of MBD primarily involves four key pathological dimensions: abnormal bone resorption, abnormal bone formation, abnormal mineral deposition, and metabolic factors. ① the core regulatory pathways for abnormal bone resorption are the RANKL/RANK/OPG system, NF-κB and Ca^2+^-NFATc1 signaling pathway. PTH and vitamin D hormones also significantly influence bone resorption. ② Abnormal bone formation mainly involves osteoblast dysfunction. The Wnt/β-catenin and BMP/Smad signaling pathway play key roles in osteoblast differentiation and function. Other signaling pathways such as Hedgehog, Notch, and FGF also participate in the regulation of osteoblasts. ③ Abnormal mineral deposition is primarily manifested as disorders in calcium and phosphorus metabolism. Vitamin D deficiency and parathyroid dysfunction can lead to imbalances in calcium and phosphorus metabolism. Additionally, FGF23 plays a crucial role in the regulation of mineral metabolism. ④ The impact of metabolic factors mainly involves the effects of abnormalities in sugar, fat, and protein metabolism on bone metabolism.

Omics technologies hold significant value in the field of disease research, as they not only enable differential analysis of metabolites and proteins but also correlate data with biological significance. In the context of MBD, omics technologies have provided powerful tools for elucidating their pathogenesis. Current research indicates that the pathogenesis of MBD primarily involves four key aspects: abnormal bone resorption, abnormal bone growth, abnormal mineral deposition, and metabolic factors, as illustrated in Figure 1.

### 3.1 Abnormal bone resorption

Abnormal bone resorption can be classified into physiological and pathological types, primarily associated with factors such as age, genetics, hormones, inflammatory responses, and lifestyle. For example, hormones such as parathyroid hormone and vitamin D influence osteoclast activity, thereby altering the bone resorption process. During the bone resorption phase, osteoclasts attach to the bone surface and secrete acids and proteases to degrade the bone matrix, leading to reduced bone mass and decreased bone strength. Subsequently, during the bone formation phase, osteoblasts migrate to the resorption site, secrete bone matrix, and promote its mineralization. These changes not only exacerbate bone metabolic disorders but may also affect the function of other organ systems, creating a vicious cycle. The RANKL/RANK/OPG is considered the core regulatory pathway controlling this process (Udagawa et al., 2002). Additionally, signaling pathways such as NF-κB and Ca^2+^-NFATc1 play crucial roles in regulating bone resorption (Russo et al., 2022).

### 3.2 Abnormal bone growth

Abnormal bone growth primarily involves osteoblast dysfunction. Under normal conditions, osteoblasts form a matrix containing collagen and proteoglycans, which gradually mineralizes into osteoid tissue. However, certain genetic disorders or metabolic abnormalities may impair osteoblast function, thereby affecting bone formation. The differentiation and function of osteoblasts are regulated by multiple signaling pathways, with the Wnt/β-catenin pathway playing a pivotal role. Wnt proteins bind to Frizzled receptors and LRP5/6 co-receptors, stabilizing β-catenin and promoting its nuclear translocation, which activates the expression of osteoblast-specific genes such as Runx2 and Osterix (Guo et al., 2021). Simultaneously, the BMP/Smad pathway regulates osteoblast differentiation through the phosphorylation and nuclear translocation of Smad1/5/8 (Fernandes and Gomes, 2016). Furthermore, signaling pathways such as Hedgehog, Notch, and FGF are also involved in osteoblast regulation. These pathways interact in complex ways to coordinate bone formation (Vlashi et al., 2023).

### 3.3 Abnormal mineral deposition

Abnormal mineral deposition is primarily characterized by disturbances in calcium and phosphorus metabolism. Calcium and phosphorus are the main metabolites of bone mineralization, and their metabolism is tightly regulated. In MBDs, various factors, such as vitamin D deficiency and parathyroid dysfunction, can disrupt calcium and phosphorus homeostasis. These disturbances directly affect bone mineralization, leading to reduced or abnormal mineral deposition. Recent studies have identified fibroblast growth factor 23 (FGF23) as a key regulator of mineral metabolism (Smith et al., 2014). FGF23, primarily produced by osteocytes, regulates phosphorus metabolism through a dual mechanism: on one hand, it inhibits phosphate reabsorption in the renal proximal tubules, and on the other hand, it suppresses the synthesis of 1,25-dihydroxyvitamin D (Sirikul et al., 2022). In patients with chronic kidney disease (CKD) and other MBDs, elevated FGF23 levels lead to hypophosphatemia and abnormal vitamin D metabolism, ultimately impairing normal mineral deposition and exacerbating skeletal pathology (Llorente-Pelayo et al., 2024).

### 3.4 Influence of metabolic factors

Metabolic disorders play a critical role in the development and progression of MBDs, with abnormalities in glucose, lipid, and protein metabolism having particularly significant impacts on bone metabolism. In terms of glucose metabolism, hyperglycemia affects bone metabolism balance through multiple mechanisms: on one hand, high glucose levels directly inhibit osteoblast differentiation and function while promoting osteoclast formation and activity (Sheu et al., 2024); on the other hand, the accumulation of advanced glycation end products, insulin resistance, and abnormalities in the insulin-like growth factor-1signaling pathway also contribute to glucose metabolism-related bone disorders (Liu et al., 2023; Mohan and Baylink, 1996). Lipid metabolism disorders not only induce osteoblast apoptosis and promote osteoclast differentiation, thereby inhibiting bone formation, but also alter the bone microenvironment by increasing bone marrow adipose tissue, ultimately disrupting bone remodeling balance (Zhang et al., 2024). Additionally, oxidized low-density lipoprotein promotes osteoclastogenesis by activating the NF-κB signaling pathway, further exacerbating bone resorption (Wang et al., 2022). Protein metabolism dysregulation affects bone metabolism primarily through bone matrix synthesis and osteocyte function. Inadequate protein intake reduces bone matrix synthesis, impairing bone mineralization, while abnormal amino acid metabolism alters osteocyte energy metabolism and signal transduction, thereby affecting bone remodeling (Srivastava et al., 2022). Notably, metabolic abnormalities of specific amino acids, such as arginine and glutamine, may directly influence the dynamic balance between bone formation and resorption (Devignes et al., 2022).

## 4 Application of omics technologies in the diagnosis of MBD

Omics technologies, as a pivotal technical system in systems biology research, integrate multidimensional data from transcriptomics, proteomics, and metabolomics to systematically elucidate dynamic changes in molecular networks within organisms. In the field of MBDs, these technologies not only comprehensively reveal the molecular mechanisms underlying bone metabolism abnormalities but also enable precise disease classification, dynamic monitoring of disease progression, and prognostic evaluation through biomarker screening. This provides novel perspectives and methodologies for clinical diagnosis and treatment.

Chen et al. conducted a systematic analysis of exosomal proteins in patients with OP at different disease stages using quantitative proteomics. They identified and validated four exosomal proteins—PSMB9, PCBP2, VSIR, and AARS—with diagnostic potential, offering new molecular targets for the clinical diagnosis of OP (Chen et al., 2020). Huo et al. employed a quantitative proteomics technologies to compare serum microvesicle protein expression profiles between OP patients and healthy controls, revealing significant differential expression of Vinculin, Filamin A, and Profilin 1. Notably, Profilin 1 expression was markedly elevated in the osteoporosis group, suggesting its potential as a valuable diagnostic indicator for OP. This finding provides critical experimental evidence for establishing a dynamic monitoring system for bone metabolism based on body fluid analysis (Huo et al., 2019). Wu Xiuhua et al. identified 165 differentially expressed genes, including Acot1, Mpig6b, Gp9, Ppbp, and Slc2a9, in a ketogenic diet-induced osteoporosis model compared to a normal diet group using transcriptomic sequencing. These genes are involved in signaling pathways such as Apelin, PI3K-Akt, and ECM-receptor interaction (Wu X. et al., 2023). Achim Buck et al. utilized spatial metabolomics to reveal complex metabolic alterations in Hyp mice, including significant changes in the pentose phosphate pathway, galactose metabolism, purine metabolism, ascorbate and aldarate metabolism, cysteine and methionine metabolism, pyrimidine metabolism, and arginine metabolism. Additionally, decreased levels of glycerophospholipids, phosphosphingolipids, fatty acids, amino acids, and peptides were observed, while carbohydrates, nucleic acids, vitamins, cofactors, and organic sulfates were elevated (Buck et al., 2022).

Notably, the application of omics technologies can not only facilitate the early diagnosis of MBDs but also provide a basis for MBDs classification by identifying distinct characteristics in metabolites, proteins, genes, and other molecular features.

The molecular characteristics uncovered by omics technologies allow for the classification of MBD into specific types, such as OP, osteomalacia, primary hyperparathyroidism, and Marfan syndrome. These classifications not only reflect the diverse etiologies of the diseases but also underscore the associated alterations in metabolites, proteins, and other molecular metabolites *in vivo*.

### 4.1 The most common MBD (OP)

OP is the most common MBD. OP is primarily classified into primary and secondary types based on etiology. Primary OP includes postmenopausal osteoporosis (PMOP), senile osteoporosis (SOP), and idiopathic osteoporosis. Secondary OP encompasses glucocorticoid-induced osteoporosis (GIOP), CKD-related osteoporosis, diabetic osteoporosis (DOP) and others.

#### 4.1.1 Senile osteoporosis

Yahui Wu et al. analyzed plasma from 379 elderly OP patients using untargeted liquid chromatography/gas chromatography-mass spectrometry (LC/GC-MS) and identified associations with retrograde endocannabinoid signaling, glycerophospholipid metabolism, steroid hormone biosynthesis, and xenobiotic metabolism by cytochrome P450. Among these, 2-aminomuconic acid semialdehyde showed diagnostic value in male OP, while tetradecanedioic acid exhibited diagnostic potential in female OP patients (Wu Y. et al., 2023). Xu Zhou et al. based on label-free quantitative proteomics (Easy-nLC1000 and Q-exactive) and Western blotting to discover that Abl Interactor 1 was significantly downregulated in elderly Chinese men with extremely low BMD. A similar trend was observed in elderly women. Functional studies demonstrated that Abl Interactor 1, at an optimal concentration of 2.0 ng/mL, significantly promoted osteoblast growth, expression of osteogenesis-related genes (OPN, ALP, COL1A1), and osteoblast differentiation. These findings suggest Abl Interactor 1 as a potential biomarker for diagnosing OP and osteoporotic fractures (Zhou et al., 2019).

#### 4.1.2 Postmenopausal osteoporosis

PMOP is primarily associated with declining estrogen levels (Li et al., 2020). When distinguishing OP between men and postmenopausal women, combining relevant metabolites with traditional bone turnover markers enhances diagnostic sensitivity, offering a foundation for early diagnosis and mechanistic studies of PMOP (Wang et al., 2019). Xin Li et al. analyzed metabolic interactions among the kidneys, bone marrow, and bones from a metabolomics perspective, identifying co-metabolites and pathways. Analysis of metabolites such as creatine, glutamine, β-hydroxybutyrate, and α-tocopherol further clarified the close relationship between energy metabolism, fatty acid metabolism, and ammonia metabolism (Li X. et al., 2022). Hongxia Zhao et al. conducted bone metabolomics-lipidomics using ultra-performance liquid chromatography-quadrupole time-of-flight mass spectrometry, revealing significant changes in fatty acyls, glycerolipids, glycerophospholipids, sphingolipids, and sterols. Disruptions in amino acid metabolism, nucleotide metabolism, and lipid metabolism were closely linked to the imbalance between bone resorption and formation, potentially underpinning PMOP (Zhao et al., 2018). Numerous metabolomics studies have shown that OP pathogenesis is closely related to amino acid metabolism, lipid metabolism, energy metabolism, and gut microbiota dysbiosis (Fan et al., 2021). Compared to healthy women, PMOP may involve disruptions in glucose, lipid, and amino acid metabolism, particularly associated with 18 metabolites, including isothreonic acid and ornithine, which are considered potential biomarkers for PMOP (Kou et al., 2022). A cross-sectional study of 517 perimenopausal and early postmenopausal women revealed associations between metabolites and BMD changes. Four fatty acids, three glycerophospholipids, three sterol lipids, two peptides, and eight other organic metabolites were significantly correlated with BMD changes. Notably, a functional metabolite module composed of fatty acids was closely related to BMD changes (Gong et al., 2021). Metabolomics has also played a significant role in TCM syndrome differentiation for OP. Compared to non-OP women, S-lactoylglutathione levels were significantly elevated in OP women with kidney-yang deficiency, suggesting its potential as a key indicator for TCM-based OP diagnosis (Yin et al., 2022).

#### 4.1.3 Glucocorticoid-induced osteoporosis(GIOP)

Glucocorticoids, commonly used anti-inflammatory drugs, directly or indirectly affect bone remodeling and are a leading cause of secondary OP (Hu and Adachi, 2019). They impair the osteogenic capacity of mesenchymal stem cells (MSCs) and promote their differentiation into adipocytes (Lane, 2019). Additionally, glucocorticoids inhibit osteoblast maturation, shorten their lifespan, reduce their function, and induce osteocyte apoptosis, ultimately leading to bone loss and OP (Weinstein, 2012). Using LC-MS metabolomics in a glucocorticoid-induced OP model, ovariectomy (OVX) alone or combined with glucocorticoids altered metabolite and lipid profiles in sheep, suggesting that phenylalanine, tyrosine, and tryptophan biosynthesis, as well as cysteine, methionine, and branched-chain amino acid metabolism, may be key pathways regulating bone loss in OVX sheep (Weinstein, 2012). Ying et al. employed transcriptomics to analyze GIOP characteristics, identifying 158 glucocorticoid-related candidate genes significantly enriched in OP-related pathways, providing new insights into the diagnosis and treatment of GIOP (Ying et al., 2020).

#### 4.1.4 Diabetic osteoporosis

DOP, a form of secondary OP, is characterized by reduced bone mineral content (BMC), decreased BMD, and bone structure deterioration due to hyperglycemia and metabolic alterations. Wang Yan et al. integrated dynamic contrast-enhanced magnetic resonance imaging Ktrans mapping texture analysis with metabolomics to evaluate early bone marrow microvascular lesions in diabetes, revealing associations with lipid metabolism abnormalities, particularly in linoleic acid metabolism (Wang Y. et al., 2024). W.-D. Liang et al. studied 18 DOP patients and found decreased levels of O-acetyl glycoprotein, proline, 1-methylhistidine, and tricarboxylic acid (TCA) cycle products, alongside elevated levels of branched-chain amino acids, choline, creatine, myo-inositol, glutamine, glutamate, aspartate, alanine, glycine, and citrulline. These changes may serve as early diagnostic markers for DOP (Liang et al., 2020). Kefeng Wu et al. observed significant increases in fatty acyls, glycerophospholipids, and phosphatidylethanolamines in T2DOP mice, with downregulated amino acid pathway metabolites. Dysregulation of lipid and glutathione pathways was identified as a major contributor to T2DOP progression in C57BKS mice (Wu et al., 2024).

#### 4.1.5 Renal osteodystrophy

Renal osteodystrophy, a MBD secondary to CKD, arises from calcium, phosphorus, and vitamin D metabolism disorders, secondary hyperparathyroidism, and acid-base imbalances. Clinical manifestations include OP, bone and joint pain, pathological fractures, and soft tissue and vascular calcification. The Wnt/β-catenin signaling pathway plays a crucial regulatory role in renal osteodystrophy, affecting the dynamic balance between bone formation and resorption (Wang et al., 2016). Aline L. Baptista et al. used nuclear magnetic resonance metabolomics to identify elevated levels of dimethyl sulfone, glycine, citrate, and N-acetylornithine in high bone turnover patients, while low ethanol levels were associated with abnormal bone mineralization, and low carnitine levels were linked to low bone volume. These findings aid in assessing bone phenotypes in chronic kidney disease-mineral and bone disorder (CKD-MBD) patients (Baptista et al., 2020). Other studies have shown that CKD-MBD pathogenesis is related to changes in protein synthesis and metabolism, amino acid metabolism, energy metabolism, and steroid hormone metabolism pathways, involving metabolites such as N-(1-Deoxy-1-fructosyl)tryptophan, glycylprolylhydroxyproline, and aminohippuric acid (Wu et al., 2015). Murat Kasap et al. identified seven protein spots in MSCs isolated from uremic bone disease patients with chronic renal failure using proteomics and molecular methods, discussing their potential relationships (Kasap et al., 2015).

### 4.2 Other MBDs

Although less studied, other MBDs have also been explored using omics technologies. Yuan Xiaozhou et al. employed magnetic bead-based weak cation exchange chromatography combined with matrix-assisted laser desorption/ionization time-of-flight mass spectrometry to identify four upregulated and three downregulated mass spectrometry peaks in mucopolysaccharidosis type I patients compared to healthy controls, offering a non-invasive diagnostic method for mucopolysaccharidosis type I (Yuan et al., 2017). Clarisse L. Torres et al. used untargeted liquid chromatography-high-resolution mass spectrometry metabolomics to reveal elevated levels of dipeptides, amino acids, and their derivatives, as well as N-acetylgalactosamine 4- or 6-sulfate (key metabolites of glycosaminoglycans) in mucopolysaccharidosis patients, providing new insights into its pathological mechanisms (Torres et al., 2023). Other MBDs, such as Marfan syndrome, have been less studied using omics technologies.

The application of omics technologies in the pathological classification and TCM syndrome differentiation of MBDs is still in its infancy. Current evidence-based medical data remain insufficient, highlighting the need for systematic and in-depth research. Such studies will provide molecular-level scientific evidence for TCM interventions in MBDs, facilitating precision medicine approaches.

## 5 Application of omics technologies in identifying potential targets of TCM monomers for treating MBD

**FIGURE 2:**
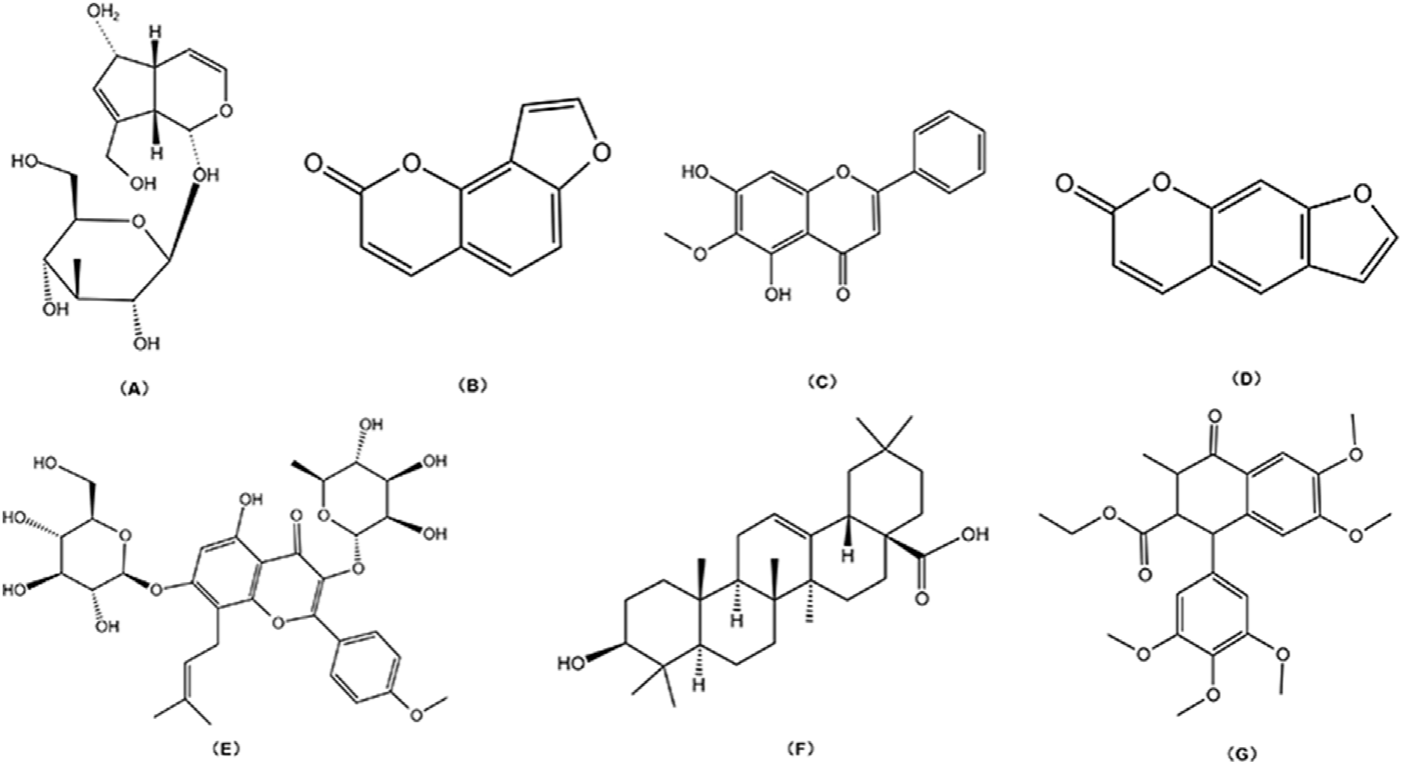
Chemical structural: **(A)** Aucubin; **(B)** Isopsoralen; **(C)** Oroxylin A; **(D)** Psoralen; **(E)** Icariin; **(F)** Oleanolic Acid; **(G)** Lignan.

TCM monomers, as the key active metabolites, are responsible for the pharmacological effects of TCM. Different botanical drugs may contain identical monomeric metabolites, which exhibit diverse medicinal properties and serve as crucial targets for TCM research and development. Table 1 summarizes the progress in metabolomics, proteomics, and transcriptomics studies of commonly used TCM monomers in treating MBDs. Figure 2 displays the chemical structures of common monomers.

**TABLE 1:** Application of omics in the study of anti-MBD mechanism of TCM monomer.

Monomer of TCM	Chemical formula	Models	Dosing time	Administration	Sample type	Target of action or pathway
Metabolomics						
Aucubin (Wang et al., 2024b)	C_15_H_22_O_9_	Dexamethasone induced OP in mice	4 weeks	5, 10, 20 mg/kg/d	Blood	Arachidonic acid
Isopsoralen (Liu et al., 2024)	C_11_H_6_O_3_	Dexamethasone -induce GIOP mice	4 weeks	5, 10, 20 mg/kg	Blood	Purine Metabolism
Achyranthes bidentata polysaccharides (Zhang et al., 2018)	(C_6_H_10_O_5_)n	OVX Rats	13 weeks	400 mg/kg body weight/day	Serum	Glycerophospholipid metabolism and retinol metabolism pathways
Oroxylin A (Xian et al., 2023)	C_16_H_12_O_5_	OVX Mice	6 weeks	2.5 mg/kg or 5 mg/kg every 2 days	Blood	phenylalanine, tyrosine, and tryptophan biosynthesis
lignan-rich fraction (Xiao et al., 2018)	C_25_H_30_O_8_	OVX Rats	12 weeks	57, 114, 228 mg/kg	Tibia	Biosynthesis of aminoacyl-tRNA, the biosynthesis of valine, leucine, and isoleucine, and the metabolic pathways for alanine, aspartate and glutamate
Transcriptomics						
Icariin (Gao et al., 2013)	C_33_H_40_O_15_	OVX Rats	13 weeks	aqueous phase 0.4 mL/(100 g⋅d), oil phase 0.6 mL/(100 g⋅d)	Thigh bone	175 genes were upregulated, 357 genes were downregulated; Sp3, Pctp, MMP8, and MT3 demonstrated the most substantial changes
Icariin (Bian et al., 2011)	C_33_H_40_O_15_	Rats with Corticosterone-induced OP	19 days	20 mg/kg/d	Bone marrow stromal cells	Gjb2, Cdh1, Cdkn2a, Alp1, Bmp10, Bmpr1a, Fgf21, Fgfr2, Adam17, Adar, Btrc
Psoralen (Bian et al., 2011)	C_11_H_6_O_3_	Rats with Corticosterone-induced OP	19 days	20 mg/kg/d	Bone marrow stromal cells	Cd4, Cdkn2a, Alpl, Adam17, Aph1a, Aph1b, Adar, Bmpr1a, Fgf21, Fgfr2, Igf1, Cd8b
Oleanolic Acid (Bian et al., 2011)	C_30_H_48_O_3_	Rats with Corticosterone-induced OP	19 days	20 mg/kg/d	Bone marrow stromal cells	Cdh1, Cd4, Cdkn2a, Alpl, Bmp6, Adam17, Aph1a, Aph1b, Numb, Btrc, Igf1, Bmpr1a, Fgf1, Fgf21, Col2a1
Lignan-rich fraction (Xiao et al., 2018)	C_25_H_30_O_8_	OVX Rat	12 weeks	57,114, 228 mg/kg	Tibia	OPG/RANKL
Morinda officinalis saponins (Zhou et al., 2024)	—	Human umbilical cord mesenchymal stem cells	21 days	(0, 5, 25, 50, 100, and 200 μg/mL) for 24, 48, 72, and 96 h	Human umbilical cord mesenchymal stem cells	BMP4, EDN1, KBTBD8, KIT,MYB,PRICKLE1, SEMA6A, SEMA6D, SOX6,SHC4 and SOX17; TGF-β/BMP-SMAD, Calcium, MAPK, PI3K-Aktsignaling pathway

### 5.1 Metabolomics

Metabolomics studies have demonstrated that TCM monomers can ameliorate bone metabolism imbalance by multidimensionally regulating metabolic networks. Icariin, one of the most widely used TCM monomers for MBDs, promotes osteoblast-mediated bone formation and inhibits osteoclast differentiation. It achieves this by regulating lipid metabolism, cytoskeleton remodeling, and energy metabolism homeostasis. These effects result in increased BMD and BMC, along with reduced levels of the bone resorption marker CTX-I. Icariin systematically improves the bone metabolic microenvironment by modulating energy metabolism, lipid metabolism, amino acid metabolism, phospholipid metabolism, and the gut microbiota axis. And this leads to increased bone mass (Pan et al., 2016). Metabolomics analysis based on UPLC/Q-TOF-MS revealed that puerarin improves osteoporosis and lipid metabolism in OVX rats. It specifically regulates phospholipid metabolism homeostasis and polyunsaturated fatty acid biosynthesis pathways, reducing adipogenic differentiation and promoting osteogenesis (Li B. et al., 2022). Aucubin, a major metabolite of Eucommia ulmoides Oliv. and Plantago asiatica L., promotes osteoblast differentiation and inhibits osteoclast formation in GIOP mice. It achieves this by regulating the arachidonic acid metabolism pathway (Wang H. et al., 2024). Isopsoralen significantly improves bone microstructure mechanics and strength parameters in GIOP mice. It activates the cGMP/PKG signaling axis to regulate purine metabolism homeostasis. Additionally, it increases serum calcium, phosphorus, and alkaline phosphatase levels, thereby promoting bone homeostasis reconstruction (Liu et al., 2024).

Mengliu Zhang et al. discovered that Achyranthes bidentata polysaccharides significantly increase biomarkers such as glutarylcarnitine, lysoPC (18:1), and 9-cis-retinoic acid through UPLC/Q-TOF-MS-based metabolomics. These changes improve BMD and trabecular bone structure by regulating lipid metabolism (Zhang et al., 2018). Oroxylin A, an active metabolites of Scutellaria baicalensis Georgi root, significantly improves bone density and trabecular structure in VOX mice. It specifically regulates the phenylalanine, tyrosine, and tryptophan biosynthesis pathways, affecting key metabolites such as L-phenylalanine, L-tryptophan, lysophosphatidylcholine (18:2), PC (22:5 (4Z, 7Z, 10Z, 13Z, 16Z)/16:1 (9Z)), and taurocholic acid (Xian et al., 2023).

### 5.2 Transcriptomics

Gao Feng et al. found that icariin significantly alters gene expression in ovariectomized rats, with 532 genes showing notable changes compared to the model group. Notably, Sp3, Pctp, MMP8, and MT3 were the most significantly altered genes. These genes potentially regulate lipid metabolism, signal transduction, transcription factor activity, collagen degradation, and immune responses, thereby improving osteoporosis (Gao et al., 2013). Bian Qin et al. used gene chip technology to compare the regulatory networks of icariin, psoralen, and oleanolic acid on bone marrow mesenchymal stem cells (BMSCs) in a corticosterone-induced rat model. They identified five common genes regulated by all three metabolites, including osteogenic differentiation-related molecules, cell cycle regulators, cytokines, and the Notch signaling pathway molecule Adam17. This suggests that kidney-tonifying TCM may promote BMSC osteogenic differentiation by regulating the cell cycle and metabolism, ultimately treating osteoporosis (Bian et al., 2011).

These studies reveal that TCM monomers exhibit multidimensional regulatory properties. Single metabolites can simultaneously act on gene expression, signal transduction, and the metabolic microenvironment related to bone metabolism. Homologous monomers exhibit distinct regulatory networks depending on the etiology, such as hormone deficiency or glucocorticoid excess. Furthermore, integrated multi-omics analysis (metabolomics-transcriptomics-proteomics) can systematically elucidate the cascade regulatory relationships of “metabolite -target-pathway-phenotype.” This is achieved through differential metabolite screening, key gene co-expression network construction, and pathway enrichment analysis. These findings provide a theoretical foundation and research paradigm for developing novel anti-MBD drugs based on the multi-target properties of TCM.

## 6 Application of omics technologies in identifying potential targets of single botanical drugs for treating MBD

TCM botanical drugs contain multiple active metabolites, enabling multi-target effects even without combination with other botanical drugs, effectively functioning as a small formula. In TCM practice, kidney-tonifying and bone-strengthening single botanical drugs are commonly used to treat MBDs. These botanical drugs not only ameliorate bone loss caused by metabolic disorders but also promote bone formation. Based on this, we summarize high-frequency single botanical drugs used for treating MBDs and elucidate their mechanisms from metabolomics, proteomics, and transcriptomics perspectives, as detailed in Table 2. In Traditional Chinese Medicine Systems Pharmacology Database and Analysis Platform (http://tcmspw.com/tcmsp.php), set the screening criteria to DL ≥ 0.18, OB ≥ 30%, Caco-2 ≥ −0.4 and HL ≥ 4 or HERB (http://herb.ac.cn/Search/), set the screening criteria to ingredient weight≤500 and inquire the drug metabolites of which is shown in Supplementary Table S1.

**TABLE 2:** Application of omics in the study of anti-MBD mechanism of single botanical drug.

botanical Drug	Subject of action	Dosing time	Administration	Sample type	Target of action or pathway
Metabolomics					
Cistanche deserticola Ma (Li et al., 2024a)	OVX Rats	12 weeks	0.6, 1.2 g/kg/d	Blood	Nucleotide, carbon, amino acid, and lipid metabolism pathways
Eucommia ulmoides Oliv. (Fang-Jie et al., 2020)	OVX Rats	3 months	0.3338 g/kg/d	Blood	Glycine, lysine, tryptophan, docosahexaenoic acid and glucose
Panax quinquefolius L. (Oiu et al., 2023)	Prednisolone-induced GIOP zebrafish	4 days	2.5, 5, 10 μg/mL	Zebrafish cubs	Purine metabolism, tricarboxylic acid cycle and pentose phosphate metabolism
Achyranthes bidentata Blume (Tao et al., 2019)	OVX Rats	12 weeks	75.6 g/kg/d	Serum, liver and kidney	Galactose metabolism, urea cycle, arginine and proline metabolism, alanine metabolism, lactose degradation, ammonia recycling and glycine and serine metabolism and so on
salt Eucommia ulmoides Oliv. (Shen et al., 2023)	5/6 nephrectomy-induced CKD-MBD mice and low calcium and high phosphorus diet	9 weeks	200, 600 mg/kg/d	fecal samples	Biosynthesis of phenylalanine, tyrosine, and tryptophan, Phenylalanine metabolism, Starch and sucrose metabolism, and galactose metabolism
Echinops davuricus Fisch. ex Hornem. (Dong et al., 2023)	D-galactose induced osteoporosis in rats	8 weeks	878, 439, 219.5 mg/kg/d	Serum	arachidonic acid metabolism, phenylalanine metabolism and tyrosine metabolism pathway
Glycine max (L.) Merr. (Sheng et al., 2020)	OVX Rats	8 weeks	2.7 g/kg/d	Urine	Steroid hormone biosynthesis, Ascorbate and aldarate metabolism, Pentose and glucuronate interconversions, Starch and sucrose metabolism and Inositol phosphate metabolism
Dipsacus asper Wall. Ex DC. (Tao et al., 2017)	OVX Rats	12 weeks	75.6 g/kg/d	Serum Liver and Kidney	Phenylalanine, tyrosine and tryptophan biosynthesis, valine, leucine and isoleucine biosynthesis, methane metabolism, glycine, serine and threonine metabolism, galactose metabolism and so on
Echinops davuricus Fisch. ex Hornem. (Wang et al., 2020)	OVX Rats	8 weeks	1.9 g/kg/d	Serum	Amino acids and glycerophospholipids metabolism
Gynochthodes officinalis (F.C.How) Razafim. & B.Bremer (Xia et al., 2019a)	Dexamethasone-induced GIOP rats	8 weeks	0.5, 1, 2 g/kg/d	Urine samples	Arachidonic acid metabolism, retrograde endocannabinoid signaling and amino acid metabolism
Cullen corylifolium (L.) Medik. (Wei et al., 2024)	OVX Mice	12 weeks	15, 45, 135 mg/kg/d	Blood	Arachidonic acid metabolism, biosynthesis of unsaturated fatty acids, linoleic acid metabolism, glycerophospholipid metabolism, purine metabolism and tryptophan metabolism
Rehmannia glutinosa (Gaertn.) Libosch. ex DC. (Xia et al., 2019b)	dexamethasone – induced rats	8 weeks	1, 2, 4 g/kg/d	Urine	steroid hormone biosynthesis, sex steroids regulation, and amino acid metabolism
Proteomics					
Eucommia ulmoides Oliv. (Zeng et al., 2010)	Mesenchymal stem cells	14 days	1 mL per dose, twice daily	Mesenchymal stem cells	Lamin A; Vimentin; Annexin A6; Superoxide dismutase; Stromal cell-derived factor; calreticulin
Drynaria roosii Nakaike (Su et al., 2024)	OVX Rats	12 weeks	0.48 g/kg/d	Bone tissue	estrogen signaling pathway, PPAR signaling pathway, erroptosis, and inflammatory immune system, PLTP, PON1, RAP1B, TFRC, MMP2, LGALS1, PIN1, PDGFRL SORBS1 and other related proteins
Achyranthes bidentata Blume (Yang et al., 2022)	Dexamethasone-induced GIOP rats	60 days	1 mg/kg/d	Femoral tissue	93 proteins such as oxidative phosphorylation, MT-CYB and NDUFA9
Drynaria roosii Nakaike (Jiang et al., 2020)	Retinoic acid-induced GIOP rats	60 days	10 g/kg/d	Serum	Arachidonic acid metabolism, glycerophospholipid metabolism and linoleic acid metabolic pathway
Transcriptomics					
Cistanche deserticola Ma (Tian et al., 2023)	OVX Rats	12 weeks	133.33 mg/kg/d	Bone	The myosin superfamily (MYH6, MYH7, MYL1, MYL3) and skeletal muscle (ACTN2, LDB3, CSRP3, MYOZ2, TNNT3, and CAV3)
Salt Eucommia ulmoides Oliv. (Shen et al., 2023)	5/6 nephrectomy-induced CKD-MBD mice + low calcium and high phosphorus diet	9 weeks	200, 600 mg/kg/d	kidney tissues	AMPK signaling pathway, cAMP signaling pathway, Insulin signaling pathway, PI3K/Akt signaling pathway, and other related pathways

### 6.1 Metabolomics

Classic kidney-tonifying botanical drugs (e.g., Rehmannia glutinosa (Gaertn.) Libosch. ex DC., Epimedium brevicornu Maxim., Drynaria roosii Nakaike, and Achyranthes bidentata Blume) maintain bone metabolic balance by regulating steroid hormone biosynthesis, phenylalanine/tyrosine metabolism, glycerophospholipid metabolism, and arachidonic acid metabolism. These botanical drugs upregulate metabolites such as p-coumaric acid, tyrosine, glucose, tagatose, and 2-hydroxycinnamic acid, while downregulating gluconic acid, adenylosuccinic acid, paxilline, ophthalmic acid, glycocyamine, lysine, and biotin (Ping et al., 2022; Huang et al., 2014; Li J. et al., 2024). Eucommia ulmoides Oliv. significantly reverses abnormal expression of glycine, lysine, docosahexaenoic acid, and glucose in the serum of ovariectomized osteoporotic rats by regulating amino acid metabolism and oxidative stress pathways, while restoring tryptophan levels (Fang-Jie et al., 2020). Qiu Yuezhi et al. found that Panax quinquefolius L. effectively improves abnormal expression of osteoblast- and osteoclast-related genes in a GIOP zebrafish model by participating in purine metabolism, the TCA cycle, and the pentose phosphate pathway, modulating 10 key biomarkers (Oiu et al., 2023). Achyranthes bidentata Blume protects against osteoporosis in rats by regulating galactose metabolism, the urea cycle, arginine and proline metabolism, alanine metabolism, lactose degradation, ammonia recycling, and glycine and serine metabolism, promoting the restoration of biomarkers to normal levels (Tao et al., 2019).

### 6.2 Proteomics

Zeng Jianchun’s team systematically analyzed the mechanism of Eucommia ulmoides Oliv. in inducing osteogenic differentiation of MSCs using two-dimensional electrophoresis. They identified 641 differentially expressed protein spots, demonstrating that Eucommia ulmoides Oliv. Primarily promotes cell differentiation, participates in bone mineralization, and regulates cell proliferation and differentiation through six functional proteins, significantly improving osteoporosis (Zeng et al., 2010). Hui Su et al. used TMT-labeled quantitative proteomics to reveal that Drynaria roosii Nakaike reverses osteoclast differentiation and enhances osteogenic activity by regulating mitochondrial function, RNA and protein receptor signaling, while inhibiting oxidative stress, improving lipid metabolism, and reducing inflammation, forming a dual regulatory mechanism for bone metabolism (Su et al., 2024). Additionally, Liu Yang et al. found that Achyranthes bidentata Blume extract significantly improves bone density and trabecular fractures in GIOP rats, enhances osteoblast differentiation, and inhibits osteoclast-mediated collagen dissolution. TMT-based proteomics identified 93 differentially expressed proteins (61 upregulated and 32 downregulated), primarily related to oxidative phosphorylation pathways, transmembrane transport, exocytosis, cell development, and ATP metabolism. MT-CYB and NDUFA9 were identified as potential biomarkers (Yang et al., 2022).

### 6.3 Transcriptomics

Shuo Tian et al. discovered that Cistanche deserticola Ma improves bone metabolic disorders in OVX rats by regulating skeletal muscle function, calcium homeostasis, adipogenic-osteogenic capacity, and osteoblast differentiation, potentially related to the myosin superfamily (MYH6, MYH7, MYL1, MYL3) and skeletal muscle (ACTN2, LDB3, CSRP3, MYOZ2, TNNT3, CAV3) (Tian et al., 2023). Salt-processed Eucommia ulmoides Oliv. may improve renal injury and bone microstructure in CKD-MBD mice by affecting the AMPK, cAMP, insulin, and PI3K/Akt signaling pathways, promoting trabecular bone growth and improving femoral tissue morphology (Shen et al., 2023).

These studies demonstrate that various single botanical drugs, despite their differences, can improve bone metabolism by regulating the expression or transcriptional regulation patterns of the same genes, highlighting the metabolomic characteristics of single botanical drugs in treating MBDs.

## 7 Application of omics technologies in identifying targets of Chinese herbal formula for treating MBD

In TCM theory, formulations are carefully designed based on syndrome differentiation principles. Botanical drugs in a formulation have distinct roles as “monarch,” “minister,” “assistant,” and “guide,” working synergistically or antagonistically to address different aspects of disease progression and exert comprehensive therapeutic effects through multiple pathways and targets.

In the treatment of MBDs, many classic formulations are widely used, such as Du-Huo Ji Sheng Tang, Zuogui Wan, and Er-Xian Tang. These formulations effectively maintain the dynamic balance of bone metabolism by promoting bone mineralization, alleviating clinical symptoms, and significantly improving therapeutic outcomes. Current research on the omics mechanisms of Chinese herbal formula in treating MBDs is summarized in Table 3, providing critical insights into their therapeutic principles and clinical optimization. The drug metabolites of Chinese herbal formula is shown in Supplementary Table S2.

**TABLE 3:** Application of omics in the study of anti-MBD mechanism of Chinese herbal formula.

TCM formula	TCM	Subject of action	Dosing time	Administration	Sample type	Target of action or pathway
Metabolomics						
Gushukang capsules (Lin et al., 2022)	Epimedium brevicornu Maxim., Rehmannia glutinosa (Gaertn.) Libosch. ex DC., Drynaria roosii Nakaike, Astragalus mongholicus Bunge, Salvia miltiorrhiza Bunge, Auricularia auricula (L.ex Hook) Underw	OVX Rats	12 weeks	0.32 g/kg/d	Lumbar vertebra	Cant1, Gstz1, Aldh3b1, Bid and Slc1a3, nucleotide metabolism, amino acid metabolism, immune processes, and general cellular processes
Zishen Jiangtang Pill (Li et al., 2018)	Astragalus mongholicus Bunge, Radix Rehmanniae Recens, Rehmannia glutinosa (Gaertn.) Libosch. ex DC., Schisandra chinensis (Turcz.) Baill., Epimedium brevicornu Maxim Cibotium barometz (L.) J.Sm., and Plastrum Testudinis, Curculigo orchioides Gaertn., Codonopsis pilosula (Franch.) Nannf., Achyranthes bidentata Blume, Drynaria roosii Nakaike, Panax notoginseng (Burkill) F.H.Chen Polygonatum sibiricum Redouté	STZ Rats	8 weeks	1.5, 3.0 g/kg/d	Blood and Urine	Acetate, urea, acetone, and citrulline
Shuanghuangyigu Recipe (Xiong et al., 2014)	Eucommia ulmoides Oliv., Rehmannia glutinosa (Gaertn.) Libosch. ex DC., Astragalus mongholicus Bunge, Dipsacus asper Wall. Ex DC., Drynaria roosii Nakaike, Siphonostegia chinensis Benth., Lycium barbarum L., Cornus officinalis Siebold & Zucc., Ophrys apifera Huds., Angelica sinensis (Oliv.) Diels, Achyranthes bidentata Blume	Patients with diabetes mellitus combined with MBD	3 months	—	Serum	Citrulline, Valine,Betaine Choline, Proline,Glutamine, O-Acetyl glycoprotein,N-Acetyl glycoprotein, Pyruvate,1-Methylhistidine, α-Ketoglutarate,Citrate, Leucine, Isoleucine,Glucose, Tyrosine, Alanine
Wen-Shen-Tong-Luo-Zhi-Tong-Decoction (Li et al., 2025)	Aconitum carmichaelii Debeaux, Cornus officinalis Siebold & Zucc., Drynaria roosii Nakaike, Epimedium brevicornu Maxim., Cnidium monnieri (L.) Cusson, Cibotium barometz (L.) J.Sm., Coix lacryma-jobi var. Ma-yuen (Rom.Caill.) Stapf, Atractylodes macrocephala Koidz Hansenia weberbaueriana (Fedde ex H.Wolff) Pimenov & Kljuykov, Angelica biserrata (R.H.Shan & C.Q.Yuan) C.Q.Yuan & R.H.Shan, Asarum heterotropoides F.Schmidt, Gastrodia elata Blume, Paeonia lactiflora Pall., Glycyrrhiza glabra L	Senile osteoporosis mice	8 weeks	0.21, 0.42 g/10 g/d	Blood	Central carbon metabolism, steroidogenesis, cholesterol metabolism, and the TCA cycle
Shenyang Fang (Huang et al., 2023b)	Cistanche deserticola Ma, Epimedium brevicornu Maxim., Drynaria roosii Nakaike	OVX Rats	60 days	5.14 g/kg/d	Blood	Amino acid and lipid metabolism
Erzhi Wan (Shang et al., 2024)	Ligustrum lucidum W.T.Aiton, Eclipta prostrata (L.) L.	OVX Rat and a mixed solution of thyroxine and reserpine	12 weeks	1 mL/100 g/d	Kidney, Bone marrow and bone	Arginine and proline metabolism, glycine, serine and threonine metabolism, alanine, aspartate and glutamate metabolism
Gushudan (Feng et al., 2022)	Epimedium brevicornu Maxim., Drynaria roosii Nakaike, Cnidium monnieri (L.) Cusson, Salvia miltiorrhiza Bunge	Hydrocortisone-induced GIOP rats	30 days	1.2 g/kg/d	Serum	Carbohydrate metabolism, amino acid metabolism and intestinal flora metabolism pathway
Yi-Guan-Jian decoction (Yin et al., 2023)	Rehmannia glutinosa (Gaertn.) Libosch. ex DC. , Glehnia littoralis (J.G.Cooper) F.Schmidt ex Miq, Angelica sinensis (Oliv.) Diels, Ophiopogon japonicus (Thunb.) Ker Gawl., Lycium barbarum L., Melia azedarach L.	Dexamethasone-induced GIOP mice	8 weeks	2 mg/kg/d	Liver	Taurine metabolic and hypotaurine metabolic
Roucongrong Pill (Jiang et al., 2025)	Cistanche deserticola Ma, Cuscuta chinensis Lam. ,Aconitum carmichaelii Debeaux, Achyranthes bidentata Blume, Cervus nippon Temminck	OVX Rats	12 weeks	8.5 g/kg	Fecal	Taurine and hypotaurine metabolism, phenylalanine Metabolism, and steroid hormone biosynthesis
Jianguwan (Chang et al., 2023)	Cistanche deserticola Ma, Dioscorea futschauensis Uline ex R.Knuth, Cuscuta chinensis Lam., Eucommia ulmoides Oliv., Pork Kidney	OVX Rats	12 weeks	3.6 g/kg	Blood	Arachidonic acid metabolism, inositol phosphate metabolism, arginine and proline metabolism, galactose metabolism and phosphatidylinositol signal transduction system
Jiangu granule (Liu et al., 2021)	Epimedium brevicornu Maxim., Cornus officinalis Siebold & Zucc., Dioscorea oppositifolia L., Codonopsis pilosula (Franch.) Nannf., Drynaria roosii Nakaike, Lycium barbarum L., Citrus reticulata Blanco, Curcuma longa L., Croci Stigma, Pyrola calliantha Andres	OVX Rats	12 weeks	5.8 g/kg/d	Blood	Pantothenate and CoA biosynthesis; Primary bile acid biosynthesis; Steroid hormone biosynthesis; Biosynthesis of unsaturated fatty acids; Pyrimidine metabolism; Biosynthesis of secondary metabolites
Jintiange Capsule (Fang et al., 2022)	Artificial Tiger Bone Powder	OVX Rats	12 weeks	180, 360 mg/kg	Blood	Glycerol phospholipid metabolism, aminoacyl-tRNA biosynthesis, Vitamin B6 metabolism and tryptophan metabolism pathways
Gushudan (Yuan et al., 2020)	Epimedium brevicornu Maxim., Drynaria roosii Nakaike, Cnidium monnieri (L.) Cusson, Salvia miltiorrhiza Bunge	Prednisolone-induced GIOP rats	12 weeks	30 g/kg	Plasma samples	Propanoate metabolism, Valine, leucine and isoleucine Biosynthesis, Glycerophospholipid metabolism, Phenylalanine metabolism, Arginine and proline metabolism, Primary bile acid biosynthesis, D-Glutamine and D-glutamate metabolism, Taurine and hypotaurine metabolism
Gushudan (Liu et al., 2020)	Epimedium brevicornu Maxim., Drynaria roosii Nakaike, Cnidium monnieri (L.) Cusson, Salvia miltiorrhiza Bunge	Prednisolone-induced GIOP Rats	12 weeks	30 g/kg/d	Urine samples	the energy metabolism, intestinal flora metabolism, amino acid metabolism, and oxidative stress response
Proteomics						
Shengmai Chenggu Capsule (Wang et al., 2006)	Leaf of Cajan, etc	OVX Rats	6 weeks	—	Bone tissue	Thioredoxin peroxidase 1; Myosin light polypeptide 2; ubiquitin-conjugating enzyme E2-17kD
Gushukang granule (Lin et al., 2021)	Epimedium brevicornu Maxim., Rehmannia glutinosa (Gaertn.) Libosch. ex DC., Drynaria roosii Nakaike, Astragalus mongholicus Bunge Salvia miltiorrhiza Bunge, Auricularia auricula (L.ex Hook) Underw	OVX Rats	12 weeks	4.32 g/kg/d	Lumbar vertebra	tyrosine metabolism, ethanol metabolism, JAK-STAT and other signal pathways
Er-xian Decoction (Liu et al., 2019)	Curculigo orchioides Gaertn., Epimedium brevicornu Maxim., Gynochthodes officinalis (F.C.How) Razafim. & B.Bremer, Angelica sinensis (Oliv.) Diels, Anemarrhena asphodeloides Bunge, Phellodendron chinense C.K.Schneid	OVX Rats	90 days	12 g/kg/d	Femur	Cytochrome b-245 light chain, Mitogen-activated protein kinase 1, Ras- related C3 botulinum toxin substrate 1
Zhuanggu Zhitong Formula (Yang et al., 2012)	Epimedium brevicornu Maxim., Cullen corylifolium (L.) Medik., Cibotium barometz (L.) J.Sm., Achyranthes bidentata Blume, Drynaria roosii Nakaike, Lycium barbarum L., Ligustrum lucidum W.T.Aiton	OVX Rats	13 weeks	0.6 mL of aqueous phase +0.4 mL of oil phase per 100 g, once daily	Femur	Creatine kinase M-type, Serotransferrin; Fructose-bisphosphate aldolase A,
Yigu decoction (Tang et al., 2023)	Cullen corylifolium (L.) Medik., Drynaria roosii Nakaike, Epimedium brevicornu Maxim., Salvia miltiorrhiza Bunge, Dioscorea oppositifolia L. Rehmannia glutinosa (Gaertn.) Libosch. ex DC.	OVX Rats	12 weeks	10 mL/kg/d	Femur	Transcriptional misregulation in cancer and Mineral absorption pathway
Zuogui Pill (Zhang et al., 2016)	Rehmannia glutinosa (Gaertn.) Libosch. ex DC., Dioscorea oppositifolia L., Lycium barbarum L Cornus officinalis Siebold and Zucc., Achyranthes bidentata Blume, Cuscuta chinensis Lam., Glue of Tortoise Plastron, Deerhorn Glue	OVX Rats	3 months	9.68 g/kg	Femur	Alpha-enolase
Jiangu granules (Li et al., 2024b)	Epimedium brevicornu Maxim., Cornus officinalis Siebold and Zucc., Dioscorea oppositifolia L., Codonopsis pilosula (Franch.) Nannf., Drynaria roosii Nakaike, Lycium barbarum L., Citrus reticulata Blanco, Curcuma longa L., Croci Stigma, Pyrola calliantha Andres	OVX Rats	12 weeks	—	Bone tissue	Steroid and Notch pathways; PHGs (PACS1, SRPK1, CYBB, RBPJ) and PPGs (AGL, CDH13, F7, FABP3)
Transcriptomics						
Zhuanggu Zhitong Formula (Chen et al., 2021)	Epimedii Foliu Psoralea, Psoraleae Fructus, Ligustri Lucidi Fructus, Babury Wolfberry Fruit, Drynariae Rhizoma, Achyranthes Bidentata, Cibotii Rhizoma	OVX Rats	13 weeks	567 mg/kg	Peripheral Blood Mononuclear Cells	Nlrp12, Oscar, LOC100359515,Csf3r,TNF signaling pathway, IL-17 signaling pathway and *Salmonella* infection signaling pathway
Bajitianwan formula (Xu et al., 2024)	Panax ginseng C.A.Mey., Polygala tenuifolia Willd., Ophrys apifera Huds., Lycium chinense Mill., Acorus verus (L.) Raf., Gynochthodes officinalis (F.C.How) Razafim. and B.Bremer	Iron overloaded OP mice	12 weeks	9, 18 g/kg/d	Hippocampus tissues, Serum, Liver	RAGE/PI3K/AKT pathway
Heng-Gu-Gu-Shang-Yu-He-Ji (Xie et al., 2024)	Panax ginseng C.A.Mey., Carthamus tinctorius L., Panax notoginseng (Burkill) F.H.Chen, Astragalus mongholicus Bunge, Datura metel L, Eucommia ulmoides Oliv., Citrus reticulata Blanco, Hydrangea ampla (Chun) Y.De Smet & Granados, Trionycis Carapax	OVX Rats	10 weeks	0.4395 g/kg/d	Femurs	PI3K/AKT/mTORC1 signaling pathway,94 genes as key regulatory genes, including Sirt7, Nog, etc.
Bushen Huoxue decoction (Mai et al., 2023)	Rehmannia glutinosa (Gaertn.) Libosch. ex DC., Cullen corylifolium (L.) Medik., Cuscuta chinensis Lam., Eucommia ulmoides Oliv., Lycium barbarum L., Angelica sinensis (Oliv.) Diels, Cornus officinalis Siebold & Zucc., Cistanche deserticola Ma, Angelica biserrata (R.H.Shan and C.Q.Yuan) C.Q.Yuan and R.H.Shan, Carthamus tinctorius L., Commiphora myrrha (T.Nees) Engl	OVX Rats	3 months	55 mg/kg/d	Femur	TLR4, MyD88, NF-kB
Bushen Jianpi Huoxue Formula (Chen et al., 2008)	Cullen corylifolium (L.) Medik., Paeonia lactiflora Pall., Epimedium brevicornu Maxim., Cistanche deserticola Ma, Rehmannia glutinosa (Gaertn.) Libosch. ex DC., Astragalus mongholicus Bunge, Cuscuta chinensis Lam., Angelica sinensis (Oliv.) Diels, Salvia miltiorrhiza Bunge, Ziziphus jujuba Mill	patients with primary OP	6 months	Take one dose daily, three times a day	Blood	BMP, Caspase3, Ca28 Tg, Ctla4, IL-6, TNf-α, SMAD2, TGF-β
Bushen Huatan Recipe (Zhang et al., 2022)	Cuscuta chinensis Lam., Epimedium brevicornu Maxim., Cullen corylifolium (L.) Medik., Trichosanthes kirilowii Maxim., Monascus purpureus, Crataegus monogyna Jacq	OVX Rats	12 weeks	9.4 g/kg/d	Blood	Steroid hormone biosynthesis, PI3K-Akt signaling pathway, Toll-like receptor signaling pathway,T cell receptor signaling pathway, TNF signaling pathway,HIF-1 signaling pathway, Osteoclast differentiation
Bu-Shen-YiQi Decoction (Zhong et al., 2023)	Epimedium brevicornu Maxim., Astragalus mongholicus Bunge, Rehmannia glutinosa (Gaertn.) Libosch. ex DC., Scutellaria baicalensis Georgi, Paeonia lactiflora Pall	Cigarette smoke-induced COPD-Related OP Rats	15 weeks	6.25 g/kg, 12.5 g/kg, 25 g/kg	Blood	MAPK and PI3K/AKT pathways
Yiwei Decoction (Fan et al., 2023)	Codonopsis pilosula (Franch.) Nannf., Atractylodes macrocephala Koidz., Ophrys apifera Huds., Glycyrrhiza glabra L., Citrus reticulata Blanco, Dolomiaea costus (Falc.) Kasana & A.K.Pandey, Wurfbainia villosa (Lour.) Škorničk. & A.D.Poulsen, Pinellia ternata (Thunb.) Makino	Cyclophosphamide-induced GIOP Rats	4 weeks	7.830 g/kg/d	Blood	ALB, C3, SNCA, and CLTC, Calcium signaling, growth hormone synthesis, secretion, and action, and the renin-angiotensin system
Liuwei DihuangPill (Chen et al., 2023)	Rehmannia glutinosa (Gaertn.) Libosch. ex DC., Dioscorea oppositifolia L., Alisma plantago-aquatica subsp. Orientale (Sam.) Sam., Ophrys apifera Huds., Paeonia × suffruticosa Andrews	OVX Rats	3 months	157.5 mg/kg/d	Femur	NLRP3, Caspase-1, GSDMD, IL-6,IL-1B, TNF-a, Bcl-2, Casepase-3Casepase-8, P53

### 7.1 Metabolomics

Chinese herbal formula primarily regulate bone metabolism by modulating amino acid metabolism and unsaturated fatty acid pathways to enhance osteoblast activity. Gushukang Capsule, a classic anti-OP formula listed in the Chinese Pharmacopoeia, consists of Epimedium brevicornu Maxim., Rehmannia glutinosa (Gaertn.) Libosch. ex DC., Drynaria roosii Nakaike., Astragalus mongholicus Bunge, Salvia miltiorrhiza Bunge., and Auricularia auricula (L.ex Hook.) Underw. This formulation achieves bone metabolic homeostasis through multi-target synergistic regulation: at the protein level, it corrects abnormal expression of key functional proteins such as Cant1 (calcium signaling regulator), Gstz1 (glutathione transferase), Aldh3b1 (aldehyde dehydrogenase), Bid (apoptosis regulator), and Slc1a3 (glutamate transporter); at the metabolite level, it systematically regulates the bioavailability of 12 bone metabolism-related substances, including tyramine, thymidine, deoxycytidine, cytosine, and L-aspartic acid; and by intervening in 11 core metabolic pathways, including purine metabolism, pyrimidine metabolism, histidine metabolism, and β-alanine metabolism, it significantly improves bone density and trabecular structure parameters (thickness, number, and connectivity density) in OVX model rats, validating the scientific basis of the TCM theory “kidney governs bone” and “tonifying the kidney to strengthen bone” (Lin et al., 2022).

DOP, with its multi-system involvement, is traditionally attributed to kidney essence deficiency, spleen dysfunction, and blood stasis, involving energy metabolism imbalance, impaired bone matrix synthesis, and microcirculation abnormalities (Liang and Xiong, 2013). Modern studies show that different Chinese herbal formula can achieve precise intervention through specific metabolic pathways. Huilin Li et al. found that Zishen Jiangtang Pill improves glucose metabolism, bone metabolism, and metabolic disorders in DOP mice by reducing levels of metabolic markers such as acetate, urea, acetone, and citrulline in the blood (Li et al., 2018). Shuang Huang Yi Gu Fang improves bone trabecular structure in DOP patients by upregulating citrulline, valine, betaine, choline, proline, glutamine, O-acetyl glycoprotein, N-acetyl glycoprotein, pyruvate, 1-methylhistidine, and TCA cycle products (α-ketoglutarate, citrate), while downregulating branched-chain amino acids (leucine, isoleucine), glucose, tyrosine, and alanine, thereby regulating bone proteins and treating the disease (Xiong et al., 2014). Wen Shen Tong Luo Zhi Tong Decoction effectively reverses bone loss in steroid-induced osteoporosis model mice by targeting central carbon metabolism, steroidogenesis, cholesterol metabolism, and the TCA cycle, improving bone microstructure and homeostasis, increasing BMD, bone volume/total volume, trabecular number, and trabecular thickness, and reducing trabecular separation (Li et al., 2025).

Chinese herbal formula can also treat MBDs based on syndrome differentiation. Huang Chen et al. used Shen Yang Fang to treat kidney-yang deficiency OP mice, showing that the formulation corrects amino acid and lipid metabolism disorders by significantly regulating 98 metabolites (89 upregulated and 9 downregulated) (Huang C. et al., 2023). Er Zhi Wan improves bone strength and microstructure in kidney-yin deficiency osteoporotic rats by regulating arginine and proline metabolism, glycine, serine, and threonine metabolism, and alanine, aspartate, and glutamate metabolism, while alleviating kidney-yang deficiency symptoms (Shang et al., 2024). Additionally, Gushudan prevents and treats kidney-yang deficiency OP in rats through multiple targets, with 11 differential metabolites, including N,N-dimethylglycine, guanidinoacetic acid, and glycolic acid, identified for the first time (Feng et al., 2022). Yi Guan Jian decoction, a classic formula for liver-kidney yin deficiency, increases BMD, improves bone microstructure, and reverses bone loss in GIOP mice by regulating taurine and hypotaurine metabolism and improving alkaline phosphatase and osteocalcin levels (Yin et al., 2023).

These studies collectively demonstrate that Chinese herbal formula dynamically regulate bone metabolism through synergistic multi-pathway effects, providing new theoretical foundations for precision medicine in MBDs.

### 7.2 Proteomics

Wang Haibin et al. identified ubiquitin-conjugating enzyme as an estrogen-related protein involved in disease progression and reported the biological functions of thioredoxin peroxidase 1 and myosin light polypeptide 2 as novel osteoporosis-related proteins. Notably, Shengmai Cheng gu Capsule significantly regulates bone metabolism by modulating the expression levels of these proteins (Wang et al., 2006). Gushukang granule improves BMD and regulates bone metabolism in OP model rats by regulating differential proteins and pathways such as tyrosine metabolism, ethanol metabolism, and JAK-STAT signaling (Lin et al., 2021). Liu Bo et al. systematically analyzed the anti-osteoporosis targets of the classic formula Er Xian Tang using iTRAQ isotope labeling combined with NanoLC-LTQ-Orbitrap high-resolution mass spectrometry, validating the dynamic regulation of key differential proteins such as cytochrome b-245 light chain, MAP kinase-1, and RAC1 during TCM intervention (Liu et al., 2019).

### 7.3 Transcriptomics

Recent advances in transcriptomics have significantly contributed to understanding the mechanisms of Chinese herbal formula in treating osteoporosis. Multiple studies show that traditional Chinese herbal formula regulate bone metabolic balance through multi-pathway synergistic effects. Zhuanggu Zhitong Formula significantly affects the expression of 149 genes in ovariectomized rats, with Nlrp12, Oscar, LOC100359515, and Csf3r playing key roles in osteoblast and osteoclast differentiation, suggesting that the formulation improves osteoporosis by simultaneously regulating osteoblasts and osteoclasts through TNF and IL-17 signaling pathways (Chen et al., 2021). Bajitianwan Formula improves bone microstructure in iron overload-induced osteoporosis by activating the RAGE/PI3K/AKT pathway and delaying oxidative stress (Xu et al., 2024). Notably, kidney-tonifying formulations exhibit multidimensional regulatory advantages: Heng-Gu-Gu-Shang-Yu-He-Ji significantly enhances bone biomechanical properties in OVX rats by activating the PI3K/AKT/mTORC1 pathway through 94 key genes, including Sirt7 and Nog (Xie et al., 2024). Bushen Huoxue decoction increases BMC and density, promotes bone formation, and regulates bone metabolism by downregulating TLR4, MyD88, and NF-κB expression and inhibiting inflammatory cytokines such as IL-6, IL-1β, and TNF-α(96). Bu Shen Jian Pi Huo Xue Formula reshapes bone remodeling balance by regulating 18 differentially expressed genes and over 20 signaling pathways, involving mechanisms such as upregulation of osteoblast proliferation genes and inhibition of osteoclast-related genes (Chen et al., 2008). In terms of immune regulation, Zhang Yu et al. found that Bushen Huatan Recipe regulates bone immunity and inflammation through Toll-like/T cell receptor signaling, TNF/HIF-1 signaling, and osteoclast differentiation pathways, achieving prevention and treatment of PMOP by participating in osteoblast/osteoclast differentiation, proliferation, and apoptosis, providing a reference for treating PMOP from the perspective of phlegm (Zhang et al., 2022). Yuanyuan Zhong et al. discovered that Bu-Shen-Yi-Qi Decoction improves chronic obstructive pulmonary disease-related osteoporosis by upregulating genes related to antioxidant stress and aerobic respiration and activating the MAPK and PI3K/AKT pathways (Zhong et al., 2023). Yiwei decoction, an effective formula for yangming deficiency closely related to OP, reduces gonadotropin-releasing hormone and follicle-stimulating hormone levels, decreases bone loss, promotes osteoblast formation, inhibits osteoclast formation, balances bone metabolism, and increases BMD in rats with ovarian insufficiency-related OP by regulating calcium signaling, growth hormone synthesis, secretion, and action, and the renin-angiotensin system, as well as key targets such as ALB, C3, SNCA, and CLTC (Fan et al., 2023).

These studies collectively reveal the multi-level characteristics of Chinese herbal formula in treating MBDs: (1) bidirectional regulation of bone metabolism through key signaling pathways (e.g., PI3K/AKT, MAPK); (2) reshaping the bone microenvironment by regulating specific gene expression (e.g., Sirt7, Nog); (3) improving the bone immune microenvironment by inhibiting inflammatory cytokine release; and (4) restoring bone metabolic homeostasis by regulating endocrine axis function. These findings provide molecular biological evidence for the modernization of TCM, but further exploration is needed to understand the synergistic mechanisms between different formulations and the spatiotemporal-specific regulation of key targets.

## 8 Discussion and conclusion

In recent years, with the rapid advancement of omics technologies, metabolomics, proteomics, and transcriptomics have been extensively applied in the research of MBDs. These technologies have comprehensively elucidated the pathogenesis of MBDs at the levels of metabolites, proteins, and genes, and have deeply explored the mechanisms of intervention by TCM monomers, single botanical drugs, and Chinese herbal formula, providing new insights and scientific evidence for the prevention and treatment of MBDs.

### 8.1 Advantages and disadvantages of TCM in preventing and treating MBD

Omics technologies can comprehensively reflect the multi-metabolite, multi-target, and multi-pathway characteristics of TCM, which aligns with the holistic principles of TCM. Moreover, omics technologies enable researchers to reveal the mechanisms underlying the active metabolites of TCM and their regulatory effects on bone metabolism-related genes, proteins, and metabolites. By leveraging the technical advantages of omics and the therapeutic potential of TCM, researchers can significantly advance the systematic and in-depth analysis of TCM efficacy mechanisms, integrating “molecular networks-pharmacological activities-disease-syndrome effects” at multiple levels. However, TCM emphasizes syndrome differentiation and individualized treatment, while omics research often struggles to fully capture the complexity of personalized therapies. Additionally, it is challenging to correlate omics data analysis results with clinical symptoms and therapeutic outcomes. Therefore, strengthening the integration of omics technologies with TCM is crucial to enhance the clinical relevance and practical utility of data analysis.

### 8.2 Literature quality assessment

We included high-quality studies published in both Chinese and English, which provide comprehensive details on the materials and methods used in the research process, particularly those pertaining to TCM and its metabolites. Specific aspects, including experimental models, administration methods, dosing schedules, and drug application sites, are thoroughly documented, thereby ensuring the reproducibility and reliability of the research findings. However, most of these studies are confined to animal experiments or *in vitro* cell models and lack relevant clinical trials. This limitation hinders the further validation of their precise efficacy and potential adverse effects.

### 8.3 Limitations and prospects

However, current research still faces several limitations: Firstly, limited scope of disease research: existing studies primarily focus on common MBDs such as OP, with less attention paid to relatively rare MBDs like osteomalacia, mucopolysaccharidosis, and Marfan Syndrome. Future research should expand the application of omics technologies to strengthen the study of other types of MBDs. Secondly, insufficient multi-omics integration analysis: current research predominantly employs single omics technologies, making it difficult to fully reveal the complex molecular regulatory networks of MBDs. Future efforts should enhance the combined use of metabolomics, proteomics, and genomics to construct a more comprehensive molecular mechanism map of MBDs. Thirdly, inadequate capacity for big data analysis: The vast amount of data generated by omics studies presents substantial challenges in collection, organization, and analysis, developing efficient and precise data analysis methods, in conjunction with artificial intelligence and machine learning technologies, is crucial for addressing this issue in the future. Lastly, insufficient integration of omics technologies with TCM theory: Future research should focus on how to deeply integrate omics strategies with “TCM theory” and “TCM metabolite compatibility” studies, using modern scientific data to interpret the theories and methods of TCM in preventing and treating MBDs. This will help promote the modernization and internationalization of TCM, providing more comprehensive solutions for the prevention and treatment of MBDs.

In summary, omics technologies have provided powerful tools for the research and prevention of MBDs, but existing challenges must be overcome. In the future, by expanding the scope of research, strengthening multi-omics integration, enhancing data analysis capabilities, and deepening the integration of omics technologies with TCM theory, we will further advance the in-depth development of MBD research and lay a solid foundation for developing more effective prevention and treatment strategies.

## Abbreviations

BMC, bone mineral content; BMD, bone mineral density; DOP, diabetic osteoporosis; FGF23, Fibroblast growth factor 23; GIOP, Glucocorticoid-induced osteoporosis; LC-MS, liquid chromatography-mass spectrometry; MBD, Metabolic bone disease; MSCs, mesenchymal stem cells; OP, Osteoporosis; OVX, Ovariectomized; PMOP, Postmenopausal osteoporosis; TCA, tricarboxylic acid; TCM, traditional Chinese medicine; UPLC/Q-TOF-MS, ultraperformance liquid chromatography/quadrupole time-of-flight.
