# Supplementary material for: Advances in omics technologies for traditional Chinese medicine in the prevention and treatment of metabolic bone diseases
Source: Front Pharmacol. 2025 Apr 11;16:1576286. doi: 10.3389/fphar.2025.1576286 (PMC12021879; doi:10.3389/fphar.2025.1576286)
Supplement: Supplementary file 1 [file Table1.docx]

*Supplementary Material*

Advances in Omics Technologies for Traditional Chinese Medicine in the Prevention and Treatment of Metabolic Bone Diseases

Author: Wenjun Cai^1,2 *^, Lili Jiang^3*^, Changwei Zhao^1,4 †^, Xiaoling Zhou^5 †^

† Corresponding author

Changwei Zhao, Email address: zcw_1980@126.com.

Xiaoling Zhou, Email address: zxl19800110@163.com

Supplementary Table 1: Active Ingredients of Botanical Drugs

| **Chinese Name** | **Latin Name** | **Family** | **Species** | **Part Used** | **Molecule ID** | **Molecule** |
| --- | --- | --- | --- | --- | --- | --- |
| Dihuang | Rehmannia glutinosa (Gaertn.) Libosch. ex DC. | [Orobanchaceae](https://powo.science.kew.org/taxon/urn:lsid:ipni.org:names:30003446-2) | Rehmannia glutinosa | Root | MOL000359 | [sitosterol](https://www.tcmsp-e.com/molecule.php?qn=359) |
|  |  |  |  |  | MOL000449 | [Stigmasterol](https://www.tcmsp-e.com/molecule.php?qn=449) |
| Xiyangshen | Panax quinquefolius L. | Araliaceae | Panax quinquefolius | Root | MOL011435 | PQ-2 |
|  |  |  |  |  | MOL011442 | (8S,9S,10R,13R,14S,17R)-17-[(1R,4R)-4-ethyl-1,5-dimethylhexyl]-10,13-dimethyl-1,2,8,9,11,12,14,15,16,17-decahydrocyclopenta[a]phenanthren-7-one |
|  |  |  |  |  | MOL000358 | beta-sitosterol |
|  |  |  |  |  | MOL006774 | stigmast-7-enol |
|  |  |  |  |  | MOL006980 | papaverine |
|  |  |  |  |  | MOL008173 | daucosterol_qt |
|  |  |  |  |  | MOL008397 | Daturilin |
| Niuxi | Achyranthes bidentata Blume | Amaranthaceae | Achyranthes bidentata | Root | MOL012505 | bidentatoside,ii_qt |
|  |  |  |  |  | MOL000085 | beta-daucosterol_qt |
|  |  |  |  |  | MOL002643 | delta 7-stigmastenol |
|  |  |  |  |  | MOL004355 | Spinasterol |
|  |  |  |  |  | MOL000358 | beta-sitosterol |
|  |  |  |  |  | MOL001006 | poriferasta-7,22E-dien-3beta-ol |
|  |  |  |  |  | MOL000449 | Stigmasterol |
|  |  |  |  |  | MOL002897 | epiberberine |
|  |  |  |  |  | MOL001454 | berberine |
|  |  |  |  |  | MOL001458 | coptisine |
|  |  |  |  |  | MOL000098 | quercetin |
|  |  |  |  |  | MOL000422 | kaempferol |
|  |  |  |  |  | MOL003847 | Inophyllum E |
|  |  |  |  |  | MOL002714 | baicalein |
|  |  |  |  |  | MOL000173 | wogonin |
| Lancitou | Echinops davuricus Fisch. ex Hornem. | Asteraceae | Echinops davuricus | Root | MOL002307 | 20-Hexadecanoylingenol |
|  |  |  |  |  | MOL011455 | 20-Hexadecanoylingenol |
|  |  |  |  |  | MOL000932 | alpha-Farnesene |
|  |  |  |  |  | MOL000024 | alpha-humulene |
|  |  |  |  |  | MOL008058 | alpha-humulene |
|  |  |  |  |  | MOL001203 | α-santalene |
|  |  |  |  |  | MOL007551 | α-santalene |
|  |  |  |  |  | MOL000029 | β-humulene |
|  |  |  |  |  | MOL002451 | β-humulene |
|  |  |  |  |  | MOL000358 | beta-sitosterol |
|  |  |  |  |  | MOL001986 | beta-sitosterol |
|  |  |  |  |  | MOL001987 | beta-sitosterol |
|  |  |  |  |  | MOL008583 | beta-sitosterol |
|  |  |  |  |  | MOL000093 | daucosterol |
|  |  |  |  |  | MOL000515 | Melissic acid |
|  |  |  |  |  | MOL000983 | n-Triacontanol |
|  |  |  |  |  | MOL001972 | pulegone |
|  |  |  |  |  | MOL004632 | Pulsatillic acid |
| Gusuibu | Drynaria roosii Nakaike | [Polypodiaceae](https://powo.science.kew.org/taxon/urn:lsid:ipni.org:names:30000471-2) | Drynaria roosii | Rhizome | MOL009087 | marioside_qt |
|  |  |  |  |  | MOL004328 | naringenin |
|  |  |  |  |  | MOL001040 | (2R)-5,7-dihydroxy-2-(4-hydroxyphenyl)chroman-4-one |
|  |  |  |  |  | MOL000422 | kaempferol |
|  |  |  |  |  | MOL002914 | Eriodyctiol (flavanone) |
|  |  |  |  |  | MOL005190 | eriodictyol |
|  |  |  |  |  | MOL001978 | Aureusidin |
|  |  |  |  |  | MOL000006 | luteolin |
|  |  |  |  |  | MOL009091 | xanthogalenol |
|  |  |  |  |  | MOL000358 | beta-sitosterol |
|  |  |  |  |  | MOL000449 | Stigmasterol |
|  |  |  |  |  | MOL009061 | 22-Stigmasten-3-one |
|  |  |  |  |  | MOL009075 | cycloartenone |
|  |  |  |  |  | MOL009063 | Cyclolaudenol acetate |
|  |  |  |  |  | MOL009076 | cyclolaudenol |
| Duzhong | Eucommia ulmoides Oliv. | Eucommiaceae | Eucommia ulmoides | Bark | MOL000211 | [Mairin](https://www.tcmsp-e.com/molecule.php?qn=211) |
|  |  |  |  |  | MOL000358 | [beta-sitosterol](https://www.tcmsp-e.com/molecule.php?qn=358) |
|  |  |  |  |  | MOL000422 | [kaempferol](https://www.tcmsp-e.com/molecule.php?qn=422) |
|  |  |  |  |  | MOL000443 | [Erythraline](https://www.tcmsp-e.com/molecule.php?qn=443) |
|  |  |  |  |  | MOL007059 | [3-beta-Hydroxymethyllenetanshiquinone](https://www.tcmsp-e.com/molecule.php?qn=7059) |
|  |  |  |  |  | MOL009015 | [(-)-Tabernemontanine](https://www.tcmsp-e.com/molecule.php?qn=9015) |
|  |  |  |  |  | MOL009027 | [Cyclopamine](https://www.tcmsp-e.com/molecule.php?qn=9027) |
|  |  |  |  |  | MOL009029 | [Dehydrodiconiferyl alcohol 4,gamma'-di-O-beta-D-glucopyanoside_qt](https://www.tcmsp-e.com/molecule.php?qn=9029) |
|  |  |  |  |  | MOL009042 | [Helenalin](https://www.tcmsp-e.com/molecule.php?qn=9042) |
|  |  |  |  |  | MOL009053 | [4-[(2S,3R)-5-[(E)-3-hydroxyprop-1-enyl]-7-methoxy-3-methylol-2,3-dihydrobenzofuran-2-yl]-2-methoxy-phenol](https://www.tcmsp-e.com/molecule.php?qn=9053) |
|  |  |  |  |  | MOL000098 | [quercetin](https://www.tcmsp-e.com/molecule.php?qn=98) |
|  |  |  |  |  | MOL002773 | [beta-carotene](https://www.tcmsp-e.com/molecule.php?qn=2773) |
|  |  |  |  |  | MOL008240 | [(E)-3-[4-[(1R,2R)-2-hydroxy-2-(4-hydroxy-3-methoxy-phenyl)-1-methylol-ethoxy]-3-methoxy-phenyl]acrolein](https://www.tcmsp-e.com/molecule.php?qn=8240) |
|  |  |  |  |  | MOL011604 | [Syringetin](https://www.tcmsp-e.com/molecule.php?qn=11604) |
| Xuduan | Dipsacus asper Wall. ex DC. | Caprifoliaceae | Dipsacus asper | Root | MOL003152 | Gentisin |
|  |  |  |  |  | MOL000358 | beta-sitosterol |
|  |  |  |  |  | MOL000359 | sitosterol |
|  |  |  |  |  | MOL008188 | Japonine |
|  |  |  |  |  | MOL009323 | Sylvestroside III_qt |
| Roucongrong | Cistanche deserticola Ma | Orobanchaceae | Cistanche deserticola | Stem | MOL000358 | [beta-sitosterol](https://www.tcmsp-e.com/molecule.php?qn=358) |
|  |  |  |  |  | MOL005320 | [arachidonate](https://www.tcmsp-e.com/molecule.php?qn=5320) |
|  |  |  |  |  | MOL005384 | [suchilactone](https://www.tcmsp-e.com/molecule.php?qn=5384) |
|  |  |  |  |  | MOL000098 | [quercetin](https://www.tcmsp-e.com/molecule.php?qn=98) |
|  |  |  |  |  | MOL008871 | [Marckine](https://www.tcmsp-e.com/molecule.php?qn=8871) |
| Bajitian | Gynochthodes officinalis (F.C.How) Razafim. & B.Bremer | Rubiaceae | Gynochthodes officinalis | Root | MOL002883 | [Ethyl oleate (NF)](https://www.tcmsp-e.com/molecule.php?qn=2883) |
|  |  |  |  |  | MOL000358 | [beta-sitosterol](https://www.tcmsp-e.com/molecule.php?qn=358) |
|  |  |  |  |  | MOL000359 | [sitosterol](https://www.tcmsp-e.com/molecule.php?qn=359) |
|  |  |  |  |  | MOL006147 | [Alizarin-2-methylether](https://www.tcmsp-e.com/molecule.php?qn=6147) |
|  |  |  |  |  | MOL009495 | [2-hydroxy-1,5-dimethoxy-6-(methoxymethyl)-9,10-anthraquinone](https://www.tcmsp-e.com/molecule.php?qn=9495) |
|  |  |  |  |  | MOL009496 | [1,5,7-trihydroxy-6-methoxy-2-methoxymethylanthracenequinone](https://www.tcmsp-e.com/molecule.php?qn=9496) |
|  |  |  |  |  | MOL009500 | [1,6-dihydroxy-5-methoxy-2-(methoxymethyl)-9,10-anthraquinone](https://www.tcmsp-e.com/molecule.php?qn=9500) |
|  |  |  |  |  | MOL009503 | [1-hydroxy-3-methoxy-9,10-anthraquinone](https://www.tcmsp-e.com/molecule.php?qn=9503) |
|  |  |  |  |  | MOL009504 | [1-hydroxy-6-hydroxymethylanthracenequinone](https://www.tcmsp-e.com/molecule.php?qn=9504) |
|  |  |  |  |  | MOL009513 | [2-hydroxy-1,8-dimethoxy-7-methoxymethylanthracenequinone](https://www.tcmsp-e.com/molecule.php?qn=9513) |
|  |  |  |  |  | MOL009519 | [(2R,3S)-(+)-3',5-Dihydroxy-4 ,7-dimethoxydihydroflavonol](https://www.tcmsp-e.com/molecule.php?qn=9519) |
|  |  |  |  |  | MOL009524 | [3beta,20(R),5-alkenyl-stigmastol](https://www.tcmsp-e.com/molecule.php?qn=9524) |
|  |  |  |  |  | MOL009525 | [3beta-24S(R)-butyl-5-alkenyl-cholestol](https://www.tcmsp-e.com/molecule.php?qn=9525) |
|  |  |  |  |  | MOL009562 | [Ohioensin-A](https://www.tcmsp-e.com/molecule.php?qn=9562) |
| Douchi | Glycine max (L.) Merr. | Fabaceae | Glycine max | Seed | MOL008400 | glycitein |
| Buguzhi | Cullen corylifolium (L.) Medik. | Fabaceae | Cullen corylifolium | Dried ripe fruit | MOL003590 | angelicin |
|  |  |  |  |  | MOL005009 | Corylifolinin |
|  |  |  |  |  | MOL000093 | daucosterol |
|  |  |  |  |  | MOL001525 | daucosterol |
|  |  |  |  |  | MOL002296 | daucosterol |
|  |  |  |  |  | MOL005868 | daucosterol |
|  |  |  |  |  | MOL007096 | daucosterol |
|  |  |  |  |  | MOL008172 | daucosterol |
|  |  |  |  |  | MOL008926 | daucosterol |
|  |  |  |  |  | MOL010584 | daucosterol |
|  |  |  |  |  | MOL012237 | daucosterol |
|  |  |  |  |  | MOL000448 | isobavachin |
|  |  |  |  |  | MOL001393 | myristic acid |
|  |  |  |  |  | MOL005639 | Neobavachalcone |
|  |  |  |  |  | MOL001950 | psoralen |
|  |  |  |  |  | MOL000860 | stearic acid |
|  |  |  |  |  | MOL000449 | stigmasterol |
|  |  |  |  |  | MOL002045 | stigmasterol |
|  |  |  |  |  | MOL008159 | TRIACONTANE |

Supplementary Table 2: Composition and Active Ingredients of the Chinese herbal formula

| **Chinese herbal formula** | **Chinese Name** | **English/Latin Name** | **Family** | **Species** | **Part Used** | **Molecule ID** | **Molecule** |
| --- | --- | --- | --- | --- | --- | --- | --- |
| Gushukang capsules | Yinyanghuo | Epimedium brevicornu Maxim. | [Berberidaceae](https://powo.science.kew.org/taxon/urn:lsid:ipni.org:names:30000989-2) | Epimedium brevicornu | Leaves | MOL000098 | quercetin |
|  |  |  |  |  |  | MOL000006 | luteolin |
|  |  |  |  |  |  | MOL000422 | kaempferol |
|  |  |  |  |  |  | MOL004382 | Yinyanghuo A |
|  |  |  |  |  |  | MOL003044 | Chryseriol |
|  |  |  |  |  |  | MOL001792 | DFV |
|  |  |  |  |  |  | MOL004386 | Yinyanghuo E |
|  |  |  |  |  |  | MOL003542 | 8-Isopentenyl-kaempferol |
|  |  |  |  |  |  | MOL004373 | Anhydroicaritin |
|  |  |  |  |  |  | MOL004384 | Yinyanghuo C |
|  |  |  |  |  |  | MOL004380 | C-Homoerythrinan, 1,6-didehydro-3,15,16-trimethoxy-, (3.beta.)- |
|  |  |  |  |  |  | MOL000359 | sitosterol |
|  |  |  |  |  |  | MOL001645 | Linoleyl acetate |
|  |  |  |  |  |  | MOL001510 | 24-epicampesterol |
|  |  |  |  |  |  | MOL001771 | poriferast-5-en-3beta-ol |
|  |  |  |  |  |  | MOL004391 | 8-(3-methylbut-2-enyl)-2-phenyl-chromone |
|  | Dihuang | Rehmannia glutinosa (Gaertn.) Libosch. ex DC. | [Orobanchaceae](https://powo.science.kew.org/taxon/urn:lsid:ipni.org:names:30003446-2) | Rehmannia glutinosa | Root | MOL000359 | [sitosterol](https://www.tcmsp-e.com/molecule.php?qn=359) |
|  |  |  |  |  |  | MOL000449 | [Stigmasterol](https://www.tcmsp-e.com/molecule.php?qn=449) |
|  | Gusuibu | Drynaria roosii Nakaike | [Polypodiaceae](https://powo.science.kew.org/taxon/urn:lsid:ipni.org:names:30000471-2) | Drynaria roosii | Rhizome | MOL009087 | marioside_qt |
|  |  |  |  |  |  | MOL004328 | naringenin |
|  |  |  |  |  |  | MOL001040 | (2R)-5,7-dihydroxy-2-(4-hydroxyphenyl)chroman-4-one |
|  |  |  |  |  |  | MOL000422 | kaempferol |
|  |  |  |  |  |  | MOL002914 | Eriodyctiol (flavanone) |
|  |  |  |  |  |  | MOL005190 | eriodictyol |
|  |  |  |  |  |  | MOL001978 | Aureusidin |
|  |  |  |  |  |  | MOL000006 | luteolin |
|  |  |  |  |  |  | MOL009091 | xanthogalenol |
|  |  |  |  |  |  | MOL000358 | beta-sitosterol |
|  |  |  |  |  |  | MOL000449 | Stigmasterol |
|  |  |  |  |  |  | MOL009061 | 22-Stigmasten-3-one |
|  |  |  |  |  |  | MOL009075 | cycloartenone |
|  |  |  |  |  |  | MOL009063 | Cyclolaudenol acetate |
|  |  |  |  |  |  | MOL009076 | cyclolaudenol |
|  | Huangqi | [Astragalus mongholicus Bunge](https://mpns.science.kew.org/mpns-portal/plantDetail?plantId=2661222&query=Astragalus+membranaceus+%28Fisch.%29Bge.&filter=&fuzzy=false&nameType=all&dbs=wcs) | [Fabaceae](https://powo.science.kew.org/taxon/urn:lsid:ipni.org:names:30000147-2) | Astragalus mongholicus | Root | MOL000211 | Mairin |
|  |  |  |  |  |  | MOL000239 | Jaranol |
|  |  |  |  |  |  | MOL000296 | hederagenin |
|  |  |  |  |  |  | MOL000033 | (3S,8S,9S,10R,13R,14S,17R)-10,13-dimethyl-17-[(2R,5S)-5-propan-2-yloctan-2-yl]-2,3,4,7,8,9,11,12,14,15,16,17-dodecahydro-1H-cyclopenta[a]phenanthren-3-ol |
|  |  |  |  |  |  | MOL000354 | isorhamnetin |
|  |  |  |  |  |  | MOL000371 | 3,9-di-O-methylnissolin |
|  |  |  |  |  |  | MOL000380 | (6aR,11aR)-9,10-dimethoxy-6a,11a-dihydro-6H-benzofurano[3,2-c]chromen-3-ol |
|  |  |  |  |  |  | MOL000387 | Bifendate |
|  |  |  |  |  |  | MOL000392 | formononetin |
|  |  |  |  |  |  | MOL000398 | isoflavanone |
|  |  |  |  |  |  | MOL000417 | Calycosin |
|  |  |  |  |  |  | MOL000422 | kaempferol |
|  |  |  |  |  |  | MOL000442 | 1,7-Dihydroxy-3,9-dimethoxy pterocarpene |
|  |  |  |  |  |  | MOL000098 | quercetin |
|  | Danshen | Salvia miltiorrhiza Bunge | [Lamiaceae](https://powo.science.kew.org/taxon/urn:lsid:ipni.org:names:30000097-2) | Salvia miltiorrhiza | Root | MOL001601 | 1,2,5,6-tetrahydrotanshinone |
|  |  |  |  |  |  | MOL001659 | Poriferasterol |
|  |  |  |  |  |  | MOL001771 | poriferast-5-en-3beta-ol |
|  |  |  |  |  |  | MOL002222 | sugiol |
|  |  |  |  |  |  | MOL002651 | Dehydrotanshinone II A |
|  |  |  |  |  |  | MOL000006 | luteolin |
|  |  |  |  |  |  | MOL007036 | 5,6-dihydroxy-7-isopropyl-1,1-dimethyl-2,3-dihydrophenanthren-4-one |
|  |  |  |  |  |  | MOL007041 | 2-isopropyl-8-methylphenanthrene-3,4-dione |
|  |  |  |  |  |  | MOL007045 | 3α-hydroxytanshinoneⅡa |
|  |  |  |  |  |  | MOL007048 | (E)-3-[2-(3,4-dihydroxyphenyl)-7-hydroxy-benzofuran-4-yl]acrylic acid |
|  |  |  |  |  |  | MOL007049 | 4-methylenemiltirone |
|  |  |  |  |  |  | MOL007050 | 2-(4-hydroxy-3-methoxyphenyl)-5-(3-hydroxypropyl)-7-methoxy-3-benzofurancarboxaldehyde |
|  |  |  |  |  |  | MOL007058 | formyltanshinone |
|  |  |  |  |  |  | MOL007059 | 3-beta-Hydroxymethyllenetanshiquinone |
|  |  |  |  |  |  | MOL007061 | Methylenetanshinquinone |
|  |  |  |  |  |  | MOL007068 | Przewaquinone B |
|  |  |  |  |  |  | MOL007069 | przewaquinone c |
|  |  |  |  |  |  | MOL007070 | (6S,7R)-6,7-dihydroxy-1,6-dimethyl-8,9-dihydro-7H-naphtho[8,7-g]benzofuran-10,11-dione |
|  |  |  |  |  |  | MOL007071 | przewaquinone f |
|  |  |  |  |  |  | MOL007077 | sclareol |
|  |  |  |  |  |  | MOL007079 | tanshinaldehyde |
|  |  |  |  |  |  | MOL007081 | Danshenol B |
|  |  |  |  |  |  | MOL007082 | Danshenol A |
|  |  |  |  |  |  | MOL007085 | Salvilenone |
|  |  |  |  |  |  | MOL007088 | cryptotanshinone |
|  |  |  |  |  |  | MOL007093 | dan-shexinkum d |
|  |  |  |  |  |  | MOL007094 | danshenspiroketallactone |
|  |  |  |  |  |  | MOL007098 | deoxyneocryptotanshinone |
|  |  |  |  |  |  | MOL007100 | dihydrotanshinlactone |
|  |  |  |  |  |  | MOL007101 | dihydrotanshinoneⅠ |
|  |  |  |  |  |  | MOL007108 | isocryptotanshi-none |
|  |  |  |  |  |  | MOL007111 | Isotanshinone II |
|  |  |  |  |  |  | MOL007115 | manool |
|  |  |  |  |  |  | MOL007118 | microstegiol |
|  |  |  |  |  |  | MOL007119 | miltionone Ⅰ |
|  |  |  |  |  |  | MOL007122 | Miltirone |
|  |  |  |  |  |  | MOL007124 | neocryptotanshinone ii |
|  |  |  |  |  |  | MOL007125 | neocryptotanshinone |
|  |  |  |  |  |  | MOL007127 | 1-methyl-8,9-dihydro-7H-naphtho[5,6-g]benzofuran-6,10,11-trione |
|  |  |  |  |  |  | MOL007130 | prolithospermic acid |
|  |  |  |  |  |  | MOL007140 | (Z)-3-[2-[(E)-2-(3,4-dihydroxyphenyl)vinyl]-3,4-dihydroxy-phenyl]acrylic acid |
|  |  |  |  |  |  | MOL007149 | NSC 122421 |
|  |  |  |  |  |  | MOL007150 | (6S)-6-hydroxy-1-methyl-6-methylol-8,9-dihydro-7H-naphtho[8,7-g]benzofuran-10,11-quinone |
|  |  |  |  |  |  | MOL007151 | Tanshindiol B |
|  |  |  |  |  |  | MOL007152 | Przewaquinone E |
|  |  |  |  |  |  | MOL007154 | tanshinone iia |
|  |  |  |  |  |  | MOL007155 | (6S)-6-(hydroxymethyl)-1,6-dimethyl-8,9-dihydro-7H-naphtho[8,7-g]benzofuran-10,11-dione |
|  |  |  |  |  |  | MOL007156 | tanshinone Ⅵ |
| Zishen Jiangtang Pill | Huangqi | [Astragalus mongholicus Bunge](https://mpns.science.kew.org/mpns-portal/plantDetail?plantId=2661222&query=Astragalus+membranaceus+%28Fisch.%29Bge.&filter=&fuzzy=false&nameType=all&dbs=wcs) | [Fabaceae](https://powo.science.kew.org/taxon/urn:lsid:ipni.org:names:30000147-2) | Astragalus mongholicus | Root | MOL000211 | Mairin |
|  |  |  |  |  |  | MOL000239 | Jaranol |
|  |  |  |  |  |  | MOL000296 | hederagenin |
|  |  |  |  |  |  | MOL000033 | (3S,8S,9S,10R,13R,14S,17R)-10,13-dimethyl-17-[(2R,5S)-5-propan-2-yloctan-2-yl]-2,3,4,7,8,9,11,12,14,15,16,17-dodecahydro-1H-cyclopenta[a]phenanthren-3-ol |
|  |  |  |  |  |  | MOL000354 | isorhamnetin |
|  |  |  |  |  |  | MOL000371 | 3,9-di-O-methylnissolin |
|  |  |  |  |  |  | MOL000380 | (6aR,11aR)-9,10-dimethoxy-6a,11a-dihydro-6H-benzofurano[3,2-c]chromen-3-ol |
|  |  |  |  |  |  | MOL000387 | Bifendate |
|  |  |  |  |  |  | MOL000392 | formononetin |
|  |  |  |  |  |  | MOL000398 | isoflavanone |
|  |  |  |  |  |  | MOL000417 | Calycosin |
|  |  |  |  |  |  | MOL000422 | kaempferol |
|  |  |  |  |  |  | MOL000442 | 1,7-Dihydroxy-3,9-dimethoxy pterocarpene |
|  |  |  |  |  |  | MOL000098 | quercetin |
|  | Dihuang | Rehmannia glutinosa (Gaertn.) Libosch. ex DC. | [Orobanchaceae](https://powo.science.kew.org/taxon/urn:lsid:ipni.org:names:30003446-2) | Rehmannia glutinosa | Root | MOL000359 | [sitosterol](https://www.tcmsp-e.com/molecule.php?qn=359) |
|  |  |  |  |  |  | MOL000449 | [Stigmasterol](https://www.tcmsp-e.com/molecule.php?qn=449) |
|  | Wuweizi | Schisandra chinensis (Turcz.) Baill. | Schisandraceae | Schisandra chinensis | Dried ripe fruit | MOL008968 | Gomisin-A |
|  |  |  |  |  |  | MOL008957 | Schizandrer B |
|  |  |  |  |  |  | MOL008956 | Angeloylgomisin O |
|  |  |  |  |  |  | MOL008974 | Gomisin G |
|  |  |  |  |  |  | MOL008978 | Gomisin R |
|  |  |  |  |  |  | MOL005317 | Deoxyharringtonine |
|  |  |  |  |  |  | MOL008992 | Wuweizisu C |
|  | Yinyanghuo | Epimedium brevicornu Maxim. | [Berberidaceae](https://powo.science.kew.org/taxon/urn:lsid:ipni.org:names:30000989-2) | Epimedium brevicornu | Leaves | MOL000098 | quercetin |
|  |  |  |  |  |  | MOL000006 | luteolin |
|  |  |  |  |  |  | MOL000422 | kaempferol |
|  |  |  |  |  |  | MOL004382 | Yinyanghuo A |
|  |  |  |  |  |  | MOL003044 | Chryseriol |
|  |  |  |  |  |  | MOL001792 | DFV |
|  |  |  |  |  |  | MOL004386 | Yinyanghuo E |
|  |  |  |  |  |  | MOL003542 | 8-Isopentenyl-kaempferol |
|  |  |  |  |  |  | MOL004373 | Anhydroicaritin |
|  |  |  |  |  |  | MOL004384 | Yinyanghuo C |
|  |  |  |  |  |  | MOL004380 | C-Homoerythrinan, 1,6-didehydro-3,15,16-trimethoxy-, (3.beta.)- |
|  |  |  |  |  |  | MOL000359 | sitosterol |
|  |  |  |  |  |  | MOL001645 | Linoleyl acetate |
|  |  |  |  |  |  | MOL001510 | 24-epicampesterol |
|  |  |  |  |  |  | MOL001771 | poriferast-5-en-3beta-ol |
|  |  |  |  |  |  | MOL004391 | 8-(3-methylbut-2-enyl)-2-phenyl-chromone |
|  | Gouji | Cibotium barometz （L.） J.Sm. | Cyatheaceae | Cibotium barometz | Rhizome | MOL003389 | 3'-O-Methylorobol |
|  |  |  |  |  |  | MOL002608 | Aspidinol |
|  |  |  |  |  |  | MOL000448 | isobavachin |
|  |  |  |  |  |  | MOL000422 | kaempferol |
|  |  |  |  |  |  | MOL004328 | naringenin |
|  |  |  |  |  |  | MOL003673 | Wighteone |
|  | Xianmao | Curculigo orchioides Gaertn. | [Hypoxidaceae](https://powo.science.kew.org/taxon/urn:lsid:ipni.org:names:77126735-1) | Curculigo orchioides | Root | MOL003578 | [Cycloartenol](https://www.tcmsp-e.com/molecule.php?qn=3578) |
|  |  |  |  |  |  | MOL000358 | [beta-sitosterol](https://www.tcmsp-e.com/molecule.php?qn=358) |
|  |  |  |  |  |  | MOL004114 | [3,2',4',6'-Tetrahydroxy-4,3'-dimethoxy chalcone](https://www.tcmsp-e.com/molecule.php?qn=4114) |
|  |  |  |  |  |  | MOL004125 | [Curculigoside B_qt](https://www.tcmsp-e.com/molecule.php?qn=4125) |
|  |  |  |  |  |  | MOL000449 | [Stigmasterol](https://www.tcmsp-e.com/molecule.php?qn=449) |
|  | Niuxi | Achyranthes bidentata Blume | Amaranthaceae | Achyranthes bidentata | Root | MOL012505 | bidentatoside,ii_qt |
|  |  |  |  |  |  | MOL000085 | beta-daucosterol_qt |
|  |  |  |  |  |  | MOL002643 | delta 7-stigmastenol |
|  |  |  |  |  |  | MOL004355 | Spinasterol |
|  |  |  |  |  |  | MOL000358 | beta-sitosterol |
|  |  |  |  |  |  | MOL001006 | poriferasta-7,22E-dien-3beta-ol |
|  |  |  |  |  |  | MOL000449 | Stigmasterol |
|  |  |  |  |  |  | MOL002897 | epiberberine |
|  |  |  |  |  |  | MOL001454 | berberine |
|  |  |  |  |  |  | MOL001458 | coptisine |
|  |  |  |  |  |  | MOL000098 | quercetin |
|  |  |  |  |  |  | MOL000422 | kaempferol |
|  |  |  |  |  |  | MOL003847 | Inophyllum E |
|  |  |  |  |  |  | MOL002714 | baicalein |
|  |  |  |  |  |  | MOL000173 | wogonin |
|  | Dangshen | Codonopsis pilosula (Franch.) Nannf. | [Campanulaceae](https://powo.science.kew.org/taxon/urn:lsid:ipni.org:names:30000171-2) | Codonopsis pilosula | Root | MOL001006 | [poriferasta-7,22E-dien-3beta-ol](https://www.tcmsp-e.com/molecule.php?qn=1006) |
|  |  |  |  |  |  | MOL002140 | [Perlolyrine](https://www.tcmsp-e.com/molecule.php?qn=2140) |
|  |  |  |  |  |  | MOL003036 | [ZINC03978781](https://www.tcmsp-e.com/molecule.php?qn=3036) |
|  |  |  |  |  |  | MOL000449 | [Stigmasterol](https://www.tcmsp-e.com/molecule.php?qn=449) |
|  |  |  |  |  |  | MOL003896 | [7-Methoxy-2-methyl isoflavone](https://www.tcmsp-e.com/molecule.php?qn=3896) |
|  |  |  |  |  |  | MOL004355 | [Spinasterol](https://www.tcmsp-e.com/molecule.php?qn=4355) |
|  |  |  |  |  |  | MOL004492 | [Chrysanthemaxanthin](https://www.tcmsp-e.com/molecule.php?qn=4492) |
|  |  |  |  |  |  | MOL005321 | [Frutinone A](https://www.tcmsp-e.com/molecule.php?qn=5321) |
|  |  |  |  |  |  | MOL000006 | [luteolin](https://www.tcmsp-e.com/molecule.php?qn=6) |
|  |  |  |  |  |  | MOL006774 | [stigmast-7-enol](https://www.tcmsp-e.com/molecule.php?qn=6774) |
|  |  |  |  |  |  | MOL007059 | [3-beta-Hydroxymethyllenetanshiquinone](https://www.tcmsp-e.com/molecule.php?qn=7059) |
|  |  |  |  |  |  | MOL007514 | [methyl icosa-11,14-dienoate](https://www.tcmsp-e.com/molecule.php?qn=7514) |
|  |  |  |  |  |  | MOL008391 | [5alpha-Stigmastan-3,6-dione](https://www.tcmsp-e.com/molecule.php?qn=8391) |
|  |  |  |  |  |  | MOL008397 | [Daturilin](https://www.tcmsp-e.com/molecule.php?qn=8397) |
|  |  |  |  |  |  | MOL008400 | [glycitein](https://www.tcmsp-e.com/molecule.php?qn=8400) |
|  |  |  |  |  |  | MOL008407 | [(8S,9S,10R,13R,14S,17R)-17-[(E,2R,5S)-5-ethyl-6-methylhept-3-en-2-yl]-10,13-dimethyl-1,2,4,7,8,9,11,12,14,15,16,17-dodecahydrocyclopenta[a]phenanthren-3-one](https://www.tcmsp-e.com/molecule.php?qn=8407) |
|  |  |  |  |  |  | MOL008411 | [11-Hydroxyrankinidine](https://www.tcmsp-e.com/molecule.php?qn=8411) |
|  | Gusuibu | Drynaria roosii Nakaike | [Polypodiaceae](https://powo.science.kew.org/taxon/urn:lsid:ipni.org:names:30000471-2) | Drynaria roosii | Rhizome | MOL009087 | marioside_qt |
|  |  |  |  |  |  | MOL004328 | naringenin |
|  |  |  |  |  |  | MOL001040 | (2R)-5,7-dihydroxy-2-(4-hydroxyphenyl)chroman-4-one |
|  |  |  |  |  |  | MOL000422 | kaempferol |
|  |  |  |  |  |  | MOL002914 | Eriodyctiol (flavanone) |
|  |  |  |  |  |  | MOL005190 | eriodictyol |
|  |  |  |  |  |  | MOL001978 | Aureusidin |
|  |  |  |  |  |  | MOL000006 | luteolin |
|  |  |  |  |  |  | MOL009091 | xanthogalenol |
|  |  |  |  |  |  | MOL000358 | beta-sitosterol |
|  |  |  |  |  |  | MOL000449 | Stigmasterol |
|  |  |  |  |  |  | MOL009061 | 22-Stigmasten-3-one |
|  |  |  |  |  |  | MOL009075 | cycloartenone |
|  |  |  |  |  |  | MOL009063 | Cyclolaudenol acetate |
|  |  |  |  |  |  | MOL009076 | cyclolaudenol |
|  | Sanqi | Panax notoginseng (Burkill) F.H.Chen | [Araliaceae](https://powo.science.kew.org/taxon/urn:lsid:ipni.org:names:30001539-2) | Panax notoginseng | Root and Rhizome | MOL001494 | [Mandenol](https://www.tcmsp-e.com/molecule.php?qn=1494) |
|  |  |  |  |  |  | MOL001792 | [DFV](https://www.tcmsp-e.com/molecule.php?qn=1792) |
|  |  |  |  |  |  | MOL000358 | [beta-sitosterol](https://www.tcmsp-e.com/molecule.php?qn=358) |
|  |  |  |  |  |  | MOL000449 | [Stigmasterol](https://www.tcmsp-e.com/molecule.php?qn=449) |
|  |  |  |  |  |  | MOL000098 | [quercetin](https://www.tcmsp-e.com/molecule.php?qn=98) |
|  | Huangjing | Polygonatum sibiricum Redouté | Asparagaceae | Polygonatum sibiricum | Rhizome | MOL001792 | [DFV](https://www.tcmsp-e.com/molecule.php?qn=1792) |
|  |  |  |  |  |  | MOL002714 | [baicalein](https://www.tcmsp-e.com/molecule.php?qn=2714) |
|  |  |  |  |  |  | MOL002959 | [3'-Methoxydaidzein](https://www.tcmsp-e.com/molecule.php?qn=2959) |
|  |  |  |  |  |  | MOL000358 | [beta-sitosterol](https://www.tcmsp-e.com/molecule.php?qn=358) |
|  |  |  |  |  |  | MOL000359 | [sitosterol](https://www.tcmsp-e.com/molecule.php?qn=359) |
|  |  |  |  |  |  | MOL003889 | [methylprotodioscin_qt](https://www.tcmsp-e.com/molecule.php?qn=3889) |
|  |  |  |  |  |  | MOL004941 | [(2R)-7-hydroxy-2-(4-hydroxyphenyl)chroman-4-one](https://www.tcmsp-e.com/molecule.php?qn=4941) |
|  |  |  |  |  |  | MOL000546 | [diosgenin](https://www.tcmsp-e.com/molecule.php?qn=546) |
|  |  |  |  |  |  | MOL006331 | [4',5-Dihydroxyflavone](https://www.tcmsp-e.com/molecule.php?qn=6331) |
|  |  |  |  |  |  | MOL009760 | [sibiricoside A_qt](https://www.tcmsp-e.com/molecule.php?qn=9760) |
|  |  |  |  |  |  | MOL009766 | [zhonghualiaoine 1](https://www.tcmsp-e.com/molecule.php?qn=9766) |
| Shuanghuangyigu Recipe | Duzhong | Eucommia ulmoides Oliv. | Eucommiaceae | Eucommia ulmoides | Bark | MOL000211 | [Mairin](https://www.tcmsp-e.com/molecule.php?qn=211) |
|  |  |  |  |  |  | MOL000358 | [beta-sitosterol](https://www.tcmsp-e.com/molecule.php?qn=358) |
|  |  |  |  |  |  | MOL000422 | [kaempferol](https://www.tcmsp-e.com/molecule.php?qn=422) |
|  |  |  |  |  |  | MOL000443 | [Erythraline](https://www.tcmsp-e.com/molecule.php?qn=443) |
|  |  |  |  |  |  | MOL007059 | [3-beta-Hydroxymethyllenetanshiquinone](https://www.tcmsp-e.com/molecule.php?qn=7059) |
|  |  |  |  |  |  | MOL009015 | [(-)-Tabernemontanine](https://www.tcmsp-e.com/molecule.php?qn=9015) |
|  |  |  |  |  |  | MOL009027 | [Cyclopamine](https://www.tcmsp-e.com/molecule.php?qn=9027) |
|  |  |  |  |  |  | MOL009029 | [Dehydrodiconiferyl alcohol 4,gamma'-di-O-beta-D-glucopyanoside_qt](https://www.tcmsp-e.com/molecule.php?qn=9029) |
|  |  |  |  |  |  | MOL009042 | [Helenalin](https://www.tcmsp-e.com/molecule.php?qn=9042) |
|  |  |  |  |  |  | MOL009053 | [4-[(2S,3R)-5-[(E)-3-hydroxyprop-1-enyl]-7-methoxy-3-methylol-2,3-dihydrobenzofuran-2-yl]-2-methoxy-phenol](https://www.tcmsp-e.com/molecule.php?qn=9053) |
|  |  |  |  |  |  | MOL000098 | [quercetin](https://www.tcmsp-e.com/molecule.php?qn=98) |
|  |  |  |  |  |  | MOL002773 | [beta-carotene](https://www.tcmsp-e.com/molecule.php?qn=2773) |
|  |  |  |  |  |  | MOL008240 | [(E)-3-[4-[(1R,2R)-2-hydroxy-2-(4-hydroxy-3-methoxy-phenyl)-1-methylol-ethoxy]-3-methoxy-phenyl]acrolein](https://www.tcmsp-e.com/molecule.php?qn=8240) |
|  |  |  |  |  |  | MOL011604 | [Syringetin](https://www.tcmsp-e.com/molecule.php?qn=11604) |
|  | Dihuang | Rehmannia glutinosa (Gaertn.) Libosch. ex DC. | [Orobanchaceae](https://powo.science.kew.org/taxon/urn:lsid:ipni.org:names:30003446-2) | Rehmannia glutinosa | Root | MOL000359 | [sitosterol](https://www.tcmsp-e.com/molecule.php?qn=359) |
|  |  |  |  |  |  | MOL000449 | [Stigmasterol](https://www.tcmsp-e.com/molecule.php?qn=449) |
|  | Huangqi | [Astragalus mongholicus Bunge](https://mpns.science.kew.org/mpns-portal/plantDetail?plantId=2661222&query=Astragalus+membranaceus+%28Fisch.%29Bge.&filter=&fuzzy=false&nameType=all&dbs=wcs) | [Fabaceae](https://powo.science.kew.org/taxon/urn:lsid:ipni.org:names:30000147-2) | Astragalus mongholicus | Root | MOL000211 | Mairin |
|  |  |  |  |  |  | MOL000239 | Jaranol |
|  |  |  |  |  |  | MOL000296 | hederagenin |
|  |  |  |  |  |  | MOL000033 | (3S,8S,9S,10R,13R,14S,17R)-10,13-dimethyl-17-[(2R,5S)-5-propan-2-yloctan-2-yl]-2,3,4,7,8,9,11,12,14,15,16,17-dodecahydro-1H-cyclopenta[a]phenanthren-3-ol |
|  |  |  |  |  |  | MOL000354 | isorhamnetin |
|  |  |  |  |  |  | MOL000371 | 3,9-di-O-methylnissolin |
|  |  |  |  |  |  | MOL000380 | (6aR,11aR)-9,10-dimethoxy-6a,11a-dihydro-6H-benzofurano[3,2-c]chromen-3-ol |
|  |  |  |  |  |  | MOL000387 | Bifendate |
|  |  |  |  |  |  | MOL000392 | formononetin |
|  |  |  |  |  |  | MOL000398 | isoflavanone |
|  |  |  |  |  |  | MOL000417 | Calycosin |
|  |  |  |  |  |  | MOL000422 | kaempferol |
|  |  |  |  |  |  | MOL000442 | 1,7-Dihydroxy-3,9-dimethoxy pterocarpene |
|  |  |  |  |  |  | MOL000098 | quercetin |
|  | Xuduan | Dipsacus asper Wall. ex DC. | Caprifoliaceae | Dipsacus asper | Root | MOL003152 | Gentisin |
|  |  |  |  |  |  | MOL000358 | beta-sitosterol |
|  |  |  |  |  |  | MOL000359 | sitosterol |
|  |  |  |  |  |  | MOL008188 | Japonine |
|  |  |  |  |  |  | MOL009323 | Sylvestroside III_qt |
|  | Gusuibu | Drynaria roosii Nakaike | Polypodiaceae | Drynaria roosii | Rhizome | MOL009087 | marioside_qt |
|  |  |  |  |  |  | MOL004328 | naringenin |
|  |  |  |  |  |  | MOL001040 | (2R)-5,7-dihydroxy-2-(4-hydroxyphenyl)chroman-4-one |
|  |  |  |  |  |  | MOL000422 | kaempferol |
|  |  |  |  |  |  | MOL002914 | Eriodyctiol (flavanone) |
|  |  |  |  |  |  | MOL005190 | eriodictyol |
|  |  |  |  |  |  | MOL001978 | Aureusidin |
|  |  |  |  |  |  | MOL000006 | luteolin |
|  |  |  |  |  |  | MOL009091 | xanthogalenol |
|  |  |  |  |  |  | MOL000358 | beta-sitosterol |
|  |  |  |  |  |  | MOL000449 | Stigmasterol |
|  |  |  |  |  |  | MOL009061 | 22-Stigmasten-3-one |
|  |  |  |  |  |  | MOL009075 | cycloartenone |
|  |  |  |  |  |  | MOL009063 | Cyclolaudenol acetate |
|  |  |  |  |  |  | MOL009076 | cyclolaudenol |
|  | Liujinv | [Siphonostegia chinensis Benth.](https://mpns.science.kew.org/mpns-portal/plantDetail?plantId=2586669&query=Siphonostegiae+Herba++&filter=&fuzzy=false&nameType=all&dbs=wcsCmp) | [Orobanchaceae](https://powo.science.kew.org/taxon/urn:lsid:ipni.org:names:30003446-2) | *Siphonostegia chinensis* | Whole plant | MOL001733 | [EUPATORIN](https://www.tcmsp-e.com/molecule.php?qn=1733) |
|  |  |  |  |  |  | MOL000358 | [beta-sitosterol](https://www.tcmsp-e.com/molecule.php?qn=358) |
|  |  |  |  |  |  | MOL000006 | [luteolin](https://www.tcmsp-e.com/molecule.php?qn=6) |
|  |  |  |  |  |  | MOL008127 | [Ermanin](https://www.tcmsp-e.com/molecule.php?qn=8127) |
|  | Gouqizi | Lycium barbarum L. | Solanaceae | Lycium barbarum | Dried ripe fruit | MOL001323 | Sitosterol alpha1 |
|  |  |  |  |  |  | MOL003578 | Cycloartenol |
|  |  |  |  |  |  | MOL001494 | Mandenol |
|  |  |  |  |  |  | MOL001495 | Ethyl linolenate |
|  |  |  |  |  |  | MOL001979 | LAN |
|  |  |  |  |  |  | MOL000449 | Stigmasterol |
|  |  |  |  |  |  | MOL000358 | beta-sitosterol |
|  |  |  |  |  |  | MOL005438 | campesterol |
|  |  |  |  |  |  | MOL007449 | 24-methylidenelophenol |
|  |  |  |  |  |  | MOL008173 | daucosterol_qt |
|  |  |  |  |  |  | MOL008400 | glycitein |
|  |  |  |  |  |  | MOL000953 | CLR |
|  |  |  |  |  |  | MOL009604 | 14b-pregnane |
|  |  |  |  |  |  | MOL009612 | (24R)-4alpha-Methyl-24-ethylcholesta-7,25-dien-3beta-ylacetate |
|  |  |  |  |  |  | MOL009615 | 24-Methylenecycloartan-3beta,21-diol |
|  |  |  |  |  |  | MOL009617 | 24-ethylcholest-22-enol |
|  |  |  |  |  |  | MOL009618 | 24-ethylcholesta-5,22-dienol |
|  |  |  |  |  |  | MOL009620 | 24-methyl-31-norlanost-9(11)-enol |
|  |  |  |  |  |  | MOL009621 | 24-methylenelanost-8-enol |
|  |  |  |  |  |  | MOL009622 | Fucosterol |
|  |  |  |  |  |  | MOL009631 | 31-Norcyclolaudenol |
|  |  |  |  |  |  | MOL009633 | 31-norlanost-9(11)-enol |
|  |  |  |  |  |  | MOL009634 | 31-norlanosterol |
|  |  |  |  |  |  | MOL009635 | 4,24-methyllophenol |
|  |  |  |  |  |  | MOL009639 | Lophenol |
|  |  |  |  |  |  | MOL009640 | 4alpha,14alpha,24-trimethylcholesta-8,24-dienol |
|  |  |  |  |  |  | MOL009641 | 4alpha,24-dimethylcholesta-7,24-dienol |
|  |  |  |  |  |  | MOL009642 | 4alpha-methyl-24-ethylcholesta-7,24-dienol |
|  |  |  |  |  |  | MOL009644 | 6-Fluoroindole-7-Dehydrocholesterol |
|  |  |  |  |  |  | MOL009646 | 7-O-Methylluteolin-6-C-beta-glucoside_qt |
|  |  |  |  |  |  | MOL009650 | Atropine |
|  |  |  |  |  |  | MOL009651 | Cryptoxanthin monoepoxide |
|  |  |  |  |  |  | MOL009653 | Cycloeucalenol |
|  |  |  |  |  |  | MOL009656 | (E,E)-1-ethyl octadeca-3,13-dienoate |
|  |  |  |  |  |  | MOL009662 | Lantadene A |
|  |  |  |  |  |  | MOL009677 | lanost-8-en-3beta-ol |
|  |  |  |  |  |  | MOL009678 | lanost-8-enol |
|  |  |  |  |  |  | MOL009681 | Obtusifoliol |
|  |  |  |  |  |  | MOL000098 | quercetin |
|  | Shanzhuyu | Cornus officinalis Siebold & Zucc. | Cornaceae | Cornus officinalis | Ripe fruit | MOL002883 | Ethyl oleate (NF) |
|  |  |  |  |  |  | MOL008457 | Tetrahydroalstonine |
|  |  |  |  |  |  | MOL005481 | 2,6,10,14,18-pentamethylicosa-2,6,10,14,18-pentaene |
|  |  |  |  |  |  | MOL005530 | Hydroxygenkwanin |
|  |  |  |  |  |  | MOL000359 | sitosterol |
|  |  |  |  |  |  | MOL001771 | poriferast-5-en-3beta-ol |
|  |  |  |  |  |  | MOL000358 | beta-sitosterol |
|  |  |  |  |  |  | MOL001494 | Mandenol |
|  |  |  |  |  |  | MOL000449 | Stigmasterol |
|  |  |  |  |  |  | MOL005557 | lanosta-8,24-dien-3-ol,3-acetate |
|  |  |  |  |  |  | MOL001495 | Ethyl linolenate |
|  |  |  |  |  |  | MOL005486 | 3,4-Dehydrolycopen-16-al |
|  |  |  |  |  |  | MOL005360 | malkangunin |
|  |  |  |  |  |  | MOL005531 | Telocinobufagin |
|  | fuling | Ophrys apifera Huds. | [Orchidaceae](https://powo.science.kew.org/taxon/urn:lsid:ipni.org:names:30000046-2) | Ophrys apifera | Sclerotium | MOL000291 | Poricoic acid B |
|  |  |  |  |  |  | MOL000290 | Poricoic acid A |
|  |  |  |  |  |  | MOL000273 | (2R)-2-[(3S,5R,10S,13R,14R,16R,17R)-3,16-dihydroxy-4,4,10,13,14-pentamethyl-2,3,5,6,12,15,16,17-octahydro-1H-cyclopenta[a]phenanthren-17-yl]-6-methylhept-5-enoic acid |
|  |  |  |  |  |  | MOL000280 | (2R)-2-[(3S,5R,10S,13R,14R,16R,17R)-3,16-dihydroxy-4,4,10,13,14-pentamethyl-2,3,5,6,12,15,16,17-octahydro-1H-cyclopenta[a]phenanthren-17-yl]-5-isopropyl-hex-5-enoic acid |
|  |  |  |  |  |  | MOL000289 | pachymic acid |
|  |  |  |  |  |  | MOL000276 | 7,9(11)-dehydropachymic acid |
|  |  |  |  |  |  | MOL000296 | hederagenin |
|  |  |  |  |  |  | MOL000279 | Cerevisterol |
|  |  |  |  |  |  | MOL000292 | poricoic acid C |
|  |  |  |  |  |  | MOL000285 | (2R)-2-[(5R,10S,13R,14R,16R,17R)-16-hydroxy-3-keto-4,4,10,13,14-pentamethyl-1,2,5,6,12,15,16,17-octahydrocyclopenta[a]phenanthren-17-yl]-5-isopropyl-hex-5-enoic acid |
|  |  |  |  |  |  | MOL000287 | 3beta-Hydroxy-24-methylene-8-lanostene-21-oic acid |
|  |  |  |  |  |  | MOL000275 | trametenolic acid |
|  |  |  |  |  |  | MOL000282 | ergosta-7,22E-dien-3beta-ol |
|  |  |  |  |  |  | MOL000300 | dehydroeburicoic acid |
|  | Danggui | Angelica sinensis (Oliv.) Diels | Apiaceae | Angelica sinensis | Root | MOL000358 | [beta-sitosterol](https://www.tcmsp-e.com/molecule.php?qn=358) |
|  |  |  |  |  |  | MOL000449 | [Stigmasterol](https://www.tcmsp-e.com/molecule.php?qn=449) |
|  | Niuxi | Achyranthes bidentata Blume | Amaranthaceae | Achyranthes bidentata | Root | MOL012505 | bidentatoside,ii_qt |
|  |  |  |  |  |  | MOL000085 | beta-daucosterol_qt |
|  |  |  |  |  |  | MOL002643 | delta 7-stigmastenol |
|  |  |  |  |  |  | MOL004355 | Spinasterol |
|  |  |  |  |  |  | MOL000358 | beta-sitosterol |
|  |  |  |  |  |  | MOL001006 | poriferasta-7,22E-dien-3beta-ol |
|  |  |  |  |  |  | MOL000449 | Stigmasterol |
|  |  |  |  |  |  | MOL002897 | epiberberine |
|  |  |  |  |  |  | MOL001454 | berberine |
|  |  |  |  |  |  | MOL001458 | coptisine |
|  |  |  |  |  |  | MOL000098 | quercetin |
|  |  |  |  |  |  | MOL000422 | kaempferol |
|  |  |  |  |  |  | MOL003847 | Inophyllum E |
|  |  |  |  |  |  | MOL002714 | baicalein |
|  |  |  |  |  |  | MOL000173 | wogonin |
| Wen-Shen-Tong-Luo-Zhi-Tong-Decoction | Fuzi | Aconitum carmichaelii Debeaux | Ranunculaceae | Aconitum carmichaelii | Root | MOL002211 | 11,14-eicosadienoic acid |
|  |  |  |  |  |  | MOL002392 | Deltoin |
|  |  |  |  |  |  | MOL002397 | karakoline |
|  |  |  |  |  |  | MOL002398 | Karanjin |
|  |  |  |  |  |  | MOL002401 | Neokadsuranic acid B |
|  |  |  |  |  |  | MOL002410 | benzoylnapelline |
|  |  |  |  |  |  | MOL002415 | 6-Demethyldesoline |
|  |  |  |  |  |  | MOL002416 | deoxyaconitine |
|  |  |  |  |  |  | MOL002421 | ignavine |
|  |  |  |  |  |  | MOL002422 | isotalatizidine |
|  |  |  |  |  |  | MOL002434 | Carnosifloside I_qt |
|  |  |  |  |  |  | MOL000359 | sitosterol |
|  |  |  |  |  |  | MOL000538 | hypaconitine |
|  | Shanzhuyu | Cornus officinalis Siebold & Zucc. | Cornaceae | Cornus officinalis | Ripe fruit | MOL002883 | Ethyl oleate (NF) |
|  |  |  |  |  |  | MOL008457 | Tetrahydroalstonine |
|  |  |  |  |  |  | MOL005481 | 2,6,10,14,18-pentamethylicosa-2,6,10,14,18-pentaene |
|  |  |  |  |  |  | MOL005530 | Hydroxygenkwanin |
|  |  |  |  |  |  | MOL000359 | sitosterol |
|  |  |  |  |  |  | MOL001771 | poriferast-5-en-3beta-ol |
|  |  |  |  |  |  | MOL000358 | beta-sitosterol |
|  |  |  |  |  |  | MOL001494 | Mandenol |
|  |  |  |  |  |  | MOL000449 | Stigmasterol |
|  |  |  |  |  |  | MOL005557 | lanosta-8,24-dien-3-ol,3-acetate |
|  |  |  |  |  |  | MOL001495 | Ethyl linolenate |
|  |  |  |  |  |  | MOL005486 | 3,4-Dehydrolycopen-16-al |
|  |  |  |  |  |  | MOL005360 | malkangunin |
|  |  |  |  |  |  | MOL005531 | Telocinobufagin |
|  | Gusuibu | Drynaria roosii Nakaike | [Polypodiaceae](https://powo.science.kew.org/taxon/urn:lsid:ipni.org:names:30000471-2) | Drynaria roosii | Rhizome | MOL009087 | marioside_qt |
|  |  |  |  |  |  | MOL004328 | naringenin |
|  |  |  |  |  |  | MOL001040 | (2R)-5,7-dihydroxy-2-(4-hydroxyphenyl)chroman-4-one |
|  |  |  |  |  |  | MOL000422 | kaempferol |
|  |  |  |  |  |  | MOL002914 | Eriodyctiol (flavanone) |
|  |  |  |  |  |  | MOL005190 | eriodictyol |
|  |  |  |  |  |  | MOL001978 | Aureusidin |
|  |  |  |  |  |  | MOL000006 | luteolin |
|  |  |  |  |  |  | MOL009091 | xanthogalenol |
|  |  |  |  |  |  | MOL000358 | beta-sitosterol |
|  |  |  |  |  |  | MOL000449 | Stigmasterol |
|  |  |  |  |  |  | MOL009061 | 22-Stigmasten-3-one |
|  |  |  |  |  |  | MOL009075 | cycloartenone |
|  |  |  |  |  |  | MOL009063 | Cyclolaudenol acetate |
|  |  |  |  |  |  | MOL009076 | cyclolaudenol |
|  | Yinyanghuo | Epimedium brevicornu Maxim. | [Berberidaceae](https://powo.science.kew.org/taxon/urn:lsid:ipni.org:names:30000989-2) | Epimedium brevicornu | Leaves | MOL000098 | quercetin |
|  |  |  |  |  |  | MOL000006 | luteolin |
|  |  |  |  |  |  | MOL000422 | kaempferol |
|  |  |  |  |  |  | MOL004382 | Yinyanghuo A |
|  |  |  |  |  |  | MOL003044 | Chryseriol |
|  |  |  |  |  |  | MOL001792 | DFV |
|  |  |  |  |  |  | MOL004386 | Yinyanghuo E |
|  |  |  |  |  |  | MOL003542 | 8-Isopentenyl-kaempferol |
|  |  |  |  |  |  | MOL004373 | Anhydroicaritin |
|  |  |  |  |  |  | MOL004384 | Yinyanghuo C |
|  |  |  |  |  |  | MOL004380 | C-Homoerythrinan, 1,6-didehydro-3,15,16-trimethoxy-, (3.beta.)- |
|  |  |  |  |  |  | MOL000359 | sitosterol |
|  |  |  |  |  |  | MOL001645 | Linoleyl acetate |
|  |  |  |  |  |  | MOL001510 | 24-epicampesterol |
|  |  |  |  |  |  | MOL001771 | poriferast-5-en-3beta-ol |
|  |  |  |  |  |  | MOL004391 | 8-(3-methylbut-2-enyl)-2-phenyl-chromone |
|  | Shechuangzi | Cnidium monnieri (L.) Cusson | [Apiaceae](https://powo.science.kew.org/taxon/urn:lsid:ipni.org:names:30000180-2) | Cnidium monnieri | Fruit | MOL001510 | 24-epicampesterol |
|  |  |  |  |  |  | MOL001771 | poriferast-5-en-3beta-ol |
|  |  |  |  |  |  | MOL002881 | Diosmetin |
|  |  |  |  |  |  | MOL002883 | Ethyl oleate (NF) |
|  |  |  |  |  |  | MOL000358 | beta-sitosterol |
|  |  |  |  |  |  | MOL003591 | ar-curcumene |
|  |  |  |  |  |  | MOL003600 | cnidimol B |
|  |  |  |  |  |  | MOL003604 | cnidimol F |
|  |  |  |  |  |  | MOL003605 | (E)-2,3-bis(2-keto-7-methoxy-chromen-8-yl)acrolein |
|  |  |  |  |  |  | MOL003606 | cniforin A |
|  |  |  |  |  |  | MOL003607 | cniforin B |
|  |  |  |  |  |  | MOL003624 | o-Isovalerylcolum bianetin |
|  |  |  |  |  |  | MOL003626 | Ostruthin |
|  |  |  |  |  |  | MOL000449 | Stigmasterol |
|  | Gouji | Cibotium barometz (L.) J.Sm. | [Cyatheaceae](https://powo.science.kew.org/taxon/urn:lsid:ipni.org:names:77126785-1) | Cibotium barometz | Rhizome | MOL003389 | 3'-O-Methylorobol |
|  |  |  |  |  |  | MOL002608 | Aspidinol |
|  |  |  |  |  |  | MOL000448 | isobavachin |
|  |  |  |  |  |  | MOL000422 | kaempferol |
|  |  |  |  |  |  | MOL004328 | naringenin |
|  |  |  |  |  |  | MOL003673 | Wighteone |
|  | Yiyiren | Coix lacryma-jobi var. ma-yuen (Rom.Caill.) Stapf | [Poaceae](https://powo.science.kew.org/taxon/urn:lsid:ipni.org:names:30000032-2) | [Coix lacryma-jobi](https://powo.science.kew.org/taxon/urn:lsid:ipni.org:names:30100521-2) | Seeds | MOL001323 | Sitosterol alpha1 |
|  |  |  |  |  |  | MOL001494 | Mandenol |
|  |  |  |  |  |  | MOL002882 | [(2R)-2,3-dihydroxypropyl] (Z)-octadec-9-enoate |
|  |  |  |  |  |  | MOL000359 | sitosterol |
|  |  |  |  |  |  | MOL000449 | Stigmasterol |
|  |  |  |  |  |  | MOL008121 | 2-Monoolein |
|  |  |  |  |  |  | MOL000953 | CLR |
|  | Baizhu | Atractylodes macrocephala Koidz. | Asteraceae | Atractylodes macrocephala | Rhizome | MOL000033 | (3S,8S,9S,10R,13R,14S,17R)-10,13-dimethyl-17-[(2R,5S)-5-propan-2-yloctan-2-yl]-2,3,4,7,8,9,11,12,14,15,16,17-dodecahydro-1H-cyclopenta[a]phenanthren-3-ol |
|  |  |  |  |  |  | MOL000021 | 14-acetyl-12-senecioyl-2E,8E,10E-atractylentriol |
|  |  |  |  |  |  | MOL000020 | 12-senecioyl-2E,8E,10E-atractylentriol |
|  |  |  |  |  |  | MOL000022 | 14-acetyl-12-senecioyl-2E,8Z,10E-atractylentriol |
|  |  |  |  |  |  | MOL000072 | 8β-ethoxy atractylenolide Ⅲ |
|  | Qianghuo | Hansenia weberbaueriana (Fedde ex H.Wolff) Pimenov & Kljuykov | Apiaceae | Hansenia weberbaueriana | Rhizome and root | MOL000358 | beta-sitosterol |
|  |  |  |  |  |  | MOL000359 | sitosterol |
|  |  |  |  |  |  | MOL002881 | Diosmetin |
|  | Duhuo | Angelica biserrata (R.H.Shan & C.Q.Yuan) C.Q.Yuan & R.H.Shan | [Apiaceae](https://powo.science.kew.org/taxon/urn:lsid:ipni.org:names:30000180-2) | Angelica biserrata | Root | MOL000358 | [beta-sitosterol](https://www.tcmsp-e.com/molecule.php?qn=358) |
|  |  |  |  |  |  | MOL004780 | [Angelicone](https://www.tcmsp-e.com/molecule.php?qn=4780) |
|  | Xixin | Asarum heterotropoides F.Schmidt | [Aristolochiaceae](https://powo.science.kew.org/taxon/urn:lsid:ipni.org:names:30000909-2) | Asarum heterotropoides | Rhizome and root | MOL012140 | 4,9-dimethoxy-1-vinyl-$b-carboline |
|  |  |  |  |  |  | MOL012141 | Caribine |
|  |  |  |  |  |  | MOL001558 | sesamin |
|  |  |  |  |  |  | MOL009849 | ZINC05223929 |
|  |  |  |  |  |  | MOL000422 | kaempferol |
|  |  |  |  |  |  | MOL002962 | (3S)-7-hydroxy-3-(2,3,4-trimethoxyphenyl)chroman-4-one |
|  |  |  |  |  |  | MOL001460 | Cryptopin |
|  | Tianma | Gastrodia elata Blume | Orchidaceae | Gastrodia elata | Stem | MOL002307 | 20-Hexadecanoylingenol |
|  |  |  |  |  |  | MOL011455 | 20-Hexadecanoylingenol |
|  |  |  |  |  |  | MOL001320 | amygdalin |
|  |  |  |  |  |  | MOL000358 | beta-sitosterol |
|  |  |  |  |  |  | MOL001986 | beta-sitosterol |
|  |  |  |  |  |  | MOL001987 | beta-sitosterol |
|  |  |  |  |  |  | MOL008583 | beta-sitosterol |
|  |  |  |  |  |  | MOL001456 | citric acid |
|  |  |  |  |  |  | MOL000093 | daucosterol |
|  |  |  |  |  |  | MOL001525 | daucosterol |
|  |  |  |  |  |  | MOL002296 | daucosterol |
|  |  |  |  |  |  | MOL005868 | daucosterol |
|  |  |  |  |  |  | MOL007096 | daucosterol |
|  |  |  |  |  |  | MOL008172 | daucosterol |
|  |  |  |  |  |  | MOL008926 | daucosterol |
|  |  |  |  |  |  | MOL010584 | daucosterol |
|  |  |  |  |  |  | MOL012237 | daucosterol |
|  |  |  |  |  |  | MOL007986 | Gastrodin |
|  |  |  |  |  |  | MOL000346 | succinic acid |
|  |  |  |  |  |  | MOL005384 | suchilactone |
|  |  |  |  |  |  | MOL000842 | sucrose |
|  |  |  |  |  |  | MOL000635 | vanillin |
|  |  |  |  |  |  | MOL002647 | Vanillyl alcohol |
|  | Baishao | Paeonia lactiflora Pall. | Paeoniaceae | Paeonia lactiflora | Root | MOL000359 | sitosterol |
|  |  |  |  |  |  | MOL000358 | beta-sitosterol |
|  |  |  |  |  |  | MOL000422 | kaempferol |
|  |  |  |  |  |  | MOL001919 | (3S,5R,8R,9R,10S,14S)-3,17-dihydroxy-4,4,8,10,14-pentamethyl-2,3,5,6,7,9-hexahydro-1H-cyclopenta[a]phenanthrene-15,16-dione |
|  |  |  |  |  |  | MOL000211 | Mairin |
|  |  |  |  |  |  | MOL001925 | paeoniflorin_qt |
|  |  |  |  |  |  | MOL001918 | paeoniflorgenone |
|  | Gancao | Glycyrrhiza glabra L. | Fabaceae | Glycyrrhiza glabra | Rhizome and root | MOL001484 | Inermine |
|  |  |  |  |  |  | MOL001792 | DFV |
|  |  |  |  |  |  | MOL000211 | Mairin |
|  |  |  |  |  |  | MOL002311 | Glycyrol |
|  |  |  |  |  |  | MOL000239 | Jaranol |
|  |  |  |  |  |  | MOL002565 | Medicarpin |
|  |  |  |  |  |  | MOL000354 | isorhamnetin |
|  |  |  |  |  |  | MOL000359 | sitosterol |
|  |  |  |  |  |  | MOL003656 | Lupiwighteone |
|  |  |  |  |  |  | MOL003896 | 7-Methoxy-2-methyl isoflavone |
|  |  |  |  |  |  | MOL000392 | formononetin |
|  |  |  |  |  |  | MOL000417 | Calycosin |
|  |  |  |  |  |  | MOL000422 | kaempferol |
|  |  |  |  |  |  | MOL004328 | naringenin |
|  |  |  |  |  |  | MOL004805 | (2S)-2-[4-hydroxy-3-(3-methylbut-2-enyl)phenyl]-8,8-dimethyl-2,3-dihydropyrano[2,3-f]chromen-4-one |
|  |  |  |  |  |  | MOL004806 | euchrenone |
|  |  |  |  |  |  | MOL004808 | glyasperin B |
|  |  |  |  |  |  | MOL004810 | glyasperin F |
|  |  |  |  |  |  | MOL004814 | Isotrifoliol |
|  |  |  |  |  |  | MOL004815 | (E)-1-(2,4-dihydroxyphenyl)-3-(2,2-dimethylchromen-6-yl)prop-2-en-1-one |
|  |  |  |  |  |  | MOL004824 | (2S)-6-(2,4-dihydroxyphenyl)-2-(2-hydroxypropan-2-yl)-4-methoxy-2,3-dihydrofuro[3,2-g]chromen-7-one |
|  |  |  |  |  |  | MOL004827 | Semilicoisoflavone B |
|  |  |  |  |  |  | MOL004828 | Glepidotin A |
|  |  |  |  |  |  | MOL004829 | Glepidotin B |
|  |  |  |  |  |  | MOL004835 | Glypallichalcone |
|  |  |  |  |  |  | MOL004838 | 8-(6-hydroxy-2-benzofuranyl)-2,2-dimethyl-5-chromenol |
|  |  |  |  |  |  | MOL004841 | Licochalcone B |
|  |  |  |  |  |  | MOL004848 | licochalcone G |
|  |  |  |  |  |  | MOL004855 | Licoricone |
|  |  |  |  |  |  | MOL004856 | Gancaonin A |
|  |  |  |  |  |  | MOL004857 | Gancaonin B |
|  |  |  |  |  |  | MOL004863 | 3-(3,4-dihydroxyphenyl)-5,7-dihydroxy-8-(3-methylbut-2-enyl)chromone |
|  |  |  |  |  |  | MOL004864 | 5,7-dihydroxy-3-(4-methoxyphenyl)-8-(3-methylbut-2-enyl)chromone |
|  |  |  |  |  |  | MOL004866 | 2-(3,4-dihydroxyphenyl)-5,7-dihydroxy-6-(3-methylbut-2-enyl)chromone |
|  |  |  |  |  |  | MOL004882 | Licocoumarone |
|  |  |  |  |  |  | MOL004883 | Licoisoflavone |
|  |  |  |  |  |  | MOL004884 | Licoisoflavone B |
|  |  |  |  |  |  | MOL004885 | licoisoflavanone |
|  |  |  |  |  |  | MOL004891 | shinpterocarpin |
|  |  |  |  |  |  | MOL004898 | (E)-3-[3,4-dihydroxy-5-(3-methylbut-2-enyl)phenyl]-1-(2,4-dihydroxyphenyl)prop-2-en-1-one |
|  |  |  |  |  |  | MOL004907 | Glyzaglabrin |
|  |  |  |  |  |  | MOL004910 | Glabranin |
|  |  |  |  |  |  | MOL004912 | Glabrone |
|  |  |  |  |  |  | MOL004913 | 1,3-dihydroxy-9-methoxy-6-benzofurano[3,2-c]chromenone |
|  |  |  |  |  |  | MOL004914 | 1,3-dihydroxy-8,9-dimethoxy-6-benzofurano[3,2-c]chromenone |
|  |  |  |  |  |  | MOL004915 | Eurycarpin A |
|  |  |  |  |  |  | MOL004935 | Sigmoidin-B |
|  |  |  |  |  |  | MOL004941 | (2R)-7-hydroxy-2-(4-hydroxyphenyl)chroman-4-one |
|  |  |  |  |  |  | MOL004945 | (2S)-7-hydroxy-2-(4-hydroxyphenyl)-8-(3-methylbut-2-enyl)chroman-4-one |
|  |  |  |  |  |  | MOL004948 | Isoglycyrol |
|  |  |  |  |  |  | MOL004949 | Isolicoflavonol |
|  |  |  |  |  |  | MOL004957 | HMO |
|  |  |  |  |  |  | MOL004959 | 1-Methoxyphaseollidin |
|  |  |  |  |  |  | MOL004961 | Quercetin der. |
|  |  |  |  |  |  | MOL000497 | licochalcone a |
|  |  |  |  |  |  | MOL004985 | icos-5-enoic acid |
|  |  |  |  |  |  | MOL004988 | Kanzonol F |
|  |  |  |  |  |  | MOL004989 | 6-prenylated eriodictyol |
|  |  |  |  |  |  | MOL004991 | 7-Acetoxy-2-methylisoflavone |
|  |  |  |  |  |  | MOL004993 | 8-prenylated eriodictyol |
|  |  |  |  |  |  | MOL004996 | gadelaidic acid |
|  |  |  |  |  |  | MOL005000 | Gancaonin G |
|  |  |  |  |  |  | MOL005001 | Gancaonin H |
|  |  |  |  |  |  | MOL005003 | Licoagrocarpin |
|  |  |  |  |  |  | MOL005007 | Glyasperins M |
|  |  |  |  |  |  | MOL005008 | Glycyrrhiza flavonol A |
|  |  |  |  |  |  | MOL005012 | Licoagroisoflavone |
|  |  |  |  |  |  | MOL005013 | 18α-hydroxyglycyrrhetic acid |
|  |  |  |  |  |  | MOL005016 | Odoratin |
|  |  |  |  |  |  | MOL005017 | Phaseol |
|  |  |  |  |  |  | MOL005018 | Xambioona |
|  |  |  |  |  |  | MOL000098 | quercetin |
|  | Huangqi | [Astragalus mongholicus Bunge](https://mpns.science.kew.org/mpns-portal/plantDetail?plantId=2661222&query=Astragalus+membranaceus+%28Fisch.%29Bge.&filter=&fuzzy=false&nameType=all&dbs=wcs) | [Fabaceae](https://powo.science.kew.org/taxon/urn:lsid:ipni.org:names:30000147-2) | Astragalus mongholicus | Root | MOL000211 | Mairin |
|  |  |  |  |  |  | MOL000239 | Jaranol |
|  |  |  |  |  |  | MOL000296 | hederagenin |
|  |  |  |  |  |  | MOL000033 | (3S,8S,9S,10R,13R,14S,17R)-10,13-dimethyl-17-[(2R,5S)-5-propan-2-yloctan-2-yl]-2,3,4,7,8,9,11,12,14,15,16,17-dodecahydro-1H-cyclopenta[a]phenanthren-3-ol |
|  |  |  |  |  |  | MOL000354 | isorhamnetin |
|  |  |  |  |  |  | MOL000371 | 3,9-di-O-methylnissolin |
|  |  |  |  |  |  | MOL000380 | (6aR,11aR)-9,10-dimethoxy-6a,11a-dihydro-6H-benzofurano[3,2-c]chromen-3-ol |
|  |  |  |  |  |  | MOL000387 | Bifendate |
|  |  |  |  |  |  | MOL000392 | formononetin |
|  |  |  |  |  |  | MOL000398 | isoflavanone |
|  |  |  |  |  |  | MOL000417 | Calycosin |
|  |  |  |  |  |  | MOL000422 | kaempferol |
|  |  |  |  |  |  | MOL000442 | 1,7-Dihydroxy-3,9-dimethoxy pterocarpene |
|  |  |  |  |  |  | MOL000098 | quercetin |
| Shenyang Fang | Roucongrong | Cistanche deserticola Ma | Orobanchaceae | Cistanche deserticola | Stem | MOL000358 | [beta-sitosterol](https://www.tcmsp-e.com/molecule.php?qn=358) |
|  |  |  |  |  |  | MOL005320 | [arachidonate](https://www.tcmsp-e.com/molecule.php?qn=5320) |
|  |  |  |  |  |  | MOL005384 | [suchilactone](https://www.tcmsp-e.com/molecule.php?qn=5384) |
|  |  |  |  |  |  | MOL000098 | [quercetin](https://www.tcmsp-e.com/molecule.php?qn=98) |
|  |  |  |  |  |  | MOL008871 | [Marckine](https://www.tcmsp-e.com/molecule.php?qn=8871) |
|  | Yinyanghuo | Epimedium brevicornu Maxim. | [Berberidaceae](https://powo.science.kew.org/taxon/urn:lsid:ipni.org:names:30000989-2) | Epimedium brevicornu | Leaves | MOL000098 | quercetin |
|  |  |  |  |  |  | MOL000006 | luteolin |
|  |  |  |  |  |  | MOL000422 | kaempferol |
|  |  |  |  |  |  | MOL004382 | Yinyanghuo A |
|  |  |  |  |  |  | MOL003044 | Chryseriol |
|  |  |  |  |  |  | MOL001792 | DFV |
|  |  |  |  |  |  | MOL004386 | Yinyanghuo E |
|  |  |  |  |  |  | MOL003542 | 8-Isopentenyl-kaempferol |
|  |  |  |  |  |  | MOL004373 | Anhydroicaritin |
|  |  |  |  |  |  | MOL004384 | Yinyanghuo C |
|  |  |  |  |  |  | MOL004380 | C-Homoerythrinan, 1,6-didehydro-3,15,16-trimethoxy-, (3.beta.)- |
|  |  |  |  |  |  | MOL000359 | sitosterol |
|  |  |  |  |  |  | MOL001645 | Linoleyl acetate |
|  |  |  |  |  |  | MOL001510 | 24-epicampesterol |
|  |  |  |  |  |  | MOL001771 | poriferast-5-en-3beta-ol |
|  |  |  |  |  |  | MOL004391 | 8-(3-methylbut-2-enyl)-2-phenyl-chromone |
|  | Gusuibu | Drynaria roosii Nakaike | [Polypodiaceae](https://powo.science.kew.org/taxon/urn:lsid:ipni.org:names:30000471-2) | Drynaria roosii | Rhizome | MOL009087 | marioside_qt |
|  |  |  |  |  |  | MOL004328 | naringenin |
|  |  |  |  |  |  | MOL001040 | (2R)-5,7-dihydroxy-2-(4-hydroxyphenyl)chroman-4-one |
|  |  |  |  |  |  | MOL000422 | kaempferol |
|  |  |  |  |  |  | MOL002914 | Eriodyctiol (flavanone) |
|  |  |  |  |  |  | MOL005190 | eriodictyol |
|  |  |  |  |  |  | MOL001978 | Aureusidin |
|  |  |  |  |  |  | MOL000006 | luteolin |
|  |  |  |  |  |  | MOL009091 | xanthogalenol |
|  |  |  |  |  |  | MOL000358 | beta-sitosterol |
|  |  |  |  |  |  | MOL000449 | Stigmasterol |
|  |  |  |  |  |  | MOL009061 | 22-Stigmasten-3-one |
|  |  |  |  |  |  | MOL009075 | cycloartenone |
|  |  |  |  |  |  | MOL009063 | Cyclolaudenol acetate |
|  |  |  |  |  |  | MOL009076 | cyclolaudenol |
| Erzhi Wan | Nvzhenzi | Ligustrum lucidum W.T.Aiton | Oleaceae | Ligustrum lucidum | Ripe fruit | MOL000358 | beta-sitosterol |
|  |  |  |  |  |  | MOL000422 | kaempferol |
|  |  |  |  |  |  | MOL004576 | taxifolin |
|  |  |  |  |  |  | MOL005147 | Lucidumoside D_qt |
|  |  |  |  |  |  | MOL005169 | ((20S)-24-ene-3β,20-diol-3-acetate |
|  |  |  |  |  |  | MOL005190 | eriodictyol |
|  |  |  |  |  |  | MOL005209 | Lucidusculine |
|  |  |  |  |  |  | MOL000006 | luteolin |
|  |  |  |  |  |  | MOL000098 | quercetin |
|  | Mohanlian | Eclipta prostrata (L.) L. | Asteraceae | Eclipta prostrata | Whole plant | MOL003378 | 1,3,8,9-tetrahydroxybenzofurano[3,2-c]chromen-6-one |
|  |  |  |  |  |  | MOL001689 | acacetin |
|  |  |  |  |  |  | MOL000006 | luteolin |
|  |  |  |  |  |  | MOL003398 | Pratensein |
|  |  |  |  |  |  | MOL000098 | quercetin |
|  |  |  |  |  |  | MOL003404 | wedelolactone |
|  |  |  |  |  |  | MOL003389 | 3'-O-Methylorobol |
|  |  |  |  |  |  | MOL002975 | butin |
|  |  |  |  |  |  | MOL003402 | demethylwedelolactone |
| Gushudan | Yinyanghuo | Epimedium brevicornu Maxim. | [Berberidaceae](https://powo.science.kew.org/taxon/urn:lsid:ipni.org:names:30000989-2) | Epimedium brevicornu | Leaves | MOL000098 | quercetin |
|  |  |  |  |  |  | MOL000006 | luteolin |
|  |  |  |  |  |  | MOL000422 | kaempferol |
|  |  |  |  |  |  | MOL004382 | Yinyanghuo A |
|  |  |  |  |  |  | MOL003044 | Chryseriol |
|  |  |  |  |  |  | MOL001792 | DFV |
|  |  |  |  |  |  | MOL004386 | Yinyanghuo E |
|  |  |  |  |  |  | MOL003542 | 8-Isopentenyl-kaempferol |
|  |  |  |  |  |  | MOL004373 | Anhydroicaritin |
|  |  |  |  |  |  | MOL004384 | Yinyanghuo C |
|  |  |  |  |  |  | MOL004380 | C-Homoerythrinan, 1,6-didehydro-3,15,16-trimethoxy-, (3.beta.)- |
|  |  |  |  |  |  | MOL000359 | sitosterol |
|  |  |  |  |  |  | MOL001645 | Linoleyl acetate |
|  |  |  |  |  |  | MOL001510 | 24-epicampesterol |
|  |  |  |  |  |  | MOL001771 | poriferast-5-en-3beta-ol |
|  |  |  |  |  |  | MOL004391 | 8-(3-methylbut-2-enyl)-2-phenyl-chromone |
|  | Gusuibu | Drynaria roosii Nakaike | [Polypodiaceae](https://powo.science.kew.org/taxon/urn:lsid:ipni.org:names:30000471-2) | Drynaria roosii | Rhizome | MOL009087 | marioside_qt |
|  |  |  |  |  |  | MOL004328 | naringenin |
|  |  |  |  |  |  | MOL001040 | (2R)-5,7-dihydroxy-2-(4-hydroxyphenyl)chroman-4-one |
|  |  |  |  |  |  | MOL000422 | kaempferol |
|  |  |  |  |  |  | MOL002914 | Eriodyctiol (flavanone) |
|  |  |  |  |  |  | MOL005190 | eriodictyol |
|  |  |  |  |  |  | MOL001978 | Aureusidin |
|  |  |  |  |  |  | MOL000006 | luteolin |
|  |  |  |  |  |  | MOL009091 | xanthogalenol |
|  |  |  |  |  |  | MOL000358 | beta-sitosterol |
|  |  |  |  |  |  | MOL000449 | Stigmasterol |
|  |  |  |  |  |  | MOL009061 | 22-Stigmasten-3-one |
|  |  |  |  |  |  | MOL009075 | cycloartenone |
|  |  |  |  |  |  | MOL009063 | Cyclolaudenol acetate |
|  |  |  |  |  |  | MOL009076 | cyclolaudenol |
|  | Shechuangzi | Cnidium monnieri (L.) Cusson | [Apiaceae](https://powo.science.kew.org/taxon/urn:lsid:ipni.org:names:30000180-2) | Cnidium monnieri | Fruit | MOL001510 | 24-epicampesterol |
|  |  |  |  |  |  | MOL001771 | poriferast-5-en-3beta-ol |
|  |  |  |  |  |  | MOL002881 | Diosmetin |
|  |  |  |  |  |  | MOL002883 | Ethyl oleate (NF) |
|  |  |  |  |  |  | MOL000358 | beta-sitosterol |
|  |  |  |  |  |  | MOL003591 | ar-curcumene |
|  |  |  |  |  |  | MOL003600 | cnidimol B |
|  |  |  |  |  |  | MOL003604 | cnidimol F |
|  |  |  |  |  |  | MOL003605 | (E)-2,3-bis(2-keto-7-methoxy-chromen-8-yl)acrolein |
|  |  |  |  |  |  | MOL003606 | cniforin A |
|  |  |  |  |  |  | MOL003607 | cniforin B |
|  |  |  |  |  |  | MOL003624 | o-Isovalerylcolum bianetin |
|  |  |  |  |  |  | MOL003626 | Ostruthin |
|  |  |  |  |  |  | MOL000449 | Stigmasterol |
|  | Danshen | Salvia miltiorrhiza Bunge | [Lamiaceae](https://powo.science.kew.org/taxon/urn:lsid:ipni.org:names:30000097-2) | Salvia miltiorrhiza | Root | MOL001601 | 1,2,5,6-tetrahydrotanshinone |
|  |  |  |  |  |  | MOL001659 | Poriferasterol |
|  |  |  |  |  |  | MOL001771 | poriferast-5-en-3beta-ol |
|  |  |  |  |  |  | MOL002222 | sugiol |
|  |  |  |  |  |  | MOL002651 | Dehydrotanshinone II A |
|  |  |  |  |  |  | MOL000006 | luteolin |
|  |  |  |  |  |  | MOL007036 | 5,6-dihydroxy-7-isopropyl-1,1-dimethyl-2,3-dihydrophenanthren-4-one |
|  |  |  |  |  |  | MOL007041 | 2-isopropyl-8-methylphenanthrene-3,4-dione |
|  |  |  |  |  |  | MOL007045 | 3α-hydroxytanshinoneⅡa |
|  |  |  |  |  |  | MOL007048 | (E)-3-[2-(3,4-dihydroxyphenyl)-7-hydroxy-benzofuran-4-yl]acrylic acid |
|  |  |  |  |  |  | MOL007049 | 4-methylenemiltirone |
|  |  |  |  |  |  | MOL007050 | 2-(4-hydroxy-3-methoxyphenyl)-5-(3-hydroxypropyl)-7-methoxy-3-benzofurancarboxaldehyde |
|  |  |  |  |  |  | MOL007058 | formyltanshinone |
|  |  |  |  |  |  | MOL007059 | 3-beta-Hydroxymethyllenetanshiquinone |
|  |  |  |  |  |  | MOL007061 | Methylenetanshinquinone |
|  |  |  |  |  |  | MOL007068 | Przewaquinone B |
|  |  |  |  |  |  | MOL007069 | przewaquinone c |
|  |  |  |  |  |  | MOL007070 | (6S,7R)-6,7-dihydroxy-1,6-dimethyl-8,9-dihydro-7H-naphtho[8,7-g]benzofuran-10,11-dione |
|  |  |  |  |  |  | MOL007071 | przewaquinone f |
|  |  |  |  |  |  | MOL007077 | sclareol |
|  |  |  |  |  |  | MOL007079 | tanshinaldehyde |
|  |  |  |  |  |  | MOL007081 | Danshenol B |
|  |  |  |  |  |  | MOL007082 | Danshenol A |
|  |  |  |  |  |  | MOL007085 | Salvilenone |
|  |  |  |  |  |  | MOL007088 | cryptotanshinone |
|  |  |  |  |  |  | MOL007093 | dan-shexinkum d |
|  |  |  |  |  |  | MOL007094 | danshenspiroketallactone |
|  |  |  |  |  |  | MOL007098 | deoxyneocryptotanshinone |
|  |  |  |  |  |  | MOL007100 | dihydrotanshinlactone |
|  |  |  |  |  |  | MOL007101 | dihydrotanshinoneⅠ |
|  |  |  |  |  |  | MOL007108 | isocryptotanshi-none |
|  |  |  |  |  |  | MOL007111 | Isotanshinone II |
|  |  |  |  |  |  | MOL007115 | manool |
|  |  |  |  |  |  | MOL007118 | microstegiol |
|  |  |  |  |  |  | MOL007119 | miltionone Ⅰ |
|  |  |  |  |  |  | MOL007122 | Miltirone |
|  |  |  |  |  |  | MOL007124 | neocryptotanshinone ii |
|  |  |  |  |  |  | MOL007125 | neocryptotanshinone |
|  |  |  |  |  |  | MOL007127 | 1-methyl-8,9-dihydro-7H-naphtho[5,6-g]benzofuran-6,10,11-trione |
|  |  |  |  |  |  | MOL007130 | prolithospermic acid |
|  |  |  |  |  |  | MOL007140 | (Z)-3-[2-[(E)-2-(3,4-dihydroxyphenyl)vinyl]-3,4-dihydroxy-phenyl]acrylic acid |
|  |  |  |  |  |  | MOL007149 | NSC 122421 |
|  |  |  |  |  |  | MOL007150 | (6S)-6-hydroxy-1-methyl-6-methylol-8,9-dihydro-7H-naphtho[8,7-g]benzofuran-10,11-quinone |
|  |  |  |  |  |  | MOL007151 | Tanshindiol B |
|  |  |  |  |  |  | MOL007152 | Przewaquinone E |
|  |  |  |  |  |  | MOL007154 | tanshinone iia |
|  |  |  |  |  |  | MOL007155 | (6S)-6-(hydroxymethyl)-1,6-dimethyl-8,9-dihydro-7H-naphtho[8,7-g]benzofuran-10,11-dione |
|  |  |  |  |  |  | MOL007156 | tanshinone Ⅵ |
| Yi-Guan-Jian decoction | Dihuang | Rehmannia glutinosa (Gaertn.) Libosch. ex DC. | [Orobanchaceae](https://powo.science.kew.org/taxon/urn:lsid:ipni.org:names:30003446-2) | Rehmannia glutinosa | Root | MOL000359 | [sitosterol](https://www.tcmsp-e.com/molecule.php?qn=359) |
|  |  |  |  |  |  | MOL000449 | [Stigmasterol](https://www.tcmsp-e.com/molecule.php?qn=449) |
|  | Shashen | Glehnia littoralis (J.G.Cooper) F.Schmidt ex Miq. | Apiaceae | Glehnia littoralis | Root | MOL000358 | [beta-sitosterol](https://www.tcmsp-e.com/molecule.php?qn=358) |
|  |  |  |  |  |  | MOL000449 | [Stigmasterol](https://www.tcmsp-e.com/molecule.php?qn=449) |
|  |  |  |  |  |  | MOL000098 | [quercetin](https://www.tcmsp-e.com/molecule.php?qn=98) |
|  | Danggui | Angelica sinensis (Oliv.) Diels | Apiaceae | Angelica sinensis | Root | MOL000358 | [beta-sitosterol](https://www.tcmsp-e.com/molecule.php?qn=358) |
|  |  |  |  |  |  | MOL000449 | [Stigmasterol](https://www.tcmsp-e.com/molecule.php?qn=449) |
|  | Maidong | Ophiopogon japonicus (Thunb.) Ker Gawl. | Asparagaceae | Ophiopogon japonicus | Tuber | MOL002573 | β-patchoulene |
|  |  |  |  |  |  | MOL012883 | β-patchoulene |
|  |  |  |  |  |  | MOL000546 | diosgenin |
|  |  |  |  |  |  | MOL002687 | guanosine |
|  |  |  |  |  |  | MOL000263 | oleanolic acid |
|  |  |  |  |  |  | MOL001447 | oleanolic acid |
|  |  |  |  |  |  | MOL000449 | stigmasterol |
|  |  |  |  |  |  | MOL002045 | stigmasterol |
|  |  |  |  |  |  | MOL004187 | Stigmasterol-beta-D-glucoside |
|  |  |  |  |  |  | MOL000059 | uridine |
|  | Gouqizi | Lycium barbarum L. | Solanaceae | Lycium barbarum | Dried ripe fruit | MOL001323 | Sitosterol alpha1 |
|  |  |  |  |  |  | MOL003578 | Cycloartenol |
|  |  |  |  |  |  | MOL001494 | Mandenol |
|  |  |  |  |  |  | MOL001495 | Ethyl linolenate |
|  |  |  |  |  |  | MOL001979 | LAN |
|  |  |  |  |  |  | MOL000449 | Stigmasterol |
|  |  |  |  |  |  | MOL000358 | beta-sitosterol |
|  |  |  |  |  |  | MOL005438 | campesterol |
|  |  |  |  |  |  | MOL007449 | 24-methylidenelophenol |
|  |  |  |  |  |  | MOL008173 | daucosterol_qt |
|  |  |  |  |  |  | MOL008400 | glycitein |
|  |  |  |  |  |  | MOL000953 | CLR |
|  |  |  |  |  |  | MOL009604 | 14b-pregnane |
|  |  |  |  |  |  | MOL009612 | (24R)-4alpha-Methyl-24-ethylcholesta-7,25-dien-3beta-ylacetate |
|  |  |  |  |  |  | MOL009615 | 24-Methylenecycloartan-3beta,21-diol |
|  |  |  |  |  |  | MOL009617 | 24-ethylcholest-22-enol |
|  |  |  |  |  |  | MOL009618 | 24-ethylcholesta-5,22-dienol |
|  |  |  |  |  |  | MOL009620 | 24-methyl-31-norlanost-9(11)-enol |
|  |  |  |  |  |  | MOL009621 | 24-methylenelanost-8-enol |
|  |  |  |  |  |  | MOL009622 | Fucosterol |
|  |  |  |  |  |  | MOL009631 | 31-Norcyclolaudenol |
|  |  |  |  |  |  | MOL009633 | 31-norlanost-9(11)-enol |
|  |  |  |  |  |  | MOL009634 | 31-norlanosterol |
|  |  |  |  |  |  | MOL009635 | 4,24-methyllophenol |
|  |  |  |  |  |  | MOL009639 | Lophenol |
|  |  |  |  |  |  | MOL009640 | 4alpha,14alpha,24-trimethylcholesta-8,24-dienol |
|  |  |  |  |  |  | MOL009641 | 4alpha,24-dimethylcholesta-7,24-dienol |
|  |  |  |  |  |  | MOL009642 | 4alpha-methyl-24-ethylcholesta-7,24-dienol |
|  |  |  |  |  |  | MOL009644 | 6-Fluoroindole-7-Dehydrocholesterol |
|  |  |  |  |  |  | MOL009646 | 7-O-Methylluteolin-6-C-beta-glucoside_qt |
|  |  |  |  |  |  | MOL009650 | Atropine |
|  |  |  |  |  |  | MOL009651 | Cryptoxanthin monoepoxide |
|  |  |  |  |  |  | MOL009653 | Cycloeucalenol |
|  |  |  |  |  |  | MOL009656 | (E,E)-1-ethyl octadeca-3,13-dienoate |
|  |  |  |  |  |  | MOL009662 | Lantadene A |
|  |  |  |  |  |  | MOL009677 | lanost-8-en-3beta-ol |
|  |  |  |  |  |  | MOL009678 | lanost-8-enol |
|  |  |  |  |  |  | MOL009681 | Obtusifoliol |
|  |  |  |  |  |  | MOL000098 | quercetin |
|  | Chuanlianzi | Melia azedarach L. | Meliaceae | Melia azedarach | Ripe fruit | MOL001494 | Mandenol |
|  |  |  |  |  |  | MOL001495 | Ethyl linolenate |
|  |  |  |  |  |  | MOL002045 | Stigmasterol |
|  |  |  |  |  |  | MOL002047 | melianone |
|  |  |  |  |  |  | MOL002048 | Nimbolidin D |
|  |  |  |  |  |  | MOL002053 | Nimbolin A |
|  |  |  |  |  |  | MOL002056 | (E)-3-[(2S,3R)-2-(4-hydroxy-3-methoxy-phenyl)-7-methoxy-3-methylol-2,3-dihydrobenzofuran-5-yl]acrolein |
|  |  |  |  |  |  | MOL000098 | quercetin |
| Roucongrong Pill | Roucongrong | Cistanche deserticola Ma | Orobanchaceae | Cistanche deserticola | Stem | MOL000358 | beta-sitosterol |
|  |  |  |  |  |  | MOL005320 | [arachidonate](https://www.tcmsp-e.com/molecule.php?qn=5320) |
|  |  |  |  |  |  | MOL005384 | [suchilactone](https://www.tcmsp-e.com/molecule.php?qn=5384) |
|  |  |  |  |  |  | MOL000098 | [quercetin](https://www.tcmsp-e.com/molecule.php?qn=98) |
|  |  |  |  |  |  | MOL008871 | [Marckine](https://www.tcmsp-e.com/molecule.php?qn=8871) |
|  | Tusizi | Cuscuta chinensis Lam. | Convolvulaceae | Cuscuta chinensis | Seed | MOL001558 | [sesamin](https://www.tcmsp-e.com/molecule.php?qn=1558) |
|  |  |  |  |  |  | MOL000184 | [NSC63551](https://www.tcmsp-e.com/molecule.php?qn=184) |
|  |  |  |  |  |  | MOL000354 | [isorhamnetin](https://www.tcmsp-e.com/molecule.php?qn=354) |
|  |  |  |  |  |  | MOL000358 | [beta-sitosterol](https://www.tcmsp-e.com/molecule.php?qn=358) |
|  |  |  |  |  |  | MOL000422 | [kaempferol](https://www.tcmsp-e.com/molecule.php?qn=422) |
|  |  |  |  |  |  | MOL005043 | [campest-5-en-3beta-ol](https://www.tcmsp-e.com/molecule.php?qn=5043) |
|  |  |  |  |  |  | MOL005440 | [Isofucosterol](https://www.tcmsp-e.com/molecule.php?qn=5440) |
|  |  |  |  |  |  | MOL005944 | [matrine](https://www.tcmsp-e.com/molecule.php?qn=5944) |
|  |  |  |  |  |  | MOL006649 | [sophranol](https://www.tcmsp-e.com/molecule.php?qn=6649) |
|  |  |  |  |  |  | MOL000953 | [CLR](https://www.tcmsp-e.com/molecule.php?qn=953) |
|  |  |  |  |  |  | MOL000098 | [quercetin](https://www.tcmsp-e.com/molecule.php?qn=98) |
|  | Fuzi | Aconitum carmichaelii Debeaux | Ranunculaceae | Aconitum carmichaelii | Root | MOL002211 | 11,14-eicosadienoic acid |
|  |  |  |  |  |  | MOL002392 | Deltoin |
|  |  |  |  |  |  | MOL002397 | karakoline |
|  |  |  |  |  |  | MOL002398 | Karanjin |
|  |  |  |  |  |  | MOL002401 | Neokadsuranic acid B |
|  |  |  |  |  |  | MOL002410 | benzoylnapelline |
|  |  |  |  |  |  | MOL002415 | 6-Demethyldesoline |
|  |  |  |  |  |  | MOL002416 | deoxyaconitine |
|  |  |  |  |  |  | MOL002421 | ignavine |
|  |  |  |  |  |  | MOL002422 | isotalatizidine |
|  |  |  |  |  |  | MOL002434 | Carnosifloside I_qt |
|  |  |  |  |  |  | MOL000359 | sitosterol |
|  |  |  |  |  |  | MOL000538 | hypaconitine |
|  | Niuxi | Achyranthes bidentata Blume | Amaranthaceae | Achyranthes bidentata | Root | MOL012505 | bidentatoside,ii_qt |
|  |  |  |  |  |  | MOL000085 | beta-daucosterol_qt |
|  |  |  |  |  |  | MOL002643 | delta 7-stigmastenol |
|  |  |  |  |  |  | MOL004355 | Spinasterol |
|  |  |  |  |  |  | MOL000358 | beta-sitosterol |
|  |  |  |  |  |  | MOL001006 | poriferasta-7,22E-dien-3beta-ol |
|  |  |  |  |  |  | MOL000449 | Stigmasterol |
|  |  |  |  |  |  | MOL002897 | epiberberine |
|  |  |  |  |  |  | MOL001454 | berberine |
|  |  |  |  |  |  | MOL001458 | coptisine |
|  |  |  |  |  |  | MOL000098 | quercetin |
|  |  |  |  |  |  | MOL000422 | kaempferol |
|  |  |  |  |  |  | MOL003847 | Inophyllum E |
|  |  |  |  |  |  | MOL002714 | baicalein |
|  |  |  |  |  |  | MOL000173 | wogonin |
|  | Lurong | Cervus nippon Temminck | Cervidae | Cervus nippon | Velvet antler | MOL010919 | 17-beta-estradiol |
|  |  |  |  |  |  | MOL005307 | Adenosine triphosphate |
|  |  |  |  |  |  | MOL010581 | cephalin |
|  |  |  |  |  |  | MOL000987 | cholesterol |
|  |  |  |  |  |  | MOL002442 | Cholesteryl ferulate |
|  |  |  |  |  |  | MOL010921 | estrone |
|  |  |  |  |  |  | MOL001943 | lecithin |
|  |  |  |  |  |  | MOL008290 | lecithin |
|  |  |  |  |  |  | MOL011415 | lecithin |
|  |  |  |  |  |  | MOL008253 | sphingomyelin |
|  |  |  |  |  |  | MOL012938 | sphingomyelin |
| Jianguwan | Roucongrong | Cistanche deserticola Ma | Orobanchaceae | Cistanche deserticola | Stem | MOL000358 | beta-sitosterol |
|  |  |  |  |  |  | MOL005320 | [arachidonate](https://www.tcmsp-e.com/molecule.php?qn=5320) |
|  |  |  |  |  |  | MOL005384 | [suchilactone](https://www.tcmsp-e.com/molecule.php?qn=5384) |
|  |  |  |  |  |  | MOL000098 | [quercetin](https://www.tcmsp-e.com/molecule.php?qn=98) |
|  |  |  |  |  |  | MOL008871 | [Marckine](https://www.tcmsp-e.com/molecule.php?qn=8871) |
|  | Bixie | Dioscorea futschauensis Uline ex R.Knuth | Dioscoreaceae | Dioscorea futschauensis | Rhizome | MOL013233 | [EINECS 213-897-0](https://www.tcmsp-e.com/molecule.php?qn=13233) |
|  |  |  |  |  |  | MOL000546 | [diosgenin](https://www.tcmsp-e.com/molecule.php?qn=546) |
|  | Tusizi | Cuscuta chinensis Lam. | Convolvulaceae | Cuscuta chinensis | Seed | MOL001558 | [sesamin](https://www.tcmsp-e.com/molecule.php?qn=1558) |
|  |  |  |  |  |  | MOL000184 | [NSC63551](https://www.tcmsp-e.com/molecule.php?qn=184) |
|  |  |  |  |  |  | MOL000354 | [isorhamnetin](https://www.tcmsp-e.com/molecule.php?qn=354) |
|  |  |  |  |  |  | MOL000358 | [beta-sitosterol](https://www.tcmsp-e.com/molecule.php?qn=358) |
|  |  |  |  |  |  | MOL000422 | [kaempferol](https://www.tcmsp-e.com/molecule.php?qn=422) |
|  |  |  |  |  |  | MOL005043 | [campest-5-en-3beta-ol](https://www.tcmsp-e.com/molecule.php?qn=5043) |
|  |  |  |  |  |  | MOL005440 | [Isofucosterol](https://www.tcmsp-e.com/molecule.php?qn=5440) |
|  |  |  |  |  |  | MOL005944 | [matrine](https://www.tcmsp-e.com/molecule.php?qn=5944) |
|  |  |  |  |  |  | MOL006649 | [sophranol](https://www.tcmsp-e.com/molecule.php?qn=6649) |
|  |  |  |  |  |  | MOL000953 | [CLR](https://www.tcmsp-e.com/molecule.php?qn=953) |
|  |  |  |  |  |  | MOL000098 | [quercetin](https://www.tcmsp-e.com/molecule.php?qn=98) |
|  | Duzhong | Eucommia ulmoides Oliv. | Eucommiaceae | Eucommia ulmoides | Bark | MOL000211 | [Mairin](https://www.tcmsp-e.com/molecule.php?qn=211) |
|  |  |  |  |  |  | MOL000358 | [beta-sitosterol](https://www.tcmsp-e.com/molecule.php?qn=358) |
|  |  |  |  |  |  | MOL000422 | [kaempferol](https://www.tcmsp-e.com/molecule.php?qn=422) |
|  |  |  |  |  |  | MOL000443 | [Erythraline](https://www.tcmsp-e.com/molecule.php?qn=443) |
|  |  |  |  |  |  | MOL007059 | [3-beta-Hydroxymethyllenetanshiquinone](https://www.tcmsp-e.com/molecule.php?qn=7059) |
|  |  |  |  |  |  | MOL009015 | [(-)-Tabernemontanine](https://www.tcmsp-e.com/molecule.php?qn=9015) |
|  |  |  |  |  |  | MOL009027 | [Cyclopamine](https://www.tcmsp-e.com/molecule.php?qn=9027) |
|  |  |  |  |  |  | MOL009029 | [Dehydrodiconiferyl alcohol 4,gamma'-di-O-beta-D-glucopyanoside_qt](https://www.tcmsp-e.com/molecule.php?qn=9029) |
|  |  |  |  |  |  | MOL009042 | [Helenalin](https://www.tcmsp-e.com/molecule.php?qn=9042) |
|  |  |  |  |  |  | MOL009053 | [4-[(2S,3R)-5-[(E)-3-hydroxyprop-1-enyl]-7-methoxy-3-methylol-2,3-dihydrobenzofuran-2-yl]-2-methoxy-phenol](https://www.tcmsp-e.com/molecule.php?qn=9053) |
|  |  |  |  |  |  | MOL000098 | [quercetin](https://www.tcmsp-e.com/molecule.php?qn=98) |
|  |  |  |  |  |  | MOL002773 | [beta-carotene](https://www.tcmsp-e.com/molecule.php?qn=2773) |
|  |  |  |  |  |  | MOL008240 | [(E)-3-[4-[(1R,2R)-2-hydroxy-2-(4-hydroxy-3-methoxy-phenyl)-1-methylol-ethoxy]-3-methoxy-phenyl]acrolein](https://www.tcmsp-e.com/molecule.php?qn=8240) |
|  |  |  |  |  |  | MOL011604 | [Syringetin](https://www.tcmsp-e.com/molecule.php?qn=11604) |
| Jiangu granule | Yinyanghuo | Epimedium brevicornu Maxim. | [Berberidaceae](https://powo.science.kew.org/taxon/urn:lsid:ipni.org:names:30000989-2) | Epimedium brevicornu | Leaves | MOL000098 | quercetin |
|  |  |  |  |  |  | MOL000006 | luteolin |
|  |  |  |  |  |  | MOL000422 | kaempferol |
|  |  |  |  |  |  | MOL004382 | Yinyanghuo A |
|  |  |  |  |  |  | MOL003044 | Chryseriol |
|  |  |  |  |  |  | MOL001792 | DFV |
|  |  |  |  |  |  | MOL004386 | Yinyanghuo E |
|  |  |  |  |  |  | MOL003542 | 8-Isopentenyl-kaempferol |
|  |  |  |  |  |  | MOL004373 | Anhydroicaritin |
|  |  |  |  |  |  | MOL004384 | Yinyanghuo C |
|  |  |  |  |  |  | MOL004380 | C-Homoerythrinan, 1,6-didehydro-3,15,16-trimethoxy-, (3.beta.)- |
|  |  |  |  |  |  | MOL000359 | sitosterol |
|  |  |  |  |  |  | MOL001645 | Linoleyl acetate |
|  |  |  |  |  |  | MOL001510 | 24-epicampesterol |
|  |  |  |  |  |  | MOL001771 | poriferast-5-en-3beta-ol |
|  |  |  |  |  |  | MOL004391 | 8-(3-methylbut-2-enyl)-2-phenyl-chromone |
|  | Shanzhuyu | Cornus officinalis Siebold & Zucc. | Cornaceae | Cornus officinalis | Ripe fruit | MOL002883 | [Ethyl oleate (NF)](https://www.tcmsp-e.com/molecule.php?qn=2883) |
|  |  |  |  |  |  | MOL008457 | [Tetrahydroalstonine](https://www.tcmsp-e.com/molecule.php?qn=8457) |
|  |  |  |  |  |  | MOL005481 | [2,6,10,14,18-pentamethylicosa-2,6,10,14,18-pentaene](https://www.tcmsp-e.com/molecule.php?qn=5481) |
|  |  |  |  |  |  | MOL005530 | [Hydroxygenkwanin](https://www.tcmsp-e.com/molecule.php?qn=5530) |
|  |  |  |  |  |  | MOL000359 | [sitosterol](https://www.tcmsp-e.com/molecule.php?qn=359) |
|  |  |  |  |  |  | MOL001771 | [poriferast-5-en-3beta-ol](https://www.tcmsp-e.com/molecule.php?qn=1771) |
|  |  |  |  |  |  | MOL000358 | [beta-sitosterol](https://www.tcmsp-e.com/molecule.php?qn=358) |
|  |  |  |  |  |  | MOL001494 | [Mandenol](https://www.tcmsp-e.com/molecule.php?qn=1494) |
|  |  |  |  |  |  | MOL000449 | [Stigmasterol](https://www.tcmsp-e.com/molecule.php?qn=449) |
|  |  |  |  |  |  | MOL005557 | [lanosta-8,24-dien-3-ol,3-acetate](https://www.tcmsp-e.com/molecule.php?qn=5557) |
|  |  |  |  |  |  | MOL001495 | [Ethyl linolenate](https://www.tcmsp-e.com/molecule.php?qn=1495) |
|  |  |  |  |  |  | MOL005486 | [3,4-Dehydrolycopen-16-al](https://www.tcmsp-e.com/molecule.php?qn=5486) |
|  |  |  |  |  |  | MOL005360 | [malkangunin](https://www.tcmsp-e.com/molecule.php?qn=5360) |
|  |  |  |  |  |  | MOL005531 | [Telocinobufagin](https://www.tcmsp-e.com/molecule.php?qn=5531) |
|  | Shanyao | Dioscorea oppositifolia L. | Dioscoreaceae | Dioscorea oppositifolia | Rhizome | MOL001559 | [piperlonguminine](https://www.tcmsp-e.com/molecule.php?qn=1559) |
|  |  |  |  |  |  | MOL001736 | [(-)-taxifolin](https://www.tcmsp-e.com/molecule.php?qn=1736) |
|  |  |  |  |  |  | MOL000310 | [Denudatin B](https://www.tcmsp-e.com/molecule.php?qn=310) |
|  |  |  |  |  |  | MOL000322 | [Kadsurenone](https://www.tcmsp-e.com/molecule.php?qn=322) |
|  |  |  |  |  |  | MOL005429 | [hancinol](https://www.tcmsp-e.com/molecule.php?qn=5429) |
|  |  |  |  |  |  | MOL005430 | [hancinone C](https://www.tcmsp-e.com/molecule.php?qn=5430) |
|  |  |  |  |  |  | MOL005435 | [24-Methylcholest-5-enyl-3belta-O-glucopyranoside_qt](https://www.tcmsp-e.com/molecule.php?qn=5435) |
|  |  |  |  |  |  | MOL005438 | [campesterol](https://www.tcmsp-e.com/molecule.php?qn=5438) |
|  |  |  |  |  |  | MOL005440 | [Isofucosterol](https://www.tcmsp-e.com/molecule.php?qn=5440) |
|  |  |  |  |  |  | MOL000449 | [Stigmasterol](https://www.tcmsp-e.com/molecule.php?qn=449) |
|  |  |  |  |  |  | MOL005458 | [Dioscoreside C_qt](https://www.tcmsp-e.com/molecule.php?qn=5458) |
|  |  |  |  |  |  | MOL000546 | [diosgenin](https://www.tcmsp-e.com/molecule.php?qn=546) |
|  |  |  |  |  |  | MOL005461 | [Doradexanthin](https://www.tcmsp-e.com/molecule.php?qn=5461) |
|  |  |  |  |  |  | MOL005463 | [Methylcimicifugoside_qt](https://www.tcmsp-e.com/molecule.php?qn=5463) |
|  |  |  |  |  |  | MOL005465 | [AIDS180907](https://www.tcmsp-e.com/molecule.php?qn=5465) |
|  |  |  |  |  |  | MOL000953 | [CLR](https://www.tcmsp-e.com/molecule.php?qn=953) |
|  | Dangshen | Codonopsis pilosula (Franch.) Nannf. | [Campanulaceae](https://powo.science.kew.org/taxon/urn:lsid:ipni.org:names:30000171-2) | Codonopsis pilosula | Root | MOL001006 | [poriferasta-7,22E-dien-3beta-ol](https://www.tcmsp-e.com/molecule.php?qn=1006) |
|  |  |  |  |  |  | MOL002140 | [Perlolyrine](https://www.tcmsp-e.com/molecule.php?qn=2140) |
|  |  |  |  |  |  | MOL003036 | [ZINC03978781](https://www.tcmsp-e.com/molecule.php?qn=3036) |
|  |  |  |  |  |  | MOL000449 | [Stigmasterol](https://www.tcmsp-e.com/molecule.php?qn=449) |
|  |  |  |  |  |  | MOL003896 | [7-Methoxy-2-methyl isoflavone](https://www.tcmsp-e.com/molecule.php?qn=3896) |
|  |  |  |  |  |  | MOL004355 | [Spinasterol](https://www.tcmsp-e.com/molecule.php?qn=4355) |
|  |  |  |  |  |  | MOL004492 | [Chrysanthemaxanthin](https://www.tcmsp-e.com/molecule.php?qn=4492) |
|  |  |  |  |  |  | MOL005321 | [Frutinone A](https://www.tcmsp-e.com/molecule.php?qn=5321) |
|  |  |  |  |  |  | MOL000006 | [luteolin](https://www.tcmsp-e.com/molecule.php?qn=6) |
|  |  |  |  |  |  | MOL006774 | [stigmast-7-enol](https://www.tcmsp-e.com/molecule.php?qn=6774) |
|  |  |  |  |  |  | MOL007059 | [3-beta-Hydroxymethyllenetanshiquinone](https://www.tcmsp-e.com/molecule.php?qn=7059) |
|  |  |  |  |  |  | MOL007514 | [methyl icosa-11,14-dienoate](https://www.tcmsp-e.com/molecule.php?qn=7514) |
|  |  |  |  |  |  | MOL008391 | [5alpha-Stigmastan-3,6-dione](https://www.tcmsp-e.com/molecule.php?qn=8391) |
|  |  |  |  |  |  | MOL008397 | [Daturilin](https://www.tcmsp-e.com/molecule.php?qn=8397) |
|  |  |  |  |  |  | MOL008400 | [glycitein](https://www.tcmsp-e.com/molecule.php?qn=8400) |
|  |  |  |  |  |  | MOL008407 | [(8S,9S,10R,13R,14S,17R)-17-[(E,2R,5S)-5-ethyl-6-methylhept-3-en-2-yl]-10,13-dimethyl-1,2,4,7,8,9,11,12,14,15,16,17-dodecahydrocyclopenta[a]phenanthren-3-one](https://www.tcmsp-e.com/molecule.php?qn=8407) |
|  |  |  |  |  |  | MOL008411 | [11-Hydroxyrankinidine](https://www.tcmsp-e.com/molecule.php?qn=8411) |
|  | Gusuibu | Drynaria roosii Nakaike | [Polypodiaceae](https://powo.science.kew.org/taxon/urn:lsid:ipni.org:names:30000471-2) | Drynaria roosii | Rhizome | MOL009087 | marioside_qt |
|  |  |  |  |  |  | MOL004328 | naringenin |
|  |  |  |  |  |  | MOL001040 | (2R)-5,7-dihydroxy-2-(4-hydroxyphenyl)chroman-4-one |
|  |  |  |  |  |  | MOL000422 | kaempferol |
|  |  |  |  |  |  | MOL002914 | Eriodyctiol (flavanone) |
|  |  |  |  |  |  | MOL005190 | eriodictyol |
|  |  |  |  |  |  | MOL001978 | Aureusidin |
|  |  |  |  |  |  | MOL000006 | luteolin |
|  |  |  |  |  |  | MOL009091 | xanthogalenol |
|  |  |  |  |  |  | MOL000358 | beta-sitosterol |
|  |  |  |  |  |  | MOL000449 | Stigmasterol |
|  |  |  |  |  |  | MOL009061 | 22-Stigmasten-3-one |
|  |  |  |  |  |  | MOL009075 | cycloartenone |
|  |  |  |  |  |  | MOL009063 | Cyclolaudenol acetate |
|  |  |  |  |  |  | MOL009076 | cyclolaudenol |
|  | Gouqizi | Lycium barbarum L. | Solanaceae | Lycium barbarum | Dried ripe fruit | MOL001323 | Sitosterol alpha1 |
|  |  |  |  |  |  | MOL003578 | Cycloartenol |
|  |  |  |  |  |  | MOL001494 | Mandenol |
|  |  |  |  |  |  | MOL001495 | Ethyl linolenate |
|  |  |  |  |  |  | MOL001979 | LAN |
|  |  |  |  |  |  | MOL000449 | Stigmasterol |
|  |  |  |  |  |  | MOL000358 | beta-sitosterol |
|  |  |  |  |  |  | MOL005438 | campesterol |
|  |  |  |  |  |  | MOL007449 | 24-methylidenelophenol |
|  |  |  |  |  |  | MOL008173 | daucosterol_qt |
|  |  |  |  |  |  | MOL008400 | glycitein |
|  |  |  |  |  |  | MOL000953 | CLR |
|  |  |  |  |  |  | MOL009604 | 14b-pregnane |
|  |  |  |  |  |  | MOL009612 | (24R)-4alpha-Methyl-24-ethylcholesta-7,25-dien-3beta-ylacetate |
|  |  |  |  |  |  | MOL009615 | 24-Methylenecycloartan-3beta,21-diol |
|  |  |  |  |  |  | MOL009617 | 24-ethylcholest-22-enol |
|  |  |  |  |  |  | MOL009618 | 24-ethylcholesta-5,22-dienol |
|  |  |  |  |  |  | MOL009620 | 24-methyl-31-norlanost-9(11)-enol |
|  |  |  |  |  |  | MOL009621 | 24-methylenelanost-8-enol |
|  |  |  |  |  |  | MOL009622 | Fucosterol |
|  |  |  |  |  |  | MOL009631 | 31-Norcyclolaudenol |
|  |  |  |  |  |  | MOL009633 | 31-norlanost-9(11)-enol |
|  |  |  |  |  |  | MOL009634 | 31-norlanosterol |
|  |  |  |  |  |  | MOL009635 | 4,24-methyllophenol |
|  |  |  |  |  |  | MOL009639 | Lophenol |
|  |  |  |  |  |  | MOL009640 | 4alpha,14alpha,24-trimethylcholesta-8,24-dienol |
|  |  |  |  |  |  | MOL009641 | 4alpha,24-dimethylcholesta-7,24-dienol |
|  |  |  |  |  |  | MOL009642 | 4alpha-methyl-24-ethylcholesta-7,24-dienol |
|  |  |  |  |  |  | MOL009644 | 6-Fluoroindole-7-Dehydrocholesterol |
|  |  |  |  |  |  | MOL009646 | 7-O-Methylluteolin-6-C-beta-glucoside_qt |
|  |  |  |  |  |  | MOL009650 | Atropine |
|  |  |  |  |  |  | MOL009651 | Cryptoxanthin monoepoxide |
|  |  |  |  |  |  | MOL009653 | Cycloeucalenol |
|  |  |  |  |  |  | MOL009656 | (E,E)-1-ethyl octadeca-3,13-dienoate |
|  |  |  |  |  |  | MOL009662 | Lantadene A |
|  |  |  |  |  |  | MOL009677 | lanost-8-en-3beta-ol |
|  |  |  |  |  |  | MOL009678 | lanost-8-enol |
|  |  |  |  |  |  | MOL009681 | Obtusifoliol |
|  |  |  |  |  |  | MOL000098 | quercetin |
|  | Chenpi | Citrus reticulata Blanco | [Rutaceae](https://powo.science.kew.org/taxon/urn:lsid:ipni.org:names:30001492-2) | Citrus reticulata | Dried pericarp | MOL000359 | [sitosterol](https://www.tcmsp-e.com/molecule.php?qn=359) |
|  |  |  |  |  |  | MOL004328 | [naringenin](https://www.tcmsp-e.com/molecule.php?qn=4328) |
|  |  |  |  |  |  | MOL005100 | [5,7-dihydroxy-2-(3-hydroxy-4-methoxyphenyl)chroman-4-one](https://www.tcmsp-e.com/molecule.php?qn=5100) |
|  |  |  |  |  |  | MOL005815 | [Citromitin](https://www.tcmsp-e.com/molecule.php?qn=5815) |
|  |  |  |  |  |  | MOL005828 | [nobiletin](https://www.tcmsp-e.com/molecule.php?qn=5828) |
|  | Luxiancao | Pyrola calliantha Andres | [Ericaceae](https://powo.science.kew.org/taxon/urn:lsid:ipni.org:names:30000402-2) | Pyrola calliantha | Whole plant | MOL000358 | beta-sitosterol |
|  |  |  |  |  |  | MOL000359 | sitosterol |
|  |  |  |  |  |  | MOL000422 | kaempferol |
|  |  |  |  |  |  | MOL000552 | 5,2'-Dihydroxy-6,7,8-trimethoxyflavone |
|  |  |  |  |  |  | MOL000553 | (-)-Chimonanthine |
|  |  |  |  |  |  | MOL000098 | quercetin |
|  | Xihonghua | Croci Stigma | [Iridaceae](https://powo.science.kew.org/taxon/urn:lsid:ipni.org:names:30001783-2) | Crocus sativus | Stigma and style | MOL001389 | [n-heptanal](https://www.tcmsp-e.com/molecule.php?qn=1389) |
|  |  |  |  |  |  | MOL000354 | [isorhamnetin](https://www.tcmsp-e.com/molecule.php?qn=354) |
|  |  |  |  |  |  | MOL000422 | [kaempferol](https://www.tcmsp-e.com/molecule.php?qn=422) |
|  |  |  |  |  |  | MOL000098 | [quercetin](https://www.tcmsp-e.com/molecule.php?qn=98) |
|  | Jianghuang | Curcuma longa L. | [Zingiberaceae](https://powo.science.kew.org/taxon/urn:lsid:ipni.org:names:30004541-2) | Curcuma longa | Rhizome | MOL000449 | Stigmasterol |
|  |  |  |  |  |  | MOL000493 | campesterol |
|  |  |  |  |  |  | MOL000953 | CLR |
| Er-xian Decoction | Xianmao | Curculigo orchioides Gaertn. | [Hypoxidaceae](https://powo.science.kew.org/taxon/urn:lsid:ipni.org:names:77126735-1) | Curculigo orchioides | Root | MOL003578 | [Cycloartenol](https://www.tcmsp-e.com/molecule.php?qn=3578) |
|  |  |  |  |  |  | MOL000358 | [beta-sitosterol](https://www.tcmsp-e.com/molecule.php?qn=358) |
|  |  |  |  |  |  | MOL004114 | [3,2',4',6'-Tetrahydroxy-4,3'-dimethoxy chalcone](https://www.tcmsp-e.com/molecule.php?qn=4114) |
|  |  |  |  |  |  | MOL004125 | [Curculigoside B_qt](https://www.tcmsp-e.com/molecule.php?qn=4125) |
|  |  |  |  |  |  | MOL000449 | [Stigmasterol](https://www.tcmsp-e.com/molecule.php?qn=449) |
|  | Yinyanghuo | Epimedium brevicornu Maxim. | [Berberidaceae](https://powo.science.kew.org/taxon/urn:lsid:ipni.org:names:30000989-2) | Epimedium brevicornu | Leaves | MOL000098 | quercetin |
|  |  |  |  |  |  | MOL000006 | luteolin |
|  |  |  |  |  |  | MOL000422 | kaempferol |
|  |  |  |  |  |  | MOL004382 | Yinyanghuo A |
|  |  |  |  |  |  | MOL003044 | Chryseriol |
|  |  |  |  |  |  | MOL001792 | DFV |
|  |  |  |  |  |  | MOL004386 | Yinyanghuo E |
|  |  |  |  |  |  | MOL003542 | 8-Isopentenyl-kaempferol |
|  |  |  |  |  |  | MOL004373 | Anhydroicaritin |
|  |  |  |  |  |  | MOL004384 | Yinyanghuo C |
|  |  |  |  |  |  | MOL004380 | C-Homoerythrinan, 1,6-didehydro-3,15,16-trimethoxy-, (3.beta.)- |
|  |  |  |  |  |  | MOL000359 | sitosterol |
|  |  |  |  |  |  | MOL001645 | Linoleyl acetate |
|  |  |  |  |  |  | MOL001510 | 24-epicampesterol |
|  |  |  |  |  |  | MOL001771 | poriferast-5-en-3beta-ol |
|  |  |  |  |  |  | MOL004391 | 8-(3-methylbut-2-enyl)-2-phenyl-chromone |
|  | Bajitian | Gynochthodes officinalis (F.C.How) Razafim. & B.Bremer | Rubiaceae | Gynochthodes officinalis | Root | MOL002883 | [Ethyl oleate (NF)](https://www.tcmsp-e.com/molecule.php?qn=2883) |
|  |  |  |  |  |  | MOL000358 | [beta-sitosterol](https://www.tcmsp-e.com/molecule.php?qn=358) |
|  |  |  |  |  |  | MOL000359 | [sitosterol](https://www.tcmsp-e.com/molecule.php?qn=359) |
|  |  |  |  |  |  | MOL006147 | [Alizarin-2-methylether](https://www.tcmsp-e.com/molecule.php?qn=6147) |
|  |  |  |  |  |  | MOL009495 | [2-hydroxy-1,5-dimethoxy-6-(methoxymethyl)-9,10-anthraquinone](https://www.tcmsp-e.com/molecule.php?qn=9495) |
|  |  |  |  |  |  | MOL009496 | [1,5,7-trihydroxy-6-methoxy-2-methoxymethylanthracenequinone](https://www.tcmsp-e.com/molecule.php?qn=9496) |
|  |  |  |  |  |  | MOL009500 | [1,6-dihydroxy-5-methoxy-2-(methoxymethyl)-9,10-anthraquinone](https://www.tcmsp-e.com/molecule.php?qn=9500) |
|  |  |  |  |  |  | MOL009503 | [1-hydroxy-3-methoxy-9,10-anthraquinone](https://www.tcmsp-e.com/molecule.php?qn=9503) |
|  |  |  |  |  |  | MOL009504 | [1-hydroxy-6-hydroxymethylanthracenequinone](https://www.tcmsp-e.com/molecule.php?qn=9504) |
|  |  |  |  |  |  | MOL009513 | [2-hydroxy-1,8-dimethoxy-7-methoxymethylanthracenequinone](https://www.tcmsp-e.com/molecule.php?qn=9513) |
|  |  |  |  |  |  | MOL009519 | [(2R,3S)-(+)-3',5-Dihydroxy-4 ,7-dimethoxydihydroflavonol](https://www.tcmsp-e.com/molecule.php?qn=9519) |
|  |  |  |  |  |  | MOL009524 | [3beta,20(R),5-alkenyl-stigmastol](https://www.tcmsp-e.com/molecule.php?qn=9524) |
|  |  |  |  |  |  | MOL009525 | [3beta-24S(R)-butyl-5-alkenyl-cholestol](https://www.tcmsp-e.com/molecule.php?qn=9525) |
|  |  |  |  |  |  | MOL009562 | [Ohioensin-A](https://www.tcmsp-e.com/molecule.php?qn=9562) |
|  | Danggui | Angelica sinensis (Oliv.) Diels | Apiaceae | Angelica sinensis | Root | MOL000358 | [beta-sitosterol](https://www.tcmsp-e.com/molecule.php?qn=358) |
|  |  |  |  |  |  | MOL000449 | [Stigmasterol](https://www.tcmsp-e.com/molecule.php?qn=449) |
|  | Zhimu | Anemarrhena asphodeloides Bunge | Asparagaceae | Anemarrhena asphodeloides | Rhizome | MOL001677 | asperglaucide |
|  |  |  |  |  |  | MOL003773 | Mangiferolic acid |
|  |  |  |  |  |  | MOL000422 | kaempferol |
|  |  |  |  |  |  | MOL004373 | Anhydroicaritin |
|  |  |  |  |  |  | MOL004489 | Anemarsaponin F_qt |
|  |  |  |  |  |  | MOL004492 | Chrysanthemaxanthin |
|  |  |  |  |  |  | MOL004497 | Hippeastrine |
|  |  |  |  |  |  | MOL004514 | Timosaponin B III_qt |
|  |  |  |  |  |  | MOL000449 | Stigmasterol |
|  |  |  |  |  |  | MOL004542 | Anemarsaponin E_qt |
|  |  |  |  |  |  | MOL000483 | (Z)-3-(4-hydroxy-3-methoxy-phenyl)-N-[2-(4-hydroxyphenyl)ethyl]acrylamide |
|  |  |  |  |  |  | MOL000546 | diosgenin |
|  |  |  |  |  |  | MOL000631 | coumaroyltyramine |
|  | Huangbo | Phellodendron chinense C.K.Schneid. | Rutaceae | Phellodendron chinense | Bark | MOL002652 | delta7-Dehydrosophoramine |
|  |  |  |  |  |  | MOL000098 | quercetin |
|  |  |  |  |  |  | MOL001131 | phellamurin_qt |
|  |  |  |  |  |  | MOL002651 | Dehydrotanshinone II A |
|  |  |  |  |  |  | MOL002662 | rutaecarpine |
|  |  |  |  |  |  | MOL000762 | Palmidin A |
|  |  |  |  |  |  | MOL005438 | campesterol |
|  |  |  |  |  |  | MOL006422 | thalifendine |
|  |  |  |  |  |  | MOL002894 | berberrubine |
|  |  |  |  |  |  | MOL001771 | poriferast-5-en-3beta-ol |
|  |  |  |  |  |  | MOL002643 | delta 7-stigmastenol |
|  |  |  |  |  |  | MOL000358 | beta-sitosterol |
|  |  |  |  |  |  | MOL000449 | Stigmasterol |
|  |  |  |  |  |  | MOL001455 | (S)-Canadine |
|  |  |  |  |  |  | MOL001454 | berberine |
|  |  |  |  |  |  | MOL006401 | melianone |
|  |  |  |  |  |  | MOL002666 | Chelerythrine |
|  |  |  |  |  |  | MOL002670 | Cavidine |
|  |  |  |  |  |  | MOL002656 | dihydroniloticin |
|  |  |  |  |  |  | MOL006392 | dihydroniloticin |
|  |  |  |  |  |  | MOL006413 | phellochin |
|  |  |  |  |  |  | MOL002660 | niloticin |
|  |  |  |  |  |  | MOL000787 | Fumarine |
|  |  |  |  |  |  | MOL002673 | Hispidone |
|  |  |  |  |  |  | MOL001458 | coptisine |
|  |  |  |  |  |  | MOL002668 | Worenine |
| Zhuanggu Zhitong Formula | Yinyanghuo | Epimedium brevicornu Maxim. | [Berberidaceae](https://powo.science.kew.org/taxon/urn:lsid:ipni.org:names:30000989-2) | Epimedium brevicornu | Leaves | MOL000098 | quercetin |
|  |  |  |  |  |  | MOL000006 | luteolin |
|  |  |  |  |  |  | MOL000422 | kaempferol |
|  |  |  |  |  |  | MOL004382 | Yinyanghuo A |
|  |  |  |  |  |  | MOL003044 | Chryseriol |
|  |  |  |  |  |  | MOL001792 | DFV |
|  |  |  |  |  |  | MOL004386 | Yinyanghuo E |
|  |  |  |  |  |  | MOL003542 | 8-Isopentenyl-kaempferol |
|  |  |  |  |  |  | MOL004373 | Anhydroicaritin |
|  |  |  |  |  |  | MOL004384 | Yinyanghuo C |
|  |  |  |  |  |  | MOL004380 | C-Homoerythrinan, 1,6-didehydro-3,15,16-trimethoxy-, (3.beta.)- |
|  |  |  |  |  |  | MOL000359 | sitosterol |
|  |  |  |  |  |  | MOL001645 | Linoleyl acetate |
|  |  |  |  |  |  | MOL001510 | 24-epicampesterol |
|  |  |  |  |  |  | MOL001771 | poriferast-5-en-3beta-ol |
|  |  |  |  |  |  | MOL004391 | 8-(3-methylbut-2-enyl)-2-phenyl-chromone |
|  | Buguzhi | Cullen corylifolium (L.) Medik. | Fabaceae | Cullen corylifolium | Dried ripe fruit | MOL003590 | angelicin |
|  |  |  |  |  |  | MOL005009 | Corylifolinin |
|  |  |  |  |  |  | MOL000093 | daucosterol |
|  |  |  |  |  |  | MOL001525 | daucosterol |
|  |  |  |  |  |  | MOL002296 | daucosterol |
|  |  |  |  |  |  | MOL005868 | daucosterol |
|  |  |  |  |  |  | MOL007096 | daucosterol |
|  |  |  |  |  |  | MOL008172 | daucosterol |
|  |  |  |  |  |  | MOL008926 | daucosterol |
|  |  |  |  |  |  | MOL010584 | daucosterol |
|  |  |  |  |  |  | MOL012237 | daucosterol |
|  |  |  |  |  |  | MOL000448 | isobavachin |
|  |  |  |  |  |  | MOL001393 | myristic acid |
|  |  |  |  |  |  | MOL005639 | Neobavachalcone |
|  |  |  |  |  |  | MOL001950 | psoralen |
|  |  |  |  |  |  | MOL000860 | stearic acid |
|  |  |  |  |  |  | MOL000449 | stigmasterol |
|  |  |  |  |  |  | MOL002045 | stigmasterol |
|  |  |  |  |  |  | MOL008159 | TRIACONTANE |
|  | Gouji | Cibotium barometz （L.） J.Sm. | Cyatheaceae | Cibotium barometz | Rhizome | MOL003389 | 3'-O-Methylorobol |
|  |  |  |  |  |  | MOL002608 | Aspidinol |
|  |  |  |  |  |  | MOL000448 | isobavachin |
|  |  |  |  |  |  | MOL000422 | kaempferol |
|  |  |  |  |  |  | MOL004328 | naringenin |
|  |  |  |  |  |  | MOL003673 | Wighteone |
|  | Niuxi | Achyranthes bidentata Blume | Amaranthaceae | Achyranthes bidentata |  | MOL012505 | bidentatoside,ii_qt |
|  |  |  |  |  |  | MOL000085 | beta-daucosterol_qt |
|  |  |  |  |  |  | MOL002643 | delta 7-stigmastenol |
|  |  |  |  |  |  | MOL004355 | Spinasterol |
|  |  |  |  |  |  | MOL000358 | beta-sitosterol |
|  |  |  |  |  |  | MOL001006 | poriferasta-7,22E-dien-3beta-ol |
|  |  |  |  |  |  | MOL000449 | Stigmasterol |
|  |  |  |  |  |  | MOL002897 | epiberberine |
|  |  |  |  |  |  | MOL001454 | berberine |
|  |  |  |  |  |  | MOL001458 | coptisine |
|  |  |  |  |  |  | MOL000098 | quercetin |
|  |  |  |  |  |  | MOL000422 | kaempferol |
|  |  |  |  |  |  | MOL003847 | Inophyllum E |
|  |  |  |  |  |  | MOL002714 | baicalein |
|  |  |  |  |  |  | MOL000173 | wogonin |
|  | Gusuibu | Drynaria roosii Nakaike | [Polypodiaceae](https://powo.science.kew.org/taxon/urn:lsid:ipni.org:names:30000471-2) | Drynaria roosii | Rhizome | MOL009087 | marioside_qt |
|  |  |  |  |  |  | MOL004328 | naringenin |
|  |  |  |  |  |  | MOL001040 | (2R)-5,7-dihydroxy-2-(4-hydroxyphenyl)chroman-4-one |
|  |  |  |  |  |  | MOL000422 | kaempferol |
|  |  |  |  |  |  | MOL002914 | Eriodyctiol (flavanone) |
|  |  |  |  |  |  | MOL005190 | eriodictyol |
|  |  |  |  |  |  | MOL001978 | Aureusidin |
|  |  |  |  |  |  | MOL000006 | luteolin |
|  |  |  |  |  |  | MOL009091 | xanthogalenol |
|  |  |  |  |  |  | MOL000358 | beta-sitosterol |
|  |  |  |  |  |  | MOL000449 | Stigmasterol |
|  |  |  |  |  |  | MOL009061 | 22-Stigmasten-3-one |
|  |  |  |  |  |  | MOL009075 | cycloartenone |
|  |  |  |  |  |  | MOL009063 | Cyclolaudenol acetate |
|  |  |  |  |  |  | MOL009076 | cyclolaudenol |
|  | Gouqizi | Lycium barbarum L. | Solanaceae | Lycium barbarum | Dried ripe fruit | MOL001323 | Sitosterol alpha1 |
|  |  |  |  |  |  | MOL003578 | Cycloartenol |
|  |  |  |  |  |  | MOL001494 | Mandenol |
|  |  |  |  |  |  | MOL001495 | Ethyl linolenate |
|  |  |  |  |  |  | MOL001979 | LAN |
|  |  |  |  |  |  | MOL000449 | Stigmasterol |
|  |  |  |  |  |  | MOL000358 | beta-sitosterol |
|  |  |  |  |  |  | MOL005438 | campesterol |
|  |  |  |  |  |  | MOL007449 | 24-methylidenelophenol |
|  |  |  |  |  |  | MOL008173 | daucosterol_qt |
|  |  |  |  |  |  | MOL008400 | glycitein |
|  |  |  |  |  |  | MOL000953 | CLR |
|  |  |  |  |  |  | MOL009604 | 14b-pregnane |
|  |  |  |  |  |  | MOL009612 | (24R)-4alpha-Methyl-24-ethylcholesta-7,25-dien-3beta-ylacetate |
|  |  |  |  |  |  | MOL009615 | 24-Methylenecycloartan-3beta,21-diol |
|  |  |  |  |  |  | MOL009617 | 24-ethylcholest-22-enol |
|  |  |  |  |  |  | MOL009618 | 24-ethylcholesta-5,22-dienol |
|  |  |  |  |  |  | MOL009620 | 24-methyl-31-norlanost-9(11)-enol |
|  |  |  |  |  |  | MOL009621 | 24-methylenelanost-8-enol |
|  |  |  |  |  |  | MOL009622 | Fucosterol |
|  |  |  |  |  |  | MOL009631 | 31-Norcyclolaudenol |
|  |  |  |  |  |  | MOL009633 | 31-norlanost-9(11)-enol |
|  |  |  |  |  |  | MOL009634 | 31-norlanosterol |
|  |  |  |  |  |  | MOL009635 | 4,24-methyllophenol |
|  |  |  |  |  |  | MOL009639 | Lophenol |
|  |  |  |  |  |  | MOL009640 | 4alpha,14alpha,24-trimethylcholesta-8,24-dienol |
|  |  |  |  |  |  | MOL009641 | 4alpha,24-dimethylcholesta-7,24-dienol |
|  |  |  |  |  |  | MOL009642 | 4alpha-methyl-24-ethylcholesta-7,24-dienol |
|  |  |  |  |  |  | MOL009644 | 6-Fluoroindole-7-Dehydrocholesterol |
|  |  |  |  |  |  | MOL009646 | 7-O-Methylluteolin-6-C-beta-glucoside_qt |
|  |  |  |  |  |  | MOL009650 | Atropine |
|  |  |  |  |  |  | MOL009651 | Cryptoxanthin monoepoxide |
|  |  |  |  |  |  | MOL009653 | Cycloeucalenol |
|  |  |  |  |  |  | MOL009656 | (E,E)-1-ethyl octadeca-3,13-dienoate |
|  |  |  |  |  |  | MOL009662 | Lantadene A |
|  |  |  |  |  |  | MOL009677 | lanost-8-en-3beta-ol |
|  |  |  |  |  |  | MOL009678 | lanost-8-enol |
|  |  |  |  |  |  | MOL009681 | Obtusifoliol |
|  |  |  |  |  |  | MOL000098 | quercetin |
|  | Nvzhenzi | Ligustrum lucidum W.T.Aiton | Oleaceae | Ligustrum lucidum | Ripe fruit | MOL000358 | beta-sitosterol |
|  |  |  |  |  |  | MOL000422 | kaempferol |
|  |  |  |  |  |  | MOL004576 | taxifolin |
|  |  |  |  |  |  | MOL005147 | Lucidumoside D_qt |
|  |  |  |  |  |  | MOL005169 | ((20S)-24-ene-3β,20-diol-3-acetate |
|  |  |  |  |  |  | MOL005190 | eriodictyol |
|  |  |  |  |  |  | MOL005209 | Lucidusculine |
|  |  |  |  |  |  | MOL000006 | luteolin |
|  |  |  |  |  |  | MOL000098 | quercetin |
| Yigu decoction | Buguzhi | Cullen corylifolium (L.) Medik. | Fabaceae | Cullen corylifolium | Dried ripe fruit | MOL003590 | angelicin |
|  |  |  |  |  |  | MOL005009 | Corylifolinin |
|  |  |  |  |  |  | MOL000093 | daucosterol |
|  |  |  |  |  |  | MOL001525 | daucosterol |
|  |  |  |  |  |  | MOL002296 | daucosterol |
|  |  |  |  |  |  | MOL005868 | daucosterol |
|  |  |  |  |  |  | MOL007096 | daucosterol |
|  |  |  |  |  |  | MOL008172 | daucosterol |
|  |  |  |  |  |  | MOL008926 | daucosterol |
|  |  |  |  |  |  | MOL010584 | daucosterol |
|  |  |  |  |  |  | MOL012237 | daucosterol |
|  |  |  |  |  |  | MOL000448 | isobavachin |
|  |  |  |  |  |  | MOL001393 | myristic acid |
|  |  |  |  |  |  | MOL005639 | Neobavachalcone |
|  |  |  |  |  |  | MOL001950 | psoralen |
|  |  |  |  |  |  | MOL000860 | stearic acid |
|  |  |  |  |  |  | MOL000449 | stigmasterol |
|  |  |  |  |  |  | MOL002045 | stigmasterol |
|  |  |  |  |  |  | MOL008159 | TRIACONTANE |
|  | Gusuibu | Drynaria roosii Nakaike | Polypodiaceae | Drynaria roosii | Rhizome | MOL009087 | marioside_qt |
|  |  |  |  |  |  | MOL004328 | naringenin |
|  |  |  |  |  |  | MOL001040 | (2R)-5,7-dihydroxy-2-(4-hydroxyphenyl)chroman-4-one |
|  |  |  |  |  |  | MOL000422 | kaempferol |
|  |  |  |  |  |  | MOL002914 | Eriodyctiol (flavanone) |
|  |  |  |  |  |  | MOL005190 | eriodictyol |
|  |  |  |  |  |  | MOL001978 | Aureusidin |
|  |  |  |  |  |  | MOL000006 | luteolin |
|  |  |  |  |  |  | MOL009091 | xanthogalenol |
|  |  |  |  |  |  | MOL000358 | beta-sitosterol |
|  |  |  |  |  |  | MOL000449 | Stigmasterol |
|  |  |  |  |  |  | MOL009061 | 22-Stigmasten-3-one |
|  |  |  |  |  |  | MOL009075 | cycloartenone |
|  |  |  |  |  |  | MOL009063 | Cyclolaudenol acetate |
|  |  |  |  |  |  | MOL009076 | cyclolaudenol |
|  | Yinyanghuo | Epimedium brevicornu Maxim. | Berberidaceae | Epimedium brevicornu | Leaves | MOL000098 | quercetin |
|  |  |  |  |  |  | MOL000006 | luteolin |
|  |  |  |  |  |  | MOL000422 | kaempferol |
|  |  |  |  |  |  | MOL004382 | Yinyanghuo A |
|  |  |  |  |  |  | MOL003044 | Chryseriol |
|  |  |  |  |  |  | MOL001792 | DFV |
|  |  |  |  |  |  | MOL004386 | Yinyanghuo E |
|  |  |  |  |  |  | MOL003542 | 8-Isopentenyl-kaempferol |
|  |  |  |  |  |  | MOL004373 | Anhydroicaritin |
|  |  |  |  |  |  | MOL004384 | Yinyanghuo C |
|  |  |  |  |  |  | MOL004380 | C-Homoerythrinan, 1,6-didehydro-3,15,16-trimethoxy-, (3.beta.)- |
|  |  |  |  |  |  | MOL000359 | sitosterol |
|  |  |  |  |  |  | MOL001645 | Linoleyl acetate |
|  |  |  |  |  |  | MOL001510 | 24-epicampesterol |
|  |  |  |  |  |  | MOL001771 | poriferast-5-en-3beta-ol |
|  |  |  |  |  |  | MOL004391 | 8-(3-methylbut-2-enyl)-2-phenyl-chromone |
|  | Danshen | Salvia miltiorrhiza Bunge | [Lamiaceae](https://powo.science.kew.org/taxon/urn:lsid:ipni.org:names:30000097-2) | Salvia miltiorrhiza | Root | MOL001601 | 1,2,5,6-tetrahydrotanshinone |
|  |  |  |  |  |  | MOL001659 | Poriferasterol |
|  |  |  |  |  |  | MOL001771 | poriferast-5-en-3beta-ol |
|  |  |  |  |  |  | MOL002222 | sugiol |
|  |  |  |  |  |  | MOL002651 | Dehydrotanshinone II A |
|  |  |  |  |  |  | MOL000006 | luteolin |
|  |  |  |  |  |  | MOL007036 | 5,6-dihydroxy-7-isopropyl-1,1-dimethyl-2,3-dihydrophenanthren-4-one |
|  |  |  |  |  |  | MOL007041 | 2-isopropyl-8-methylphenanthrene-3,4-dione |
|  |  |  |  |  |  | MOL007045 | 3α-hydroxytanshinoneⅡa |
|  |  |  |  |  |  | MOL007048 | (E)-3-[2-(3,4-dihydroxyphenyl)-7-hydroxy-benzofuran-4-yl]acrylic acid |
|  |  |  |  |  |  | MOL007049 | 4-methylenemiltirone |
|  |  |  |  |  |  | MOL007050 | 2-(4-hydroxy-3-methoxyphenyl)-5-(3-hydroxypropyl)-7-methoxy-3-benzofurancarboxaldehyde |
|  |  |  |  |  |  | MOL007058 | formyltanshinone |
|  |  |  |  |  |  | MOL007059 | 3-beta-Hydroxymethyllenetanshiquinone |
|  |  |  |  |  |  | MOL007061 | Methylenetanshinquinone |
|  |  |  |  |  |  | MOL007068 | Przewaquinone B |
|  |  |  |  |  |  | MOL007069 | przewaquinone c |
|  |  |  |  |  |  | MOL007070 | (6S,7R)-6,7-dihydroxy-1,6-dimethyl-8,9-dihydro-7H-naphtho[8,7-g]benzofuran-10,11-dione |
|  |  |  |  |  |  | MOL007071 | przewaquinone f |
|  |  |  |  |  |  | MOL007077 | sclareol |
|  |  |  |  |  |  | MOL007079 | tanshinaldehyde |
|  |  |  |  |  |  | MOL007081 | Danshenol B |
|  |  |  |  |  |  | MOL007082 | Danshenol A |
|  |  |  |  |  |  | MOL007085 | Salvilenone |
|  |  |  |  |  |  | MOL007088 | cryptotanshinone |
|  |  |  |  |  |  | MOL007093 | dan-shexinkum d |
|  |  |  |  |  |  | MOL007094 | danshenspiroketallactone |
|  |  |  |  |  |  | MOL007098 | deoxyneocryptotanshinone |
|  |  |  |  |  |  | MOL007100 | dihydrotanshinlactone |
|  |  |  |  |  |  | MOL007101 | dihydrotanshinoneⅠ |
|  |  |  |  |  |  | MOL007108 | isocryptotanshi-none |
|  |  |  |  |  |  | MOL007111 | Isotanshinone II |
|  |  |  |  |  |  | MOL007115 | manool |
|  |  |  |  |  |  | MOL007118 | microstegiol |
|  |  |  |  |  |  | MOL007119 | miltionone Ⅰ |
|  |  |  |  |  |  | MOL007122 | Miltirone |
|  |  |  |  |  |  | MOL007124 | neocryptotanshinone ii |
|  |  |  |  |  |  | MOL007125 | neocryptotanshinone |
|  |  |  |  |  |  | MOL007127 | 1-methyl-8,9-dihydro-7H-naphtho[5,6-g]benzofuran-6,10,11-trione |
|  |  |  |  |  |  | MOL007130 | prolithospermic acid |
|  |  |  |  |  |  | MOL007140 | (Z)-3-[2-[(E)-2-(3,4-dihydroxyphenyl)vinyl]-3,4-dihydroxy-phenyl]acrylic acid |
|  |  |  |  |  |  | MOL007149 | NSC 122421 |
|  |  |  |  |  |  | MOL007150 | (6S)-6-hydroxy-1-methyl-6-methylol-8,9-dihydro-7H-naphtho[8,7-g]benzofuran-10,11-quinone |
|  |  |  |  |  |  | MOL007151 | Tanshindiol B |
|  |  |  |  |  |  | MOL007152 | Przewaquinone E |
|  |  |  |  |  |  | MOL007154 | tanshinone iia |
|  |  |  |  |  |  | MOL007155 | (6S)-6-(hydroxymethyl)-1,6-dimethyl-8,9-dihydro-7H-naphtho[8,7-g]benzofuran-10,11-dione |
|  |  |  |  |  |  | MOL007156 | tanshinone Ⅵ |
|  | Shanyao | Dioscorea oppositifolia L. | Dioscoreaceae | Dioscorea oppositifolia | Rhizome | MOL001559 | [piperlonguminine](https://www.tcmsp-e.com/molecule.php?qn=1559) |
|  |  |  |  |  |  | MOL001736 | [(-)-taxifolin](https://www.tcmsp-e.com/molecule.php?qn=1736) |
|  |  |  |  |  |  | MOL000310 | [Denudatin B](https://www.tcmsp-e.com/molecule.php?qn=310) |
|  |  |  |  |  |  | MOL000322 | [Kadsurenone](https://www.tcmsp-e.com/molecule.php?qn=322) |
|  |  |  |  |  |  | MOL005429 | [hancinol](https://www.tcmsp-e.com/molecule.php?qn=5429) |
|  |  |  |  |  |  | MOL005430 | [hancinone C](https://www.tcmsp-e.com/molecule.php?qn=5430) |
|  |  |  |  |  |  | MOL005435 | [24-Methylcholest-5-enyl-3belta-O-glucopyranoside_qt](https://www.tcmsp-e.com/molecule.php?qn=5435) |
|  |  |  |  |  |  | MOL005438 | [campesterol](https://www.tcmsp-e.com/molecule.php?qn=5438) |
|  |  |  |  |  |  | MOL005440 | [Isofucosterol](https://www.tcmsp-e.com/molecule.php?qn=5440) |
|  |  |  |  |  |  | MOL000449 | [Stigmasterol](https://www.tcmsp-e.com/molecule.php?qn=449) |
|  |  |  |  |  |  | MOL005458 | [Dioscoreside C_qt](https://www.tcmsp-e.com/molecule.php?qn=5458) |
|  |  |  |  |  |  | MOL000546 | [diosgenin](https://www.tcmsp-e.com/molecule.php?qn=546) |
|  |  |  |  |  |  | MOL005461 | [Doradexanthin](https://www.tcmsp-e.com/molecule.php?qn=5461) |
|  |  |  |  |  |  | MOL005463 | [Methylcimicifugoside_qt](https://www.tcmsp-e.com/molecule.php?qn=5463) |
|  |  |  |  |  |  | MOL005465 | [AIDS180907](https://www.tcmsp-e.com/molecule.php?qn=5465) |
|  |  |  |  |  |  | MOL000953 | [CLR](https://www.tcmsp-e.com/molecule.php?qn=953) |
|  | Dihuang | Rehmannia glutinosa (Gaertn.) Libosch. ex DC. | [Orobanchaceae](https://powo.science.kew.org/taxon/urn:lsid:ipni.org:names:30003446-2) | Rehmannia glutinosa | Root | MOL000359 | [sitosterol](https://www.tcmsp-e.com/molecule.php?qn=359) |
|  |  |  |  |  |  | MOL000449 | [Stigmasterol](https://www.tcmsp-e.com/molecule.php?qn=449) |
| Zuogui Pill | Dihuang | Rehmannia glutinosa (Gaertn.) Libosch. ex DC. | [Orobanchaceae](https://powo.science.kew.org/taxon/urn:lsid:ipni.org:names:30003446-2) | Rehmannia glutinosa | Root | MOL000359 | [sitosterol](https://www.tcmsp-e.com/molecule.php?qn=359) |
|  |  |  |  |  |  | MOL000449 | [Stigmasterol](https://www.tcmsp-e.com/molecule.php?qn=449) |
|  | Shanyao | Dioscorea oppositifolia L. | Dioscoreaceae | Dioscorea oppositifolia | Rhizome | MOL001559 | [piperlonguminine](https://www.tcmsp-e.com/molecule.php?qn=1559) |
|  |  |  |  |  |  | MOL001736 | [(-)-taxifolin](https://www.tcmsp-e.com/molecule.php?qn=1736) |
|  |  |  |  |  |  | MOL000310 | [Denudatin B](https://www.tcmsp-e.com/molecule.php?qn=310) |
|  |  |  |  |  |  | MOL000322 | [Kadsurenone](https://www.tcmsp-e.com/molecule.php?qn=322) |
|  |  |  |  |  |  | MOL005429 | [hancinol](https://www.tcmsp-e.com/molecule.php?qn=5429) |
|  |  |  |  |  |  | MOL005430 | [hancinone C](https://www.tcmsp-e.com/molecule.php?qn=5430) |
|  |  |  |  |  |  | MOL005435 | [24-Methylcholest-5-enyl-3belta-O-glucopyranoside_qt](https://www.tcmsp-e.com/molecule.php?qn=5435) |
|  |  |  |  |  |  | MOL005438 | [campesterol](https://www.tcmsp-e.com/molecule.php?qn=5438) |
|  |  |  |  |  |  | MOL005440 | [Isofucosterol](https://www.tcmsp-e.com/molecule.php?qn=5440) |
|  |  |  |  |  |  | MOL000449 | [Stigmasterol](https://www.tcmsp-e.com/molecule.php?qn=449) |
|  |  |  |  |  |  | MOL005458 | [Dioscoreside C_qt](https://www.tcmsp-e.com/molecule.php?qn=5458) |
|  |  |  |  |  |  | MOL000546 | [diosgenin](https://www.tcmsp-e.com/molecule.php?qn=546) |
|  |  |  |  |  |  | MOL005461 | [Doradexanthin](https://www.tcmsp-e.com/molecule.php?qn=5461) |
|  |  |  |  |  |  | MOL005463 | [Methylcimicifugoside_qt](https://www.tcmsp-e.com/molecule.php?qn=5463) |
|  |  |  |  |  |  | MOL005465 | [AIDS180907](https://www.tcmsp-e.com/molecule.php?qn=5465) |
|  |  |  |  |  |  | MOL000953 | [CLR](https://www.tcmsp-e.com/molecule.php?qn=953) |
|  | Gouqizi | Lycium barbarum L. | Solanaceae | Lycium barbarum | Dried ripe fruit | MOL001323 | Sitosterol alpha1 |
|  |  |  |  |  |  | MOL003578 | Cycloartenol |
|  |  |  |  |  |  | MOL001494 | Mandenol |
|  |  |  |  |  |  | MOL001495 | Ethyl linolenate |
|  |  |  |  |  |  | MOL001979 | LAN |
|  |  |  |  |  |  | MOL000449 | Stigmasterol |
|  |  |  |  |  |  | MOL000358 | beta-sitosterol |
|  |  |  |  |  |  | MOL005438 | campesterol |
|  |  |  |  |  |  | MOL007449 | 24-methylidenelophenol |
|  |  |  |  |  |  | MOL008173 | daucosterol_qt |
|  |  |  |  |  |  | MOL008400 | glycitein |
|  |  |  |  |  |  | MOL000953 | CLR |
|  |  |  |  |  |  | MOL009604 | 14b-pregnane |
|  |  |  |  |  |  | MOL009612 | (24R)-4alpha-Methyl-24-ethylcholesta-7,25-dien-3beta-ylacetate |
|  |  |  |  |  |  | MOL009615 | 24-Methylenecycloartan-3beta,21-diol |
|  |  |  |  |  |  | MOL009617 | 24-ethylcholest-22-enol |
|  |  |  |  |  |  | MOL009618 | 24-ethylcholesta-5,22-dienol |
|  |  |  |  |  |  | MOL009620 | 24-methyl-31-norlanost-9(11)-enol |
|  |  |  |  |  |  | MOL009621 | 24-methylenelanost-8-enol |
|  |  |  |  |  |  | MOL009622 | Fucosterol |
|  |  |  |  |  |  | MOL009631 | 31-Norcyclolaudenol |
|  |  |  |  |  |  | MOL009633 | 31-norlanost-9(11)-enol |
|  |  |  |  |  |  | MOL009634 | 31-norlanosterol |
|  |  |  |  |  |  | MOL009635 | 4,24-methyllophenol |
|  |  |  |  |  |  | MOL009639 | Lophenol |
|  |  |  |  |  |  | MOL009640 | 4alpha,14alpha,24-trimethylcholesta-8,24-dienol |
|  |  |  |  |  |  | MOL009641 | 4alpha,24-dimethylcholesta-7,24-dienol |
|  |  |  |  |  |  | MOL009642 | 4alpha-methyl-24-ethylcholesta-7,24-dienol |
|  |  |  |  |  |  | MOL009644 | 6-Fluoroindole-7-Dehydrocholesterol |
|  |  |  |  |  |  | MOL009646 | 7-O-Methylluteolin-6-C-beta-glucoside_qt |
|  |  |  |  |  |  | MOL009650 | Atropine |
|  |  |  |  |  |  | MOL009651 | Cryptoxanthin monoepoxide |
|  |  |  |  |  |  | MOL009653 | Cycloeucalenol |
|  |  |  |  |  |  | MOL009656 | (E,E)-1-ethyl octadeca-3,13-dienoate |
|  |  |  |  |  |  | MOL009662 | Lantadene A |
|  |  |  |  |  |  | MOL009677 | lanost-8-en-3beta-ol |
|  |  |  |  |  |  | MOL009678 | lanost-8-enol |
|  |  |  |  |  |  | MOL009681 | Obtusifoliol |
|  |  |  |  |  |  | MOL000098 | quercetin |
|  | Shanzhuyu | Cornus officinalis Siebold & Zucc. | Cornaceae | Cornus officinalis | Ripe fruit | MOL002883 | Ethyl oleate (NF) |
|  |  |  |  |  |  | MOL008457 | Tetrahydroalstonine |
|  |  |  |  |  |  | MOL005481 | 2,6,10,14,18-pentamethylicosa-2,6,10,14,18-pentaene |
|  |  |  |  |  |  | MOL005530 | Hydroxygenkwanin |
|  |  |  |  |  |  | MOL000359 | sitosterol |
|  |  |  |  |  |  | MOL001771 | poriferast-5-en-3beta-ol |
|  |  |  |  |  |  | MOL000358 | beta-sitosterol |
|  |  |  |  |  |  | MOL001494 | Mandenol |
|  |  |  |  |  |  | MOL000449 | Stigmasterol |
|  |  |  |  |  |  | MOL005557 | lanosta-8,24-dien-3-ol,3-acetate |
|  |  |  |  |  |  | MOL001495 | Ethyl linolenate |
|  |  |  |  |  |  | MOL005486 | 3,4-Dehydrolycopen-16-al |
|  |  |  |  |  |  | MOL005360 | malkangunin |
|  |  |  |  |  |  | MOL005531 | Telocinobufagin |
|  | Niuxi | Achyranthes bidentata Blume | Amaranthaceae | Achyranthes bidentata | Root | MOL012505 | bidentatoside,ii_qt |
|  |  |  |  |  |  | MOL000085 | beta-daucosterol_qt |
|  |  |  |  |  |  | MOL002643 | delta 7-stigmastenol |
|  |  |  |  |  |  | MOL004355 | Spinasterol |
|  |  |  |  |  |  | MOL000358 | beta-sitosterol |
|  |  |  |  |  |  | MOL001006 | poriferasta-7,22E-dien-3beta-ol |
|  |  |  |  |  |  | MOL000449 | Stigmasterol |
|  |  |  |  |  |  | MOL002897 | epiberberine |
|  |  |  |  |  |  | MOL001454 | berberine |
|  |  |  |  |  |  | MOL001458 | coptisine |
|  |  |  |  |  |  | MOL000098 | quercetin |
|  |  |  |  |  |  | MOL000422 | kaempferol |
|  |  |  |  |  |  | MOL003847 | Inophyllum E |
|  |  |  |  |  |  | MOL002714 | baicalein |
|  |  |  |  |  |  | MOL000173 | wogonin |
|  | Tusizi | Cuscuta chinensis Lam. | Convolvulaceae | Cuscuta chinensis | Seed | MOL001558 | [sesamin](https://www.tcmsp-e.com/molecule.php?qn=1558) |
|  |  |  |  |  |  | MOL000184 | [NSC63551](https://www.tcmsp-e.com/molecule.php?qn=184) |
|  |  |  |  |  |  | MOL000354 | [isorhamnetin](https://www.tcmsp-e.com/molecule.php?qn=354) |
|  |  |  |  |  |  | MOL000358 | [beta-sitosterol](https://www.tcmsp-e.com/molecule.php?qn=358) |
|  |  |  |  |  |  | MOL000422 | [kaempferol](https://www.tcmsp-e.com/molecule.php?qn=422) |
|  |  |  |  |  |  | MOL005043 | [campest-5-en-3beta-ol](https://www.tcmsp-e.com/molecule.php?qn=5043) |
|  |  |  |  |  |  | MOL005440 | [Isofucosterol](https://www.tcmsp-e.com/molecule.php?qn=5440) |
|  |  |  |  |  |  | MOL005944 | [matrine](https://www.tcmsp-e.com/molecule.php?qn=5944) |
|  |  |  |  |  |  | MOL006649 | [sophranol](https://www.tcmsp-e.com/molecule.php?qn=6649) |
|  |  |  |  |  |  | MOL000953 | [CLR](https://www.tcmsp-e.com/molecule.php?qn=953) |
|  |  |  |  |  |  | MOL000098 | [quercetin](https://www.tcmsp-e.com/molecule.php?qn=98) |
| Bajitianwan formula | Bajitian | Gynochthodes officinalis (F.C.How) Razafim. & B.Bremer | Rubiaceae | Gynochthodes officinalis | Root | MOL002883 | Ethyl oleate (NF) |
|  |  |  |  |  |  | MOL000358 | beta-sitosterol |
|  |  |  |  |  |  | MOL000359 | sitosterol |
|  |  |  |  |  |  | MOL006147 | Alizarin-2-methylether |
|  |  |  |  |  |  | MOL009495 | 2-hydroxy-1,5-dimethoxy-6-(methoxymethyl)-9,10-anthraquinone |
|  |  |  |  |  |  | MOL009496 | 1,5,7-trihydroxy-6-methoxy-2-methoxymethylanthracenequinone |
|  |  |  |  |  |  | MOL009500 | 1,6-dihydroxy-5-methoxy-2-(methoxymethyl)-9,10-anthraquinone |
|  |  |  |  |  |  | MOL009503 | 1-hydroxy-3-methoxy-9,10-anthraquinone |
|  |  |  |  |  |  | MOL009504 | 1-hydroxy-6-hydroxymethylanthracenequinone |
|  |  |  |  |  |  | MOL009513 | 2-hydroxy-1,8-dimethoxy-7-methoxymethylanthracenequinone |
|  |  |  |  |  |  | MOL009519 | (2R,3S)-(+)-3',5-Dihydroxy-4 ,7-dimethoxydihydroflavonol |
|  |  |  |  |  |  | MOL009524 | 3beta,20(R),5-alkenyl-stigmastol |
|  |  |  |  |  |  | MOL009525 | 3beta-24S(R)-butyl-5-alkenyl-cholestol |
|  |  |  |  |  |  | MOL009562 | Ohioensin-A |
|  | Shichangpu | Acorus verus (L.) Raf. | [Acoraceae](https://powo.science.kew.org/taxon/urn:lsid:ipni.org:names:77126753-1) | Acorus verus | Rhizome | MOL003542 | 8-Isopentenyl-kaempferol |
|  |  |  |  |  |  | MOL003578 | Cycloartenol |
|  |  |  |  |  |  | MOL000422 | kaempferol |
|  | Digupi | [Lycium chinense Mill.](https://mpns.science.kew.org/mpns-portal/plantDetail?plantId=2496344&query=Lycii+Cortex&filter=&fuzzy=false&nameType=all&dbs=wcsCmp) | Solanaceae | Lycium chinense | Root bark | MOL001552 | OIN |
|  |  |  |  |  |  | MOL001645 | Linoleyl acetate |
|  |  |  |  |  |  | MOL001689 | acacetin |
|  |  |  |  |  |  | MOL002222 | sugiol |
|  |  |  |  |  |  | MOL002224 | aurantiamide acetate |
|  |  |  |  |  |  | MOL002228 | Kulactone |
|  |  |  |  |  |  | MOL000296 | hederagenin |
|  |  |  |  |  |  | MOL000358 | beta-sitosterol |
|  |  |  |  |  |  | MOL000449 | Stigmasterol |
|  |  |  |  |  |  | MOL000953 | CLR |
|  | fuling | Ophrys apifera Huds. | [Orchidaceae](https://powo.science.kew.org/taxon/urn:lsid:ipni.org:names:30000046-2) | Ophrys apifera | Sclerotium | MOL000291 | Poricoic acid B |
|  |  |  |  |  |  | MOL000290 | Poricoic acid A |
|  |  |  |  |  |  | MOL000273 | (2R)-2-[(3S,5R,10S,13R,14R,16R,17R)-3,16-dihydroxy-4,4,10,13,14-pentamethyl-2,3,5,6,12,15,16,17-octahydro-1H-cyclopenta[a]phenanthren-17-yl]-6-methylhept-5-enoic acid |
|  |  |  |  |  |  | MOL000280 | (2R)-2-[(3S,5R,10S,13R,14R,16R,17R)-3,16-dihydroxy-4,4,10,13,14-pentamethyl-2,3,5,6,12,15,16,17-octahydro-1H-cyclopenta[a]phenanthren-17-yl]-5-isopropyl-hex-5-enoic acid |
|  |  |  |  |  |  | MOL000289 | pachymic acid |
|  |  |  |  |  |  | MOL000276 | 7,9(11)-dehydropachymic acid |
|  |  |  |  |  |  | MOL000296 | hederagenin |
|  |  |  |  |  |  | MOL000279 | Cerevisterol |
|  |  |  |  |  |  | MOL000292 | poricoic acid C |
|  |  |  |  |  |  | MOL000285 | (2R)-2-[(5R,10S,13R,14R,16R,17R)-16-hydroxy-3-keto-4,4,10,13,14-pentamethyl-1,2,5,6,12,15,16,17-octahydrocyclopenta[a]phenanthren-17-yl]-5-isopropyl-hex-5-enoic acid |
|  |  |  |  |  |  | MOL000287 | 3beta-Hydroxy-24-methylene-8-lanostene-21-oic acid |
|  |  |  |  |  |  | MOL000275 | trametenolic acid |
|  |  |  |  |  |  | MOL000282 | ergosta-7,22E-dien-3beta-ol |
|  |  |  |  |  |  | MOL000300 | dehydroeburicoic acid |
|  | Yuanzhi | Polygala tenuifolia Willd. | Polygalaceae | Polygala tenuifolia | Root | MOL013171 | 1,6-Dihydroxy-3,7-dimethoxyxanthone |
|  |  |  |  |  |  | MOL008405 | 1-Peroxyferolide |
|  |  |  |  |  |  | MOL001997 | 5,6,7-Trimethoxycoumarin |
|  |  |  |  |  |  | MOL004718 | α-spinasterol |
|  |  |  |  |  |  | MOL000124 | citral |
|  |  |  |  |  |  | MOL006841 | Harman |
|  |  |  |  |  |  | MOL004368 | hyperin |
|  |  |  |  |  |  | MOL002051 | isoquercitrin |
|  |  |  |  |  |  | MOL001604 | linalool |
|  |  |  |  |  |  | MOL004358 | linalool |
|  |  |  |  |  |  | MOL006844 | Norharman |
|  |  |  |  |  |  | MOL011525 | Norhyoscyamine |
|  |  |  |  |  |  | MOL009996 | Onjisaponin F |
|  |  |  |  |  |  | MOL003369 | Onjisaponin G |
|  |  |  |  |  |  | MOL003370 | Onjixanthone I |
|  |  |  |  |  |  | MOL002140 | Perlolyrine |
|  |  |  |  |  |  | MOL000701 | quercitrin |
|  |  |  |  |  |  | MOL000415 | rutin |
|  |  |  |  |  |  | MOL000842 | sucrose |
|  |  |  |  |  |  | MOL002042 | thymol |
|  | Renshen | Panax ginseng C.A.Mey. | Araliaceae | Panax ginseng | Root | MOL000449 | [Stigmasterol](https://www.tcmsp-e.com/molecule.php?qn=449) |
|  |  |  |  |  |  | MOL000358 | [beta-sitosterol](https://www.tcmsp-e.com/molecule.php?qn=358) |
|  |  |  |  |  |  | MOL003648 | [Inermin](https://www.tcmsp-e.com/molecule.php?qn=3648) |
|  |  |  |  |  |  | MOL000422 | [kaempferol](https://www.tcmsp-e.com/molecule.php?qn=422) |
|  |  |  |  |  |  | MOL004492 | [Chrysanthemaxanthin](https://www.tcmsp-e.com/molecule.php?qn=4492) |
|  |  |  |  |  |  | MOL005314 | [Celabenzine](https://www.tcmsp-e.com/molecule.php?qn=5314) |
|  |  |  |  |  |  | MOL005317 | [Deoxyharringtonine](https://www.tcmsp-e.com/molecule.php?qn=5317) |
|  |  |  |  |  |  | MOL005318 | [Dianthramine](https://www.tcmsp-e.com/molecule.php?qn=5318) |
|  |  |  |  |  |  | MOL005320 | [arachidonate](https://www.tcmsp-e.com/molecule.php?qn=5320) |
|  |  |  |  |  |  | MOL005321 | [Frutinone A](https://www.tcmsp-e.com/molecule.php?qn=5321) |
|  |  |  |  |  |  | MOL005348 | [Ginsenoside-Rh4_qt](https://www.tcmsp-e.com/molecule.php?qn=5348) |
|  |  |  |  |  |  | MOL005356 | [Girinimbin](https://www.tcmsp-e.com/molecule.php?qn=5356) |
|  |  |  |  |  |  | MOL005357 | [Gomisin B](https://www.tcmsp-e.com/molecule.php?qn=5357) |
|  |  |  |  |  |  | MOL005360 | [malkangunin](https://www.tcmsp-e.com/molecule.php?qn=5360) |
|  |  |  |  |  |  | MOL005376 | [Panaxadiol](https://www.tcmsp-e.com/molecule.php?qn=5376) |
|  |  |  |  |  |  | MOL005384 | [suchilactone](https://www.tcmsp-e.com/molecule.php?qn=5384) |
|  |  |  |  |  |  | MOL005399 | [alexandrin_qt](https://www.tcmsp-e.com/molecule.php?qn=5399) |
|  |  |  |  |  |  | MOL005401 | [ginsenoside Rg5_qt](https://www.tcmsp-e.com/molecule.php?qn=5401) |
|  |  |  |  |  |  | MOL000787 | [Fumarine](https://www.tcmsp-e.com/molecule.php?qn=787) |
| Heng-Gu-Gu-Shang-Yu-He-Ji | Renshen | Panax ginseng C.A.Mey. | Araliaceae | Panax ginseng | Root | MOL000449 | [Stigmasterol](https://www.tcmsp-e.com/molecule.php?qn=449) |
|  |  |  |  |  |  | MOL000358 | [beta-sitosterol](https://www.tcmsp-e.com/molecule.php?qn=358) |
|  |  |  |  |  |  | MOL003648 | [Inermin](https://www.tcmsp-e.com/molecule.php?qn=3648) |
|  |  |  |  |  |  | MOL000422 | [kaempferol](https://www.tcmsp-e.com/molecule.php?qn=422) |
|  |  |  |  |  |  | MOL004492 | [Chrysanthemaxanthin](https://www.tcmsp-e.com/molecule.php?qn=4492) |
|  |  |  |  |  |  | MOL005314 | [Celabenzine](https://www.tcmsp-e.com/molecule.php?qn=5314) |
|  |  |  |  |  |  | MOL005317 | [Deoxyharringtonine](https://www.tcmsp-e.com/molecule.php?qn=5317) |
|  |  |  |  |  |  | MOL005318 | [Dianthramine](https://www.tcmsp-e.com/molecule.php?qn=5318) |
|  |  |  |  |  |  | MOL005320 | [arachidonate](https://www.tcmsp-e.com/molecule.php?qn=5320) |
|  |  |  |  |  |  | MOL005321 | [Frutinone A](https://www.tcmsp-e.com/molecule.php?qn=5321) |
|  |  |  |  |  |  | MOL005348 | [Ginsenoside-Rh4_qt](https://www.tcmsp-e.com/molecule.php?qn=5348) |
|  |  |  |  |  |  | MOL005356 | [Girinimbin](https://www.tcmsp-e.com/molecule.php?qn=5356) |
|  |  |  |  |  |  | MOL005357 | [Gomisin B](https://www.tcmsp-e.com/molecule.php?qn=5357) |
|  |  |  |  |  |  | MOL005360 | [malkangunin](https://www.tcmsp-e.com/molecule.php?qn=5360) |
|  |  |  |  |  |  | MOL005376 | [Panaxadiol](https://www.tcmsp-e.com/molecule.php?qn=5376) |
|  |  |  |  |  |  | MOL005384 | [suchilactone](https://www.tcmsp-e.com/molecule.php?qn=5384) |
|  |  |  |  |  |  | MOL005399 | [alexandrin_qt](https://www.tcmsp-e.com/molecule.php?qn=5399) |
|  |  |  |  |  |  | MOL005401 | [ginsenoside Rg5_qt](https://www.tcmsp-e.com/molecule.php?qn=5401) |
|  |  |  |  |  |  | MOL000787 | [Fumarine](https://www.tcmsp-e.com/molecule.php?qn=787) |
|  | Honghua | Carthamus tinctorius L. | Asteraceae | Carthamus tinctorius | Flower | MOL001771 | poriferast-5-en-3beta-ol |
|  |  |  |  |  |  | MOL002680 | Flavoxanthin |
|  |  |  |  |  |  | MOL002695 | lignan |
|  |  |  |  |  |  | MOL002698 | lupeol-palmitate |
|  |  |  |  |  |  | MOL002712 | 6-Hydroxykaempferol |
|  |  |  |  |  |  | MOL002714 | baicalein |
|  |  |  |  |  |  | MOL002719 | 6-Hydroxynaringenin |
|  |  |  |  |  |  | MOL002721 | quercetagetin |
|  |  |  |  |  |  | MOL002773 | beta-carotene |
|  |  |  |  |  |  | MOL000358 | beta-sitosterol |
|  |  |  |  |  |  | MOL000422 | kaempferol |
|  |  |  |  |  |  | MOL000449 | Stigmasterol |
|  |  |  |  |  |  | MOL000006 | luteolin |
|  |  |  |  |  |  | MOL000953 | CLR |
|  |  |  |  |  |  | MOL000098 | quercetin |
|  | Sanqi | Panax notoginseng (Burkill) F.H.Chen | [Araliaceae](https://powo.science.kew.org/taxon/urn:lsid:ipni.org:names:30001539-2) | Panax notoginseng | Root and Rhizome | MOL001494 | [Mandenol](https://www.tcmsp-e.com/molecule.php?qn=1494) |
|  |  |  |  |  |  | MOL001792 | [DFV](https://www.tcmsp-e.com/molecule.php?qn=1792) |
|  |  |  |  |  |  | MOL000358 | [beta-sitosterol](https://www.tcmsp-e.com/molecule.php?qn=358) |
|  |  |  |  |  |  | MOL000449 | [Stigmasterol](https://www.tcmsp-e.com/molecule.php?qn=449) |
|  |  |  |  |  |  | MOL000098 | [quercetin](https://www.tcmsp-e.com/molecule.php?qn=98) |
|  | Huangqi | [Astragalus mongholicus Bunge](https://mpns.science.kew.org/mpns-portal/plantDetail?plantId=2661222&query=Astragalus+membranaceus+%28Fisch.%29Bge.&filter=&fuzzy=false&nameType=all&dbs=wcs) | [Fabaceae](https://powo.science.kew.org/taxon/urn:lsid:ipni.org:names:30000147-2) | Astragalus mongholicus | Root | MOL000211 | Mairin |
|  |  |  |  |  |  | MOL000239 | Jaranol |
|  |  |  |  |  |  | MOL000296 | hederagenin |
|  |  |  |  |  |  | MOL000033 | (3S,8S,9S,10R,13R,14S,17R)-10,13-dimethyl-17-[(2R,5S)-5-propan-2-yloctan-2-yl]-2,3,4,7,8,9,11,12,14,15,16,17-dodecahydro-1H-cyclopenta[a]phenanthren-3-ol |
|  |  |  |  |  |  | MOL000354 | isorhamnetin |
|  |  |  |  |  |  | MOL000371 | 3,9-di-O-methylnissolin |
|  |  |  |  |  |  | MOL000380 | (6aR,11aR)-9,10-dimethoxy-6a,11a-dihydro-6H-benzofurano[3,2-c]chromen-3-ol |
|  |  |  |  |  |  | MOL000387 | Bifendate |
|  |  |  |  |  |  | MOL000392 | formononetin |
|  |  |  |  |  |  | MOL000398 | isoflavanone |
|  |  |  |  |  |  | MOL000417 | Calycosin |
|  |  |  |  |  |  | MOL000422 | kaempferol |
|  |  |  |  |  |  | MOL000442 | 1,7-Dihydroxy-3,9-dimethoxy pterocarpene |
|  |  |  |  |  |  | MOL000098 | quercetin |
|  | Yangjinhua | Datura metel L | Solanaceae | Datura metel | Flower | MOL001554 | Scopolamine |
|  |  |  |  |  |  | MOL011470 | 12-Deoxywithastramonolide |
|  |  |  |  |  |  | MOL011487 | Datuarmeteloside B_qt |
|  |  |  |  |  |  | MOL011490 | Datumetelin |
|  |  |  |  |  |  | MOL011495 | Daturametelin A_qt |
|  |  |  |  |  |  | MOL011497 | (6R)-6-[(1R)-2-hydroxy-1-[(8S,9S,10R,13S,14S,17R)-1-keto-10,13-dimethyl-4,7,8,9,11,12,14,15,16,17-decahydrocyclopenta[a]phenanthren-17-yl]ethyl]-4-methyl-3-methylol-5,6-dihydropyran-2-one |
|  |  |  |  |  |  | MOL011498 | (1R,2R,5R,6S)-2-[(8S,9S,10R,13S,14S,17R)-10,13-dimethyl-1-oxo-4,7,8,9,11,12,14,15,16,17-decahydrocyclopenta[a]phenanthren-17-yl]-6-(methoxymethyl)-5-methyl-4,8-dioxabicyclo[3.3.1]nonan-7-one |
|  |  |  |  |  |  | MOL011520 | hypaconitine |
|  |  |  |  |  |  | MOL011531 | Secowithamerclin |
|  |  |  |  |  |  | MOL011539 | Withametelin |
|  |  |  |  |  |  | MOL011540 | withanolide D |
|  |  |  |  |  |  | MOL003644 | Withaferine |
|  |  |  |  |  |  | MOL000422 | kaempferol |
|  |  |  |  |  |  | MOL000631 | coumaroyltyramine |
|  |  |  |  |  |  | MOL007923 | 2-(4-hydroxyphenyl)ethyl (E)-3-(4-hydroxyphenyl)prop-2-enoate |
|  |  |  |  |  |  | MOL000098 | quercetin |
|  | Duzhong | Eucommia ulmoides Oliv. | Eucommiaceae | Eucommia ulmoides | Bark | MOL000211 | [Mairin](https://www.tcmsp-e.com/molecule.php?qn=211) |
|  |  |  |  |  |  | MOL000358 | [beta-sitosterol](https://www.tcmsp-e.com/molecule.php?qn=358) |
|  |  |  |  |  |  | MOL000422 | [kaempferol](https://www.tcmsp-e.com/molecule.php?qn=422) |
|  |  |  |  |  |  | MOL000443 | [Erythraline](https://www.tcmsp-e.com/molecule.php?qn=443) |
|  |  |  |  |  |  | MOL007059 | [3-beta-Hydroxymethyllenetanshiquinone](https://www.tcmsp-e.com/molecule.php?qn=7059) |
|  |  |  |  |  |  | MOL009015 | [(-)-Tabernemontanine](https://www.tcmsp-e.com/molecule.php?qn=9015) |
|  |  |  |  |  |  | MOL009027 | [Cyclopamine](https://www.tcmsp-e.com/molecule.php?qn=9027) |
|  |  |  |  |  |  | MOL009029 | [Dehydrodiconiferyl alcohol 4,gamma'-di-O-beta-D-glucopyanoside_qt](https://www.tcmsp-e.com/molecule.php?qn=9029) |
|  |  |  |  |  |  | MOL009042 | [Helenalin](https://www.tcmsp-e.com/molecule.php?qn=9042) |
|  |  |  |  |  |  | MOL009053 | [4-[(2S,3R)-5-[(E)-3-hydroxyprop-1-enyl]-7-methoxy-3-methylol-2,3-dihydrobenzofuran-2-yl]-2-methoxy-phenol](https://www.tcmsp-e.com/molecule.php?qn=9053) |
|  |  |  |  |  |  | MOL000098 | [quercetin](https://www.tcmsp-e.com/molecule.php?qn=98) |
|  |  |  |  |  |  | MOL002773 | [beta-carotene](https://www.tcmsp-e.com/molecule.php?qn=2773) |
|  |  |  |  |  |  | MOL008240 | [(E)-3-[4-[(1R,2R)-2-hydroxy-2-(4-hydroxy-3-methoxy-phenyl)-1-methylol-ethoxy]-3-methoxy-phenyl]acrolein](https://www.tcmsp-e.com/molecule.php?qn=8240) |
|  |  |  |  |  |  | MOL011604 | Syringetin |
|  | Chenpi | Citrus reticulata Blanco | [Rutaceae](https://powo.science.kew.org/taxon/urn:lsid:ipni.org:names:30001492-2) | Citrus reticulata | Dried pericarp | MOL000359 | [sitosterol](https://www.tcmsp-e.com/molecule.php?qn=359) |
|  |  |  |  |  |  | MOL004328 | [naringenin](https://www.tcmsp-e.com/molecule.php?qn=4328) |
|  |  |  |  |  |  | MOL005100 | [5,7-dihydroxy-2-(3-hydroxy-4-methoxyphenyl)chroman-4-one](https://www.tcmsp-e.com/molecule.php?qn=5100) |
|  |  |  |  |  |  | MOL005815 | [Citromitin](https://www.tcmsp-e.com/molecule.php?qn=5815) |
|  |  |  |  |  |  | MOL005828 | [nobiletin](https://www.tcmsp-e.com/molecule.php?qn=5828) |
|  | Biejia | Trionycis Carapax | Trionychidae | Trionyx sinensis | Turtle shell | MOL010417 | 8,11-OCTADECADIENOIC ACID |
|  |  |  |  |  |  | MOL006944 | 8-Octadecenoic acid |
|  |  |  |  |  |  | MOL002052 | 9-Octadecenoic acid |
| Bushen Huoxue decoction | Dihuang | Rehmannia glutinosa (Gaertn.) Libosch. ex DC. | [Orobanchaceae](https://powo.science.kew.org/taxon/urn:lsid:ipni.org:names:30003446-2) | Rehmannia glutinosa | Root | MOL000359 | [sitosterol](https://www.tcmsp-e.com/molecule.php?qn=359) |
|  |  |  |  |  |  | MOL000449 | [Stigmasterol](https://www.tcmsp-e.com/molecule.php?qn=449) |
|  | Buguzhi | Cullen corylifolium (L.) Medik. | Fabaceae | Cullen corylifolium | Dried ripe fruit | MOL003590 | angelicin |
|  |  |  |  |  |  | MOL005009 | Corylifolinin |
|  |  |  |  |  |  | MOL000093 | daucosterol |
|  |  |  |  |  |  | MOL001525 | daucosterol |
|  |  |  |  |  |  | MOL002296 | daucosterol |
|  |  |  |  |  |  | MOL005868 | daucosterol |
|  |  |  |  |  |  | MOL007096 | daucosterol |
|  |  |  |  |  |  | MOL008172 | daucosterol |
|  |  |  |  |  |  | MOL008926 | daucosterol |
|  |  |  |  |  |  | MOL010584 | daucosterol |
|  |  |  |  |  |  | MOL012237 | daucosterol |
|  |  |  |  |  |  | MOL000448 | isobavachin |
|  |  |  |  |  |  | MOL001393 | myristic acid |
|  |  |  |  |  |  | MOL005639 | Neobavachalcone |
|  |  |  |  |  |  | MOL001950 | psoralen |
|  |  |  |  |  |  | MOL000860 | stearic acid |
|  |  |  |  |  |  | MOL000449 | stigmasterol |
|  |  |  |  |  |  | MOL002045 | stigmasterol |
|  |  |  |  |  |  | MOL008159 | TRIACONTANE |
|  | Tusizi | Cuscuta chinensis Lam. | Convolvulaceae | Cuscuta chinensis | Seed | MOL001558 | [sesamin](https://www.tcmsp-e.com/molecule.php?qn=1558) |
|  |  |  |  |  |  | MOL000184 | [NSC63551](https://www.tcmsp-e.com/molecule.php?qn=184) |
|  |  |  |  |  |  | MOL000354 | [isorhamnetin](https://www.tcmsp-e.com/molecule.php?qn=354) |
|  |  |  |  |  |  | MOL000358 | [beta-sitosterol](https://www.tcmsp-e.com/molecule.php?qn=358) |
|  |  |  |  |  |  | MOL000422 | [kaempferol](https://www.tcmsp-e.com/molecule.php?qn=422) |
|  |  |  |  |  |  | MOL005043 | [campest-5-en-3beta-ol](https://www.tcmsp-e.com/molecule.php?qn=5043) |
|  |  |  |  |  |  | MOL005440 | [Isofucosterol](https://www.tcmsp-e.com/molecule.php?qn=5440) |
|  |  |  |  |  |  | MOL005944 | [matrine](https://www.tcmsp-e.com/molecule.php?qn=5944) |
|  |  |  |  |  |  | MOL006649 | [sophranol](https://www.tcmsp-e.com/molecule.php?qn=6649) |
|  |  |  |  |  |  | MOL000953 | [CLR](https://www.tcmsp-e.com/molecule.php?qn=953) |
|  |  |  |  |  |  | MOL000098 | [quercetin](https://www.tcmsp-e.com/molecule.php?qn=98) |
|  | Duzhong | Eucommia ulmoides Oliv. | Eucommiaceae | Eucommia ulmoides | Bark | MOL000211 | [Mairin](https://www.tcmsp-e.com/molecule.php?qn=211) |
|  |  |  |  |  |  | MOL000358 | [beta-sitosterol](https://www.tcmsp-e.com/molecule.php?qn=358) |
|  |  |  |  |  |  | MOL000422 | [kaempferol](https://www.tcmsp-e.com/molecule.php?qn=422) |
|  |  |  |  |  |  | MOL000443 | [Erythraline](https://www.tcmsp-e.com/molecule.php?qn=443) |
|  |  |  |  |  |  | MOL007059 | [3-beta-Hydroxymethyllenetanshiquinone](https://www.tcmsp-e.com/molecule.php?qn=7059) |
|  |  |  |  |  |  | MOL009015 | [(-)-Tabernemontanine](https://www.tcmsp-e.com/molecule.php?qn=9015) |
|  |  |  |  |  |  | MOL009027 | [Cyclopamine](https://www.tcmsp-e.com/molecule.php?qn=9027) |
|  |  |  |  |  |  | MOL009029 | [Dehydrodiconiferyl alcohol 4,gamma'-di-O-beta-D-glucopyanoside_qt](https://www.tcmsp-e.com/molecule.php?qn=9029) |
|  |  |  |  |  |  | MOL009042 | [Helenalin](https://www.tcmsp-e.com/molecule.php?qn=9042) |
|  |  |  |  |  |  | MOL009053 | [4-[(2S,3R)-5-[(E)-3-hydroxyprop-1-enyl]-7-methoxy-3-methylol-2,3-dihydrobenzofuran-2-yl]-2-methoxy-phenol](https://www.tcmsp-e.com/molecule.php?qn=9053) |
|  |  |  |  |  |  | MOL000098 | [quercetin](https://www.tcmsp-e.com/molecule.php?qn=98) |
|  |  |  |  |  |  | MOL002773 | [beta-carotene](https://www.tcmsp-e.com/molecule.php?qn=2773) |
|  |  |  |  |  |  | MOL008240 | [(E)-3-[4-[(1R,2R)-2-hydroxy-2-(4-hydroxy-3-methoxy-phenyl)-1-methylol-ethoxy]-3-methoxy-phenyl]acrolein](https://www.tcmsp-e.com/molecule.php?qn=8240) |
|  |  |  |  |  |  | MOL011604 | [Syringetin](https://www.tcmsp-e.com/molecule.php?qn=11604) |
|  | Gouqizi | Lycium barbarum L. | Solanaceae | Lycium barbarum | Dried ripe fruit | MOL001323 | Sitosterol alpha1 |
|  |  |  |  |  |  | MOL003578 | Cycloartenol |
|  |  |  |  |  |  | MOL001494 | Mandenol |
|  |  |  |  |  |  | MOL001495 | Ethyl linolenate |
|  |  |  |  |  |  | MOL001979 | LAN |
|  |  |  |  |  |  | MOL000449 | Stigmasterol |
|  |  |  |  |  |  | MOL000358 | beta-sitosterol |
|  |  |  |  |  |  | MOL005438 | campesterol |
|  |  |  |  |  |  | MOL007449 | 24-methylidenelophenol |
|  |  |  |  |  |  | MOL008173 | daucosterol_qt |
|  |  |  |  |  |  | MOL008400 | glycitein |
|  |  |  |  |  |  | MOL000953 | CLR |
|  |  |  |  |  |  | MOL009604 | 14b-pregnane |
|  |  |  |  |  |  | MOL009612 | (24R)-4alpha-Methyl-24-ethylcholesta-7,25-dien-3beta-ylacetate |
|  |  |  |  |  |  | MOL009615 | 24-Methylenecycloartan-3beta,21-diol |
|  |  |  |  |  |  | MOL009617 | 24-ethylcholest-22-enol |
|  |  |  |  |  |  | MOL009618 | 24-ethylcholesta-5,22-dienol |
|  |  |  |  |  |  | MOL009620 | 24-methyl-31-norlanost-9(11)-enol |
|  |  |  |  |  |  | MOL009621 | 24-methylenelanost-8-enol |
|  |  |  |  |  |  | MOL009622 | Fucosterol |
|  |  |  |  |  |  | MOL009631 | 31-Norcyclolaudenol |
|  |  |  |  |  |  | MOL009633 | 31-norlanost-9(11)-enol |
|  |  |  |  |  |  | MOL009634 | 31-norlanosterol |
|  |  |  |  |  |  | MOL009635 | 4,24-methyllophenol |
|  |  |  |  |  |  | MOL009639 | Lophenol |
|  |  |  |  |  |  | MOL009640 | 4alpha,14alpha,24-trimethylcholesta-8,24-dienol |
|  |  |  |  |  |  | MOL009641 | 4alpha,24-dimethylcholesta-7,24-dienol |
|  |  |  |  |  |  | MOL009642 | 4alpha-methyl-24-ethylcholesta-7,24-dienol |
|  |  |  |  |  |  | MOL009644 | 6-Fluoroindole-7-Dehydrocholesterol |
|  |  |  |  |  |  | MOL009646 | 7-O-Methylluteolin-6-C-beta-glucoside_qt |
|  |  |  |  |  |  | MOL009650 | Atropine |
|  |  |  |  |  |  | MOL009651 | Cryptoxanthin monoepoxide |
|  |  |  |  |  |  | MOL009653 | Cycloeucalenol |
|  |  |  |  |  |  | MOL009656 | (E,E)-1-ethyl octadeca-3,13-dienoate |
|  |  |  |  |  |  | MOL009662 | Lantadene A |
|  |  |  |  |  |  | MOL009677 | lanost-8-en-3beta-ol |
|  |  |  |  |  |  | MOL009678 | lanost-8-enol |
|  |  |  |  |  |  | MOL009681 | Obtusifoliol |
|  |  |  |  |  |  | MOL000098 | quercetin |
|  | Danggui | Angelica sinensis (Oliv.) Diels | Apiaceae | Angelica sinensis | Root | MOL000358 | [beta-sitosterol](https://www.tcmsp-e.com/molecule.php?qn=358) |
|  |  |  |  |  |  | MOL000449 | [Stigmasterol](https://www.tcmsp-e.com/molecule.php?qn=449) |
|  | Shanzhuyu | Cornus officinalis Siebold & Zucc. | Cornaceae | Cornus officinalis | Ripe fruit | MOL002883 | Ethyl oleate (NF) |
|  |  |  |  |  |  | MOL008457 | Tetrahydroalstonine |
|  |  |  |  |  |  | MOL005481 | 2,6,10,14,18-pentamethylicosa-2,6,10,14,18-pentaene |
|  |  |  |  |  |  | MOL005530 | Hydroxygenkwanin |
|  |  |  |  |  |  | MOL000359 | sitosterol |
|  |  |  |  |  |  | MOL001771 | poriferast-5-en-3beta-ol |
|  |  |  |  |  |  | MOL000358 | beta-sitosterol |
|  |  |  |  |  |  | MOL001494 | Mandenol |
|  |  |  |  |  |  | MOL000449 | Stigmasterol |
|  |  |  |  |  |  | MOL005557 | lanosta-8,24-dien-3-ol,3-acetate |
|  |  |  |  |  |  | MOL001495 | Ethyl linolenate |
|  |  |  |  |  |  | MOL005486 | 3,4-Dehydrolycopen-16-al |
|  |  |  |  |  |  | MOL005360 | malkangunin |
|  |  |  |  |  |  | MOL005531 | Telocinobufagin |
|  | Roucongrong | Cistanche deserticola Ma | Orobanchaceae | Cistanche deserticola | Stem | MOL000358 | beta-sitosterol |
|  |  |  |  |  |  | MOL005320 | [arachidonate](https://www.tcmsp-e.com/molecule.php?qn=5320) |
|  |  |  |  |  |  | MOL005384 | [suchilactone](https://www.tcmsp-e.com/molecule.php?qn=5384) |
|  |  |  |  |  |  | MOL000098 | [quercetin](https://www.tcmsp-e.com/molecule.php?qn=98) |
|  |  |  |  |  |  | MOL008871 | [Marckine](https://www.tcmsp-e.com/molecule.php?qn=8871) |
|  | Duhuo | Angelica biserrata (R.H.Shan & C.Q.Yuan) C.Q.Yuan & R.H.Shan | [Apiaceae](https://powo.science.kew.org/taxon/urn:lsid:ipni.org:names:30000180-2) | Angelica biserrata | Root | MOL000358 | [beta-sitosterol](https://www.tcmsp-e.com/molecule.php?qn=358) |
|  |  |  |  |  |  | MOL004780 | [Angelicone](https://www.tcmsp-e.com/molecule.php?qn=4780) |
|  | Honghua | Carthamus tinctorius L. | Asteraceae | Carthamus tinctorius | Flower | MOL001771 | poriferast-5-en-3beta-ol |
|  |  |  |  |  |  | MOL002680 | Flavoxanthin |
|  |  |  |  |  |  | MOL002695 | lignan |
|  |  |  |  |  |  | MOL002698 | lupeol-palmitate |
|  |  |  |  |  |  | MOL002712 | 6-Hydroxykaempferol |
|  |  |  |  |  |  | MOL002714 | baicalein |
|  |  |  |  |  |  | MOL002719 | 6-Hydroxynaringenin |
|  |  |  |  |  |  | MOL002721 | quercetagetin |
|  |  |  |  |  |  | MOL002773 | beta-carotene |
|  |  |  |  |  |  | MOL000358 | beta-sitosterol |
|  |  |  |  |  |  | MOL000422 | kaempferol |
|  |  |  |  |  |  | MOL000449 | Stigmasterol |
|  |  |  |  |  |  | MOL000006 | luteolin |
|  |  |  |  |  |  | MOL000953 | CLR |
|  |  |  |  |  |  | MOL000098 | quercetin |
|  | Moyao | Commiphora myrrha (T.Nees) Engl. | Burseraceae | Commiphora myrrha | Resin | MOL001006 | poriferasta-7,22E-dien-3beta-ol |
|  |  |  |  |  |  | MOL001013 | mansumbinoic acid |
|  |  |  |  |  |  | MOL001019 | (7S,8R,9S,10R,13S,14S,17Z)-17-ethylidene-7-hydroxy-10,13-dimethyl-1,2,6,7,8,9,11,12,14,15-decahydrocyclopenta[a]phenanthrene-3,16-dione |
|  |  |  |  |  |  | MOL001022 | 11α-hydroxypregna-4,17(20)-trans-diene-3,16-dione |
|  |  |  |  |  |  | MOL001031 | epimansumbinol |
|  |  |  |  |  |  | MOL001040 | (2R)-5,7-dihydroxy-2-(4-hydroxyphenyl)chroman-4-one |
|  |  |  |  |  |  | MOL001049 | 16-hydroperoxymansumbin-13(17)-en-3β-ol |
|  |  |  |  |  |  | MOL001052 | mansumbin-13(17)-en- 3,16-dione |
|  |  |  |  |  |  | MOL001061 | (16S, 20R)-dihydroxydammar-24-en-3-one |
|  |  |  |  |  |  | MOL001062 | 15α-hydroxymansumbinone |
|  |  |  |  |  |  | MOL001063 | 28-acetoxy-15α-hydroxymansumbinone |
|  |  |  |  |  |  | MOL001069 | 3β-acetoxy-16β,20(R)-dihydroxydammar-24-ene |
|  |  |  |  |  |  | MOL001088 | 1α-acetoxy-9,19-cyclolanost-24-en-3β-ol |
|  |  |  |  |  |  | MOL001092 | [(3R,5R,8R,9R,10R,13R,14R,17S)-17-[(2S,5S)-5-(2-hydroxypropan-2-yl)-2-methyloxolan-2-yl]-4,4,8,10,14-pentamethyl-2,3,5,6,7,9,11,12,13,15,16,17-dodecahydro-1H-cyclopenta[a]phenanthren-3-yl] acetate |
|  |  |  |  |  |  | MOL001093 | cabraleone |
|  |  |  |  |  |  | MOL001095 | isofouquierone |
|  |  |  |  |  |  | MOL001126 | [(5aS,8aR,9R)-8-oxo-9-(3,4,5-trimethoxyphenyl)-5,5a,6,9-tetrahydroisobenzofurano[6,5-f][1,3]benzodioxol-8a-yl] acetate |
|  |  |  |  |  |  | MOL001131 | phellamurin_qt |
|  |  |  |  |  |  | MOL001138 | (3R,20S)-3,20-dihydroxydammar- 24-ene |
|  |  |  |  |  |  | MOL001145 | (20S)-3β-acetoxy-12β,16β,25-tetrahydroxydammar-23-ene |
|  |  |  |  |  |  | MOL001146 | (20S)-3β,12β,16β,25-pentahydroxydammar-23-ene |
|  |  |  |  |  |  | MOL001147 | (20R)-3β-acetoxy-16β-dihydroxydammar-24-ene |
|  |  |  |  |  |  | MOL001148 | 3β- hydroxydammar-24-ene |
|  |  |  |  |  |  | MOL001156 | 3-methoxyfuranoguaia-9- en-8-one |
|  |  |  |  |  |  | MOL001164 | [(5S,6R,8R,9Z)-8-methoxy-3,6,10-trimethyl-4-oxo-6,7,8,11-tetrahydro-5H-cyclodeca[b]furan-5-yl] acetate |
|  |  |  |  |  |  | MOL000358 | beta-sitosterol |
|  |  |  |  |  |  | MOL000449 | Stigmasterol |
|  |  |  |  |  |  | MOL000979 | 2-methoxyfuranoguaia-9-ene-8-one |
|  |  |  |  |  |  | MOL000098 | quercetin |
|  |  |  |  |  |  | MOL000988 | 4,17(20)-(cis)-pregnadiene-3,16-dione |
|  |  |  |  |  |  | MOL000996 | Guggulsterol IV |
| Bushen Jianpi Huoxue Formula | Buguzhi | Cullen corylifolium (L.) Medik. | Fabaceae | Cullen corylifolium | Dried ripe fruit | MOL003590 | angelicin |
|  |  |  |  |  |  | MOL005009 | Corylifolinin |
|  |  |  |  |  |  | MOL000093 | daucosterol |
|  |  |  |  |  |  | MOL001525 | daucosterol |
|  |  |  |  |  |  | MOL002296 | daucosterol |
|  |  |  |  |  |  | MOL005868 | daucosterol |
|  |  |  |  |  |  | MOL007096 | daucosterol |
|  |  |  |  |  |  | MOL008172 | daucosterol |
|  |  |  |  |  |  | MOL008926 | daucosterol |
|  |  |  |  |  |  | MOL010584 | daucosterol |
|  |  |  |  |  |  | MOL012237 | daucosterol |
|  |  |  |  |  |  | MOL000448 | isobavachin |
|  |  |  |  |  |  | MOL001393 | myristic acid |
|  |  |  |  |  |  | MOL005639 | Neobavachalcone |
|  |  |  |  |  |  | MOL001950 | psoralen |
|  |  |  |  |  |  | MOL000860 | stearic acid |
|  |  |  |  |  |  | MOL000449 | stigmasterol |
|  |  |  |  |  |  | MOL002045 | stigmasterol |
|  |  |  |  |  |  | MOL008159 | TRIACONTANE |
|  | Baishao | Paeonia lactiflora Pall. | Paeoniaceae | Paeonia lactiflora | Root | MOL000359 | sitosterol |
|  |  |  |  |  |  | MOL000358 | beta-sitosterol |
|  |  |  |  |  |  | MOL000422 | kaempferol |
|  |  |  |  |  |  | MOL001919 | (3S,5R,8R,9R,10S,14S)-3,17-dihydroxy-4,4,8,10,14-pentamethyl-2,3,5,6,7,9-hexahydro-1H-cyclopenta[a]phenanthrene-15,16-dione |
|  |  |  |  |  |  | MOL000211 | Mairin |
|  |  |  |  |  |  | MOL001925 | paeoniflorin_qt |
|  |  |  |  |  |  | MOL001918 | paeoniflorgenone |
|  | Yinyanghuo | Epimedium brevicornu Maxim. | Berberidaceae | Epimedium brevicornu | Leaves | MOL000098 | quercetin |
|  |  |  |  |  |  | MOL000006 | luteolin |
|  |  |  |  |  |  | MOL000422 | kaempferol |
|  |  |  |  |  |  | MOL004382 | Yinyanghuo A |
|  |  |  |  |  |  | MOL003044 | Chryseriol |
|  |  |  |  |  |  | MOL001792 | DFV |
|  |  |  |  |  |  | MOL004386 | Yinyanghuo E |
|  |  |  |  |  |  | MOL003542 | 8-Isopentenyl-kaempferol |
|  |  |  |  |  |  | MOL004373 | Anhydroicaritin |
|  |  |  |  |  |  | MOL004384 | Yinyanghuo C |
|  |  |  |  |  |  | MOL004380 | C-Homoerythrinan, 1,6-didehydro-3,15,16-trimethoxy-, (3.beta.)- |
|  |  |  |  |  |  | MOL000359 | sitosterol |
|  |  |  |  |  |  | MOL001645 | Linoleyl acetate |
|  |  |  |  |  |  | MOL001510 | 24-epicampesterol |
|  |  |  |  |  |  | MOL001771 | poriferast-5-en-3beta-ol |
|  |  |  |  |  |  | MOL004391 | 8-(3-methylbut-2-enyl)-2-phenyl-chromone |
|  | Roucongrong | Cistanche deserticola Ma | Orobanchaceae | Cistanche deserticola | Stem | MOL000358 | [beta-sitosterol](https://www.tcmsp-e.com/molecule.php?qn=358) |
|  |  |  |  |  |  | MOL005320 | [arachidonate](https://www.tcmsp-e.com/molecule.php?qn=5320) |
|  |  |  |  |  |  | MOL005384 | [suchilactone](https://www.tcmsp-e.com/molecule.php?qn=5384) |
|  |  |  |  |  |  | MOL000098 | [quercetin](https://www.tcmsp-e.com/molecule.php?qn=98) |
|  |  |  |  |  |  | MOL008871 | [Marckine](https://www.tcmsp-e.com/molecule.php?qn=8871) |
|  | Dihuang | Rehmannia glutinosa (Gaertn.) Libosch. ex DC. | [Orobanchaceae](https://powo.science.kew.org/taxon/urn:lsid:ipni.org:names:30003446-2) | Rehmannia glutinosa | Root | MOL000359 | [sitosterol](https://www.tcmsp-e.com/molecule.php?qn=359) |
|  |  |  |  |  |  | MOL000449 | [Stigmasterol](https://www.tcmsp-e.com/molecule.php?qn=449) |
|  | Huangqi | [Astragalus mongholicus Bunge](https://mpns.science.kew.org/mpns-portal/plantDetail?plantId=2661222&query=Astragalus+membranaceus+%28Fisch.%29Bge.&filter=&fuzzy=false&nameType=all&dbs=wcs) | [Fabaceae](https://powo.science.kew.org/taxon/urn:lsid:ipni.org:names:30000147-2) | Astragalus mongholicus | Root | MOL000211 | Mairin |
|  |  |  |  |  |  | MOL000239 | Jaranol |
|  |  |  |  |  |  | MOL000296 | hederagenin |
|  |  |  |  |  |  | MOL000033 | (3S,8S,9S,10R,13R,14S,17R)-10,13-dimethyl-17-[(2R,5S)-5-propan-2-yloctan-2-yl]-2,3,4,7,8,9,11,12,14,15,16,17-dodecahydro-1H-cyclopenta[a]phenanthren-3-ol |
|  |  |  |  |  |  | MOL000354 | isorhamnetin |
|  |  |  |  |  |  | MOL000371 | 3,9-di-O-methylnissolin |
|  |  |  |  |  |  | MOL000380 | (6aR,11aR)-9,10-dimethoxy-6a,11a-dihydro-6H-benzofurano[3,2-c]chromen-3-ol |
|  |  |  |  |  |  | MOL000387 | Bifendate |
|  |  |  |  |  |  | MOL000392 | formononetin |
|  |  |  |  |  |  | MOL000398 | isoflavanone |
|  |  |  |  |  |  | MOL000417 | Calycosin |
|  |  |  |  |  |  | MOL000422 | kaempferol |
|  |  |  |  |  |  | MOL000442 | 1,7-Dihydroxy-3,9-dimethoxy pterocarpene |
|  | Tusizi | Cuscuta chinensis Lam. | Convolvulaceae | Cuscuta chinensis | Seed | MOL001558 | [sesamin](https://www.tcmsp-e.com/molecule.php?qn=1558) |
|  |  |  |  |  |  | MOL000184 | [NSC63551](https://www.tcmsp-e.com/molecule.php?qn=184) |
|  |  |  |  |  |  | MOL000354 | [isorhamnetin](https://www.tcmsp-e.com/molecule.php?qn=354) |
|  |  |  |  |  |  | MOL000358 | [beta-sitosterol](https://www.tcmsp-e.com/molecule.php?qn=358) |
|  |  |  |  |  |  | MOL000422 | [kaempferol](https://www.tcmsp-e.com/molecule.php?qn=422) |
|  |  |  |  |  |  | MOL005043 | [campest-5-en-3beta-ol](https://www.tcmsp-e.com/molecule.php?qn=5043) |
|  |  |  |  |  |  | MOL005440 | [Isofucosterol](https://www.tcmsp-e.com/molecule.php?qn=5440) |
|  |  |  |  |  |  | MOL005944 | [matrine](https://www.tcmsp-e.com/molecule.php?qn=5944) |
|  |  |  |  |  |  | MOL006649 | [sophranol](https://www.tcmsp-e.com/molecule.php?qn=6649) |
|  |  |  |  |  |  | MOL000953 | [CLR](https://www.tcmsp-e.com/molecule.php?qn=953) |
|  |  |  |  |  |  | MOL000098 | [quercetin](https://www.tcmsp-e.com/molecule.php?qn=98) |
|  | Danggui | Angelica sinensis (Oliv.) Diels | Apiaceae | Angelica sinensis | Root | MOL000358 | [beta-sitosterol](https://www.tcmsp-e.com/molecule.php?qn=358) |
|  |  |  |  |  |  | MOL000449 | [Stigmasterol](https://www.tcmsp-e.com/molecule.php?qn=449) |
|  | Danshen | Salvia miltiorrhiza Bunge | Lamiaceae | Salvia miltiorrhiza | Root | MOL001601 | 1,2,5,6-tetrahydrotanshinone |
|  |  |  |  |  |  | MOL001659 | Poriferasterol |
|  |  |  |  |  |  | MOL001771 | poriferast-5-en-3beta-ol |
|  |  |  |  |  |  | MOL002222 | sugiol |
|  |  |  |  |  |  | MOL002651 | Dehydrotanshinone II A |
|  |  |  |  |  |  | MOL000006 | luteolin |
|  |  |  |  |  |  | MOL007036 | 5,6-dihydroxy-7-isopropyl-1,1-dimethyl-2,3-dihydrophenanthren-4-one |
|  |  |  |  |  |  | MOL007041 | 2-isopropyl-8-methylphenanthrene-3,4-dione |
|  |  |  |  |  |  | MOL007045 | 3α-hydroxytanshinoneⅡa |
|  |  |  |  |  |  | MOL007048 | (E)-3-[2-(3,4-dihydroxyphenyl)-7-hydroxy-benzofuran-4-yl]acrylic acid |
|  |  |  |  |  |  | MOL007049 | 4-methylenemiltirone |
|  |  |  |  |  |  | MOL007050 | 2-(4-hydroxy-3-methoxyphenyl)-5-(3-hydroxypropyl)-7-methoxy-3-benzofurancarboxaldehyde |
|  |  |  |  |  |  | MOL007058 | formyltanshinone |
|  |  |  |  |  |  | MOL007059 | 3-beta-Hydroxymethyllenetanshiquinone |
|  |  |  |  |  |  | MOL007061 | Methylenetanshinquinone |
|  |  |  |  |  |  | MOL007068 | Przewaquinone B |
|  |  |  |  |  |  | MOL007069 | przewaquinone c |
|  |  |  |  |  |  | MOL007070 | (6S,7R)-6,7-dihydroxy-1,6-dimethyl-8,9-dihydro-7H-naphtho[8,7-g]benzofuran-10,11-dione |
|  |  |  |  |  |  | MOL007071 | przewaquinone f |
|  |  |  |  |  |  | MOL007077 | sclareol |
|  |  |  |  |  |  | MOL007079 | tanshinaldehyde |
|  |  |  |  |  |  | MOL007081 | Danshenol B |
|  |  |  |  |  |  | MOL007082 | Danshenol A |
|  |  |  |  |  |  | MOL007085 | Salvilenone |
|  |  |  |  |  |  | MOL007088 | cryptotanshinone |
|  |  |  |  |  |  | MOL007093 | dan-shexinkum d |
|  |  |  |  |  |  | MOL007094 | danshenspiroketallactone |
|  |  |  |  |  |  | MOL007098 | deoxyneocryptotanshinone |
|  |  |  |  |  |  | MOL007100 | dihydrotanshinlactone |
|  |  |  |  |  |  | MOL007101 | dihydrotanshinoneⅠ |
|  |  |  |  |  |  | MOL007108 | isocryptotanshi-none |
|  |  |  |  |  |  | MOL007111 | Isotanshinone II |
|  |  |  |  |  |  | MOL007115 | manool |
|  |  |  |  |  |  | MOL007118 | microstegiol |
|  |  |  |  |  |  | MOL007119 | miltionone Ⅰ |
|  |  |  |  |  |  | MOL007122 | Miltirone |
|  |  |  |  |  |  | MOL007124 | neocryptotanshinone ii |
|  |  |  |  |  |  | MOL007125 | neocryptotanshinone |
|  |  |  |  |  |  | MOL007127 | 1-methyl-8,9-dihydro-7H-naphtho[5,6-g]benzofuran-6,10,11-trione |
|  |  |  |  |  |  | MOL007130 | prolithospermic acid |
|  |  |  |  |  |  | MOL007140 | (Z)-3-[2-[(E)-2-(3,4-dihydroxyphenyl)vinyl]-3,4-dihydroxy-phenyl]acrylic acid |
|  |  |  |  |  |  | MOL007149 | NSC 122421 |
|  |  |  |  |  |  | MOL007150 | (6S)-6-hydroxy-1-methyl-6-methylol-8,9-dihydro-7H-naphtho[8,7-g]benzofuran-10,11-quinone |
|  |  |  |  |  |  | MOL007151 | Tanshindiol B |
|  |  |  |  |  |  | MOL007152 | Przewaquinone E |
|  |  |  |  |  |  | MOL007154 | tanshinone iia |
|  |  |  |  |  |  | MOL007155 | (6S)-6-(hydroxymethyl)-1,6-dimethyl-8,9-dihydro-7H-naphtho[8,7-g]benzofuran-10,11-dione |
|  |  |  |  |  |  | MOL007156 | tanshinone Ⅵ |
|  | Dazao | Ziziphus jujuba Mill. | Rhamnaceae | Ziziphus jujuba | Ripe fruit | MOL012921 | stepharine |
|  |  |  |  |  |  | MOL012940 | Spiradine A |
|  |  |  |  |  |  | MOL012946 | zizyphus saponin I_qt |
|  |  |  |  |  |  | MOL012961 | jujuboside A_qt |
|  |  |  |  |  |  | MOL012976 | coumestrol |
|  |  |  |  |  |  | MOL012980 | Daechuine S6 |
|  |  |  |  |  |  | MOL012981 | Daechuine S7 |
|  |  |  |  |  |  | MOL012986 | Jujubasaponin V_qt |
|  |  |  |  |  |  | MOL012989 | Jujuboside C_qt |
|  |  |  |  |  |  | MOL012992 | Mauritine D |
|  |  |  |  |  |  | MOL001454 | berberine |
|  |  |  |  |  |  | MOL001522 | (S)-Coclaurine |
|  |  |  |  |  |  | MOL000211 | Mairin |
|  |  |  |  |  |  | MOL000449 | Stigmasterol |
|  |  |  |  |  |  | MOL003410 | Ziziphin_qt |
|  |  |  |  |  |  | MOL000358 | beta-sitosterol |
|  |  |  |  |  |  | MOL004350 | Ruvoside_qt |
|  |  |  |  |  |  | MOL005360 | malkangunin |
|  |  |  |  |  |  | MOL000787 | Fumarine |
|  |  |  |  |  |  | MOL008034 | 21302-79-4 |
|  |  |  |  |  |  | MOL002773 | beta-carotene |
|  |  |  |  |  |  | MOL000098 | quercetin |
|  |  |  |  |  |  | MOL013357 | (3S,6R,8S,9S,10R,13R,14S,17R)-17-[(1R,4R)-4-ethyl-1,5-dimethylhexyl]-10,13-dimethyl-2,3,6,7,8,9,11,12,14,15,16,17-dodecahydro-1H-cyclopenta[a]phenanthrene-3,6-diol |
| Bushen Huatan Recipe | Tusizi | Cuscuta chinensis Lam. | Convolvulaceae | Cuscuta chinensis | Seed | MOL001558 | [sesamin](https://www.tcmsp-e.com/molecule.php?qn=1558) |
|  |  |  |  |  |  | MOL000184 | [NSC63551](https://www.tcmsp-e.com/molecule.php?qn=184) |
|  |  |  |  |  |  | MOL000354 | [isorhamnetin](https://www.tcmsp-e.com/molecule.php?qn=354) |
|  |  |  |  |  |  | MOL000358 | [beta-sitosterol](https://www.tcmsp-e.com/molecule.php?qn=358) |
|  |  |  |  |  |  | MOL000422 | [kaempferol](https://www.tcmsp-e.com/molecule.php?qn=422) |
|  |  |  |  |  |  | MOL005043 | [campest-5-en-3beta-ol](https://www.tcmsp-e.com/molecule.php?qn=5043) |
|  |  |  |  |  |  | MOL005440 | [Isofucosterol](https://www.tcmsp-e.com/molecule.php?qn=5440) |
|  |  |  |  |  |  | MOL005944 | [matrine](https://www.tcmsp-e.com/molecule.php?qn=5944) |
|  |  |  |  |  |  | MOL006649 | [sophranol](https://www.tcmsp-e.com/molecule.php?qn=6649) |
|  |  |  |  |  |  | MOL000953 | [CLR](https://www.tcmsp-e.com/molecule.php?qn=953) |
|  |  |  |  |  |  | MOL000098 | [quercetin](https://www.tcmsp-e.com/molecule.php?qn=98) |
|  | Yinyanghuo | Epimedium brevicornu Maxim. | Berberidaceae | Epimedium brevicornu | Leaves | MOL000098 | quercetin |
|  |  |  |  |  |  | MOL000006 | luteolin |
|  |  |  |  |  |  | MOL000422 | kaempferol |
|  |  |  |  |  |  | MOL004382 | Yinyanghuo A |
|  |  |  |  |  |  | MOL003044 | Chryseriol |
|  |  |  |  |  |  | MOL001792 | DFV |
|  |  |  |  |  |  | MOL004386 | Yinyanghuo E |
|  |  |  |  |  |  | MOL003542 | 8-Isopentenyl-kaempferol |
|  |  |  |  |  |  | MOL004373 | Anhydroicaritin |
|  |  |  |  |  |  | MOL004384 | Yinyanghuo C |
|  |  |  |  |  |  | MOL004380 | C-Homoerythrinan, 1,6-didehydro-3,15,16-trimethoxy-, (3.beta.)- |
|  |  |  |  |  |  | MOL000359 | sitosterol |
|  |  |  |  |  |  | MOL001645 | Linoleyl acetate |
|  |  |  |  |  |  | MOL001510 | 24-epicampesterol |
|  |  |  |  |  |  | MOL001771 | poriferast-5-en-3beta-ol |
|  |  |  |  |  |  | MOL004391 | 8-(3-methylbut-2-enyl)-2-phenyl-chromone |
|  | Buguzhi | Cullen corylifolium (L.) Medik. | Fabaceae | Cullen corylifolium | Dried ripe fruit | MOL003590 | angelicin |
|  |  |  |  |  |  | MOL005009 | Corylifolinin |
|  |  |  |  |  |  | MOL000093 | daucosterol |
|  |  |  |  |  |  | MOL001525 | daucosterol |
|  |  |  |  |  |  | MOL002296 | daucosterol |
|  |  |  |  |  |  | MOL005868 | daucosterol |
|  |  |  |  |  |  | MOL007096 | daucosterol |
|  |  |  |  |  |  | MOL008172 | daucosterol |
|  |  |  |  |  |  | MOL008926 | daucosterol |
|  |  |  |  |  |  | MOL010584 | daucosterol |
|  |  |  |  |  |  | MOL012237 | daucosterol |
|  |  |  |  |  |  | MOL000448 | isobavachin |
|  |  |  |  |  |  | MOL001393 | myristic acid |
|  |  |  |  |  |  | MOL005639 | Neobavachalcone |
|  |  |  |  |  |  | MOL001950 | psoralen |
|  |  |  |  |  |  | MOL000860 | stearic acid |
|  |  |  |  |  |  | MOL000449 | stigmasterol |
|  |  |  |  |  |  | MOL002045 | stigmasterol |
|  |  |  |  |  |  | MOL008159 | TRIACONTANE |
|  | Gualou | Trichosanthes kirilowii Maxim. | Cucurbitaceae | Trichosanthes kirilowii | Fruit | MOL001494 | Mandenol |
|  |  |  |  |  |  | MOL002881 | Diosmetin |
|  |  |  |  |  |  | MOL004355 | Spinasterol |
|  |  |  |  |  |  | MOL005530 | Hydroxygenkwanin |
|  |  |  |  |  |  | MOL006756 | Schottenol |
|  |  |  |  |  |  | MOL007165 | 10α-cucurbita-5,24-diene-3β-ol |
|  |  |  |  |  |  | MOL007179 | Linolenic acid ethyl ester |
|  | Hongqu | Monascus purpureus | Aspergillaceae | Monascus purpureus | Fermented product | MOL001691 | vitamin c |
|  | Shanzha | Crataegus monogyna Jacq. | Rosaceae | Crataegus monogyna | Ripe fruit | MOL000354 | isorhamnetin |
|  |  |  |  |  |  | MOL000359 | sitosterol |
|  |  |  |  |  |  | MOL000422 | kaempferol |
|  |  |  |  |  |  | MOL000449 | Stigmasterol |
|  |  |  |  |  |  | MOL000098 | quercetin |
| Bu-Shen-YiQi Decoction | Yinyanghuo | Epimedium brevicornu Maxim. | [Berberidaceae](https://powo.science.kew.org/taxon/urn:lsid:ipni.org:names:30000989-2) | Epimedium brevicornu | Leaves | MOL000098 | quercetin |
|  |  |  |  |  |  | MOL000006 | luteolin |
|  |  |  |  |  |  | MOL000422 | kaempferol |
|  |  |  |  |  |  | MOL004382 | Yinyanghuo A |
|  |  |  |  |  |  | MOL003044 | Chryseriol |
|  |  |  |  |  |  | MOL001792 | DFV |
|  |  |  |  |  |  | MOL004386 | Yinyanghuo E |
|  |  |  |  |  |  | MOL003542 | 8-Isopentenyl-kaempferol |
|  |  |  |  |  |  | MOL004373 | Anhydroicaritin |
|  |  |  |  |  |  | MOL004384 | Yinyanghuo C |
|  |  |  |  |  |  | MOL004380 | C-Homoerythrinan, 1,6-didehydro-3,15,16-trimethoxy-, (3.beta.)- |
|  |  |  |  |  |  | MOL000359 | sitosterol |
|  |  |  |  |  |  | MOL001645 | Linoleyl acetate |
|  |  |  |  |  |  | MOL001510 | 24-epicampesterol |
|  |  |  |  |  |  | MOL001771 | poriferast-5-en-3beta-ol |
|  |  |  |  |  |  | MOL004391 | 8-(3-methylbut-2-enyl)-2-phenyl-chromone |
|  | Huangqi | Astragalus mongholicus Bunge | Fabaceae | Astragalus mongholicus | Root | MOL000211 | Mairin |
|  |  |  |  |  |  | MOL000239 | Jaranol |
|  |  |  |  |  |  | MOL000296 | hederagenin |
|  |  |  |  |  |  | MOL000033 | (3S,8S,9S,10R,13R,14S,17R)-10,13-dimethyl-17-[(2R,5S)-5-propan-2-yloctan-2-yl]-2,3,4,7,8,9,11,12,14,15,16,17-dodecahydro-1H-cyclopenta[a]phenanthren-3-ol |
|  |  |  |  |  |  | MOL000354 | isorhamnetin |
|  |  |  |  |  |  | MOL000371 | 3,9-di-O-methylnissolin |
|  |  |  |  |  |  | MOL000380 | (6aR,11aR)-9,10-dimethoxy-6a,11a-dihydro-6H-benzofurano[3,2-c]chromen-3-ol |
|  |  |  |  |  |  | MOL000387 | Bifendate |
|  |  |  |  |  |  | MOL000392 | formononetin |
|  |  |  |  |  |  | MOL000398 | isoflavanone |
|  |  |  |  |  |  | MOL000417 | Calycosin |
|  |  |  |  |  |  | MOL000422 | kaempferol |
|  |  |  |  |  |  | MOL000442 | 1,7-Dihydroxy-3,9-dimethoxy pterocarpene |
|  | Dihuang | Rehmannia glutinosa (Gaertn.) Libosch. ex DC. | [Orobanchaceae](https://powo.science.kew.org/taxon/urn:lsid:ipni.org:names:30003446-2) | Rehmannia glutinosa | Root | MOL000359 | [sitosterol](https://www.tcmsp-e.com/molecule.php?qn=359) |
|  |  |  |  |  |  | MOL000449 | [Stigmasterol](https://www.tcmsp-e.com/molecule.php?qn=449) |
|  | Huangqin | Scutellaria baicalensis Georgi | Lamiaceae | Scutellaria baicalensis | Root | MOL001458 | coptisine |
|  |  |  |  |  |  | MOL000173 | wogonin |
|  |  |  |  |  |  | MOL000552 | 5,2'-Dihydroxy-6,7,8-trimethoxyflavone |
|  |  |  |  |  |  | MOL002714 | baicalein |
|  |  |  |  |  |  | MOL002909 | 5,7,2,5-tetrahydroxy-8,6-dimethoxyflavone |
|  |  |  |  |  |  | MOL001689 | acacetin |
|  |  |  |  |  |  | MOL002933 | 5,7,4'-Trihydroxy-8-methoxyflavone |
|  |  |  |  |  |  | MOL012245 | 5,7,4'-trihydroxy-6-methoxyflavanone |
|  |  |  |  |  |  | MOL000359 | sitosterol |
|  |  |  |  |  |  | MOL000358 | beta-sitosterol |
|  |  |  |  |  |  | MOL002908 | 5,8,2'-Trihydroxy-7-methoxyflavone |
|  |  |  |  |  |  | MOL002925 | 5,7,2',6'-Tetrahydroxyflavone |
|  |  |  |  |  |  | MOL012266 | rivularin |
|  |  |  |  |  |  | MOL002926 | dihydrooroxylin A |
|  |  |  |  |  |  | MOL010415 | 11,13-Eicosadienoic acid, methyl ester |
|  |  |  |  |  |  | MOL000525 | Norwogonin |
|  |  |  |  |  |  | MOL002913 | Dihydrobaicalin_qt |
|  |  |  |  |  |  | MOL002910 | Carthamidin |
|  |  |  |  |  |  | MOL002914 | Eriodyctiol (flavanone) |
|  |  |  |  |  |  | MOL002928 | oroxylin a |
|  |  |  |  |  |  | MOL002897 | epiberberine |
|  |  |  |  |  |  | MOL000449 | Stigmasterol |
|  |  |  |  |  |  | MOL008206 | Moslosooflavone |
|  |  |  |  |  |  | MOL002917 | 5,2',6'-Trihydroxy-7,8-dimethoxyflavone |
|  |  |  |  |  |  | MOL002915 | Salvigenin |
|  |  |  |  |  |  | MOL000228 | (2R)-7-hydroxy-5-methoxy-2-phenylchroman-4-one |
|  |  |  |  |  |  | MOL002937 | DIHYDROOROXYLIN |
|  |  |  |  |  |  | MOL002911 | 2,6,2',4'-tetrahydroxy-6'-methoxychaleone |
|  |  |  |  |  |  | MOL002927 | Skullcapflavone II |
|  |  |  |  |  |  | MOL012246 | 5,7,4'-trihydroxy-8-methoxyflavanone |
|  |  |  |  |  |  | MOL002932 | Panicolin |
|  |  |  |  |  |  | MOL002934 | NEOBAICALEIN |
|  | Chishao | Paeonia lactiflora Pall. | Paeoniaceae | Paeonia lactiflora | **Root** | MOL001918 | paeoniflorgenone |
|  |  |  |  |  |  | MOL001925 | paeoniflorin_qt |
|  |  |  |  |  |  | MOL002714 | baicalein |
|  |  |  |  |  |  | MOL000358 | beta-sitosterol |
|  |  |  |  |  |  | MOL000359 | sitosterol |
|  |  |  |  |  |  | MOL004355 | Spinasterol |
|  |  |  |  |  |  | MOL000449 | Stigmasterol |
|  |  |  |  |  |  | MOL006992 | (2R,3R)-4-methoxyl-distylin |
|  |  |  |  |  |  | MOL006994 | 1-o-beta-d-glucopyranosyl-8-o-benzoylpaeonisuffrone_qt |
|  |  |  |  |  |  | MOL006996 | 1-o-beta-d-glucopyranosylpaeonisuffrone_qt |
|  |  |  |  |  |  | MOL006999 | stigmast-7-en-3-ol |
|  |  |  |  |  |  | MOL007005 | Albiflorin_qt |
|  |  |  |  |  |  | MOL007008 | 4-ethyl-paeoniflorin_qt |
|  |  |  |  |  |  | MOL007012 | 4-o-methyl-paeoniflorin_qt |
|  |  |  |  |  |  | MOL007016 | Paeoniflorigenone |
|  |  |  |  |  |  | MOL007022 | evofolinB |
|  |  |  |  |  |  | MOL002883 | Ethyl oleate (NF) |
|  |  |  |  |  |  | MOL005043 | campest-5-en-3beta-ol |
| Yiwei Decoction | Dangshen | Codonopsis pilosula (Franch.) Nannf. | [Campanulaceae](https://powo.science.kew.org/taxon/urn:lsid:ipni.org:names:30000171-2) | Codonopsis pilosula | Root | MOL001006 | [poriferasta-7,22E-dien-3beta-ol](https://www.tcmsp-e.com/molecule.php?qn=1006) |
|  |  |  |  |  |  | MOL002140 | [Perlolyrine](https://www.tcmsp-e.com/molecule.php?qn=2140) |
|  |  |  |  |  |  | MOL003036 | [ZINC03978781](https://www.tcmsp-e.com/molecule.php?qn=3036) |
|  |  |  |  |  |  | MOL000449 | [Stigmasterol](https://www.tcmsp-e.com/molecule.php?qn=449) |
|  |  |  |  |  |  | MOL003896 | [7-Methoxy-2-methyl isoflavone](https://www.tcmsp-e.com/molecule.php?qn=3896) |
|  |  |  |  |  |  | MOL004355 | [Spinasterol](https://www.tcmsp-e.com/molecule.php?qn=4355) |
|  |  |  |  |  |  | MOL004492 | [Chrysanthemaxanthin](https://www.tcmsp-e.com/molecule.php?qn=4492) |
|  |  |  |  |  |  | MOL005321 | [Frutinone A](https://www.tcmsp-e.com/molecule.php?qn=5321) |
|  |  |  |  |  |  | MOL000006 | [luteolin](https://www.tcmsp-e.com/molecule.php?qn=6) |
|  |  |  |  |  |  | MOL006774 | [stigmast-7-enol](https://www.tcmsp-e.com/molecule.php?qn=6774) |
|  |  |  |  |  |  | MOL007059 | [3-beta-Hydroxymethyllenetanshiquinone](https://www.tcmsp-e.com/molecule.php?qn=7059) |
|  |  |  |  |  |  | MOL007514 | [methyl icosa-11,14-dienoate](https://www.tcmsp-e.com/molecule.php?qn=7514) |
|  |  |  |  |  |  | MOL008391 | [5alpha-Stigmastan-3,6-dione](https://www.tcmsp-e.com/molecule.php?qn=8391) |
|  |  |  |  |  |  | MOL008397 | [Daturilin](https://www.tcmsp-e.com/molecule.php?qn=8397) |
|  |  |  |  |  |  | MOL008400 | [glycitein](https://www.tcmsp-e.com/molecule.php?qn=8400) |
|  |  |  |  |  |  | MOL008407 | [(8S,9S,10R,13R,14S,17R)-17-[(E,2R,5S)-5-ethyl-6-methylhept-3-en-2-yl]-10,13-dimethyl-1,2,4,7,8,9,11,12,14,15,16,17-dodecahydrocyclopenta[a]phenanthren-3-one](https://www.tcmsp-e.com/molecule.php?qn=8407) |
|  |  |  |  |  |  | MOL008411 | [11-Hydroxyrankinidine](https://www.tcmsp-e.com/molecule.php?qn=8411) |
|  | Baizhu | Atractylodes macrocephala Koidz. | Asteraceae | Atractylodes macrocephala | Rhizome | MOL000033 | (3S,8S,9S,10R,13R,14S,17R)-10,13-dimethyl-17-[(2R,5S)-5-propan-2-yloctan-2-yl]-2,3,4,7,8,9,11,12,14,15,16,17-dodecahydro-1H-cyclopenta[a]phenanthren-3-ol |
|  |  |  |  |  |  | MOL000021 | 14-acetyl-12-senecioyl-2E,8E,10E-atractylentriol |
|  |  |  |  |  |  | MOL000020 | 12-senecioyl-2E,8E,10E-atractylentriol |
|  |  |  |  |  |  | MOL000022 | 14-acetyl-12-senecioyl-2E,8Z,10E-atractylentriol |
|  |  |  |  |  |  | MOL000072 | 8β-ethoxy atractylenolide Ⅲ |
|  | fuling | Ophrys apifera Huds. | [Orchidaceae](https://powo.science.kew.org/taxon/urn:lsid:ipni.org:names:30000046-2) | Ophrys apifera | Sclerotium | MOL000291 | Poricoic acid B |
|  |  |  |  |  |  | MOL000290 | Poricoic acid A |
|  |  |  |  |  |  | MOL000273 | (2R)-2-[(3S,5R,10S,13R,14R,16R,17R)-3,16-dihydroxy-4,4,10,13,14-pentamethyl-2,3,5,6,12,15,16,17-octahydro-1H-cyclopenta[a]phenanthren-17-yl]-6-methylhept-5-enoic acid |
|  |  |  |  |  |  | MOL000280 | (2R)-2-[(3S,5R,10S,13R,14R,16R,17R)-3,16-dihydroxy-4,4,10,13,14-pentamethyl-2,3,5,6,12,15,16,17-octahydro-1H-cyclopenta[a]phenanthren-17-yl]-5-isopropyl-hex-5-enoic acid |
|  |  |  |  |  |  | MOL000289 | pachymic acid |
|  |  |  |  |  |  | MOL000276 | 7,9(11)-dehydropachymic acid |
|  |  |  |  |  |  | MOL000296 | hederagenin |
|  |  |  |  |  |  | MOL000279 | Cerevisterol |
|  |  |  |  |  |  | MOL000292 | poricoic acid C |
|  |  |  |  |  |  | MOL000285 | (2R)-2-[(5R,10S,13R,14R,16R,17R)-16-hydroxy-3-keto-4,4,10,13,14-pentamethyl-1,2,5,6,12,15,16,17-octahydrocyclopenta[a]phenanthren-17-yl]-5-isopropyl-hex-5-enoic acid |
|  |  |  |  |  |  | MOL000287 | 3beta-Hydroxy-24-methylene-8-lanostene-21-oic acid |
|  |  |  |  |  |  | MOL000275 | trametenolic acid |
|  |  |  |  |  |  | MOL000282 | ergosta-7,22E-dien-3beta-ol |
|  |  |  |  |  |  | MOL000300 | dehydroeburicoic acid |
|  | Gancao | Glycyrrhiza glabra L. | Fabaceae | Glycyrrhiza glabra | Rhizome and root | MOL001484 | Inermine |
|  |  |  |  |  |  | MOL001792 | DFV |
|  |  |  |  |  |  | MOL000211 | Mairin |
|  |  |  |  |  |  | MOL002311 | Glycyrol |
|  |  |  |  |  |  | MOL000239 | Jaranol |
|  |  |  |  |  |  | MOL002565 | Medicarpin |
|  |  |  |  |  |  | MOL000354 | isorhamnetin |
|  |  |  |  |  |  | MOL000359 | sitosterol |
|  |  |  |  |  |  | MOL003656 | Lupiwighteone |
|  |  |  |  |  |  | MOL003896 | 7-Methoxy-2-methyl isoflavone |
|  |  |  |  |  |  | MOL000392 | formononetin |
|  |  |  |  |  |  | MOL000417 | Calycosin |
|  |  |  |  |  |  | MOL000422 | kaempferol |
|  |  |  |  |  |  | MOL004328 | naringenin |
|  |  |  |  |  |  | MOL004805 | (2S)-2-[4-hydroxy-3-(3-methylbut-2-enyl)phenyl]-8,8-dimethyl-2,3-dihydropyrano[2,3-f]chromen-4-one |
|  |  |  |  |  |  | MOL004806 | euchrenone |
|  |  |  |  |  |  | MOL004808 | glyasperin B |
|  |  |  |  |  |  | MOL004810 | glyasperin F |
|  |  |  |  |  |  | MOL004814 | Isotrifoliol |
|  |  |  |  |  |  | MOL004815 | (E)-1-(2,4-dihydroxyphenyl)-3-(2,2-dimethylchromen-6-yl)prop-2-en-1-one |
|  |  |  |  |  |  | MOL004824 | (2S)-6-(2,4-dihydroxyphenyl)-2-(2-hydroxypropan-2-yl)-4-methoxy-2,3-dihydrofuro[3,2-g]chromen-7-one |
|  |  |  |  |  |  | MOL004827 | Semilicoisoflavone B |
|  |  |  |  |  |  | MOL004828 | Glepidotin A |
|  |  |  |  |  |  | MOL004829 | Glepidotin B |
|  |  |  |  |  |  | MOL004835 | Glypallichalcone |
|  |  |  |  |  |  | MOL004838 | 8-(6-hydroxy-2-benzofuranyl)-2,2-dimethyl-5-chromenol |
|  |  |  |  |  |  | MOL004841 | Licochalcone B |
|  |  |  |  |  |  | MOL004848 | licochalcone G |
|  |  |  |  |  |  | MOL004855 | Licoricone |
|  |  |  |  |  |  | MOL004856 | Gancaonin A |
|  |  |  |  |  |  | MOL004857 | Gancaonin B |
|  |  |  |  |  |  | MOL004863 | 3-(3,4-dihydroxyphenyl)-5,7-dihydroxy-8-(3-methylbut-2-enyl)chromone |
|  |  |  |  |  |  | MOL004864 | 5,7-dihydroxy-3-(4-methoxyphenyl)-8-(3-methylbut-2-enyl)chromone |
|  |  |  |  |  |  | MOL004866 | 2-(3,4-dihydroxyphenyl)-5,7-dihydroxy-6-(3-methylbut-2-enyl)chromone |
|  |  |  |  |  |  | MOL004882 | Licocoumarone |
|  |  |  |  |  |  | MOL004883 | Licoisoflavone |
|  |  |  |  |  |  | MOL004884 | Licoisoflavone B |
|  |  |  |  |  |  | MOL004885 | licoisoflavanone |
|  |  |  |  |  |  | MOL004891 | shinpterocarpin |
|  |  |  |  |  |  | MOL004898 | (E)-3-[3,4-dihydroxy-5-(3-methylbut-2-enyl)phenyl]-1-(2,4-dihydroxyphenyl)prop-2-en-1-one |
|  |  |  |  |  |  | MOL004907 | Glyzaglabrin |
|  |  |  |  |  |  | MOL004910 | Glabranin |
|  |  |  |  |  |  | MOL004912 | Glabrone |
|  |  |  |  |  |  | MOL004913 | 1,3-dihydroxy-9-methoxy-6-benzofurano[3,2-c]chromenone |
|  |  |  |  |  |  | MOL004914 | 1,3-dihydroxy-8,9-dimethoxy-6-benzofurano[3,2-c]chromenone |
|  |  |  |  |  |  | MOL004915 | Eurycarpin A |
|  |  |  |  |  |  | MOL004935 | Sigmoidin-B |
|  |  |  |  |  |  | MOL004941 | (2R)-7-hydroxy-2-(4-hydroxyphenyl)chroman-4-one |
|  |  |  |  |  |  | MOL004945 | (2S)-7-hydroxy-2-(4-hydroxyphenyl)-8-(3-methylbut-2-enyl)chroman-4-one |
|  |  |  |  |  |  | MOL004948 | Isoglycyrol |
|  |  |  |  |  |  | MOL004949 | Isolicoflavonol |
|  |  |  |  |  |  | MOL004957 | HMO |
|  |  |  |  |  |  | MOL004959 | 1-Methoxyphaseollidin |
|  |  |  |  |  |  | MOL004961 | Quercetin der. |
|  |  |  |  |  |  | MOL000497 | licochalcone a |
|  |  |  |  |  |  | MOL004985 | icos-5-enoic acid |
|  |  |  |  |  |  | MOL004988 | Kanzonol F |
|  |  |  |  |  |  | MOL004989 | 6-prenylated eriodictyol |
|  |  |  |  |  |  | MOL004991 | 7-Acetoxy-2-methylisoflavone |
|  |  |  |  |  |  | MOL004993 | 8-prenylated eriodictyol |
|  |  |  |  |  |  | MOL004996 | gadelaidic acid |
|  |  |  |  |  |  | MOL005000 | Gancaonin G |
|  |  |  |  |  |  | MOL005001 | Gancaonin H |
|  |  |  |  |  |  | MOL005003 | Licoagrocarpin |
|  |  |  |  |  |  | MOL005007 | Glyasperins M |
|  |  |  |  |  |  | MOL005008 | Glycyrrhiza flavonol A |
|  |  |  |  |  |  | MOL005012 | Licoagroisoflavone |
|  |  |  |  |  |  | MOL005013 | 18α-hydroxyglycyrrhetic acid |
|  |  |  |  |  |  | MOL005016 | Odoratin |
|  |  |  |  |  |  | MOL005017 | Phaseol |
|  |  |  |  |  |  | MOL005018 | Xambioona |
|  |  |  |  |  |  | MOL000098 | quercetin |
|  | Chenpi | Citrus reticulata Blanco | [Rutaceae](https://powo.science.kew.org/taxon/urn:lsid:ipni.org:names:30001492-2) | Citrus reticulata | Dried pericarp | MOL000359 | [sitosterol](https://www.tcmsp-e.com/molecule.php?qn=359) |
|  |  |  |  |  |  | MOL004328 | [naringenin](https://www.tcmsp-e.com/molecule.php?qn=4328) |
|  |  |  |  |  |  | MOL005100 | [5,7-dihydroxy-2-(3-hydroxy-4-methoxyphenyl)chroman-4-one](https://www.tcmsp-e.com/molecule.php?qn=5100) |
|  |  |  |  |  |  | MOL005815 | [Citromitin](https://www.tcmsp-e.com/molecule.php?qn=5815) |
|  |  |  |  |  |  | MOL005828 | [nobiletin](https://www.tcmsp-e.com/molecule.php?qn=5828) |
|  | Muxiang | Dolomiaea costus (Falc.) Kasana & A.K.Pandey | Asteraceae | Dolomiaea costus | Root | MOL010813 | Benzo[a]carbazole |
|  |  |  |  |  |  | MOL010839 | lappadilactone |
|  |  |  |  |  |  | MOL000211 | Mairin |
|  |  |  |  |  |  | MOL000359 | sitosterol |
|  |  |  |  |  |  | MOL000449 | Stigmasterol |
|  | Sharen | Wurfbainia villosa (Lour.) Škorničk. & A.D.Poulsen | Zingiberaceae | Wurfbainia villosa | Ripe fruit | MOL007535 | (5S,8S,9S,10R,13R,14S,17R)-17-[(1R,4R)-4-ethyl-1,5-dimethylhexyl]-10,13-dimethyl-2,4,5,7,8,9,11,12,14,15,16,17-dodecahydro-1H-cyclopenta[a]phenanthrene-3,6-dione |
|  |  |  |  |  |  | MOL000358 | beta-sitosterol |
|  |  |  |  |  |  | MOL001973 | Sitosteryl acetate |
|  |  |  |  |  |  | MOL007536 | Stigmasta-5,22-dien-3-beta-yl acetate |
|  |  |  |  |  |  | MOL000449 | Stigmasterol |
|  |  |  |  |  |  | MOL001771 | poriferast-5-en-3beta-ol |
|  |  |  |  |  |  | MOL001755 | 24-Ethylcholest-4-en-3-one |
|  |  |  |  |  |  | MOL007514 | methyl icosa-11,14-dienoate |
|  |  |  |  |  |  | MOL003975 | icosa-11,14,17-trienoic acid methyl ester |
|  | Banxia | Pinellia ternata (Thunb.) Makino | Araceae | Pinellia ternata | Tuber | MOL001755 | [24-Ethylcholest-4-en-3-one](https://www.tcmsp-e.com/molecule.php?qn=1755) |
|  |  |  |  |  |  | MOL002670 | [Cavidine](https://www.tcmsp-e.com/molecule.php?qn=2670) |
|  |  |  |  |  |  | MOL002714 | [baicalein](https://www.tcmsp-e.com/molecule.php?qn=2714) |
|  |  |  |  |  |  | MOL000358 | [beta-sitosterol](https://www.tcmsp-e.com/molecule.php?qn=358) |
|  |  |  |  |  |  | MOL000449 | [Stigmasterol](https://www.tcmsp-e.com/molecule.php?qn=449) |
|  |  |  |  |  |  | MOL005030 | [gondoic acid](https://www.tcmsp-e.com/molecule.php?qn=5030) |
|  |  |  |  |  |  | MOL000519 | [coniferin](https://www.tcmsp-e.com/molecule.php?qn=519) |
|  |  |  |  |  |  | MOL006936 | [10,13-eicosadienoic](https://www.tcmsp-e.com/molecule.php?qn=6936) |
|  |  |  |  |  |  | MOL003578 | [Cycloartenol](https://www.tcmsp-e.com/molecule.php?qn=3578) |
| Liuwei DihuangPill | Dihuang | Rehmannia glutinosa (Gaertn.) Libosch. ex DC. | [Orobanchaceae](https://powo.science.kew.org/taxon/urn:lsid:ipni.org:names:30003446-2) | Rehmannia glutinosa | Root | MOL000359 | [sitosterol](https://www.tcmsp-e.com/molecule.php?qn=359) |
|  |  |  |  |  |  | MOL000449 | [Stigmasterol](https://www.tcmsp-e.com/molecule.php?qn=449) |
|  | Shanzhuyu | Cornus officinalis Siebold & Zucc. | Cornaceae | Cornus officinalis | Ripe fruit | MOL002883 | Ethyl oleate (NF) |
|  |  |  |  |  |  | MOL008457 | Tetrahydroalstonine |
|  |  |  |  |  |  | MOL005481 | 2,6,10,14,18-pentamethylicosa-2,6,10,14,18-pentaene |
|  |  |  |  |  |  | MOL005530 | Hydroxygenkwanin |
|  |  |  |  |  |  | MOL000359 | sitosterol |
|  |  |  |  |  |  | MOL001771 | poriferast-5-en-3beta-ol |
|  |  |  |  |  |  | MOL000358 | beta-sitosterol |
|  |  |  |  |  |  | MOL001494 | Mandenol |
|  |  |  |  |  |  | MOL000449 | Stigmasterol |
|  |  |  |  |  |  | MOL005557 | lanosta-8,24-dien-3-ol,3-acetate |
|  |  |  |  |  |  | MOL001495 | Ethyl linolenate |
|  |  |  |  |  |  | MOL005486 | 3,4-Dehydrolycopen-16-al |
|  |  |  |  |  |  | MOL005360 | malkangunin |
|  |  |  |  |  |  | MOL005531 | Telocinobufagin |
|  | Shanyao | Dioscorea oppositifolia L. | Dioscoreaceae | Dioscorea oppositifolia | Rhizome | MOL001559 | [piperlonguminine](https://www.tcmsp-e.com/molecule.php?qn=1559) |
|  |  |  |  |  |  | MOL001736 | [(-)-taxifolin](https://www.tcmsp-e.com/molecule.php?qn=1736) |
|  |  |  |  |  |  | MOL000310 | [Denudatin B](https://www.tcmsp-e.com/molecule.php?qn=310) |
|  |  |  |  |  |  | MOL000322 | [Kadsurenone](https://www.tcmsp-e.com/molecule.php?qn=322) |
|  |  |  |  |  |  | MOL005429 | [hancinol](https://www.tcmsp-e.com/molecule.php?qn=5429) |
|  |  |  |  |  |  | MOL005430 | [hancinone C](https://www.tcmsp-e.com/molecule.php?qn=5430) |
|  |  |  |  |  |  | MOL005435 | [24-Methylcholest-5-enyl-3belta-O-glucopyranoside_qt](https://www.tcmsp-e.com/molecule.php?qn=5435) |
|  |  |  |  |  |  | MOL005438 | [campesterol](https://www.tcmsp-e.com/molecule.php?qn=5438) |
|  |  |  |  |  |  | MOL005440 | [Isofucosterol](https://www.tcmsp-e.com/molecule.php?qn=5440) |
|  |  |  |  |  |  | MOL000449 | [Stigmasterol](https://www.tcmsp-e.com/molecule.php?qn=449) |
|  |  |  |  |  |  | MOL005458 | [Dioscoreside C_qt](https://www.tcmsp-e.com/molecule.php?qn=5458) |
|  |  |  |  |  |  | MOL000546 | [diosgenin](https://www.tcmsp-e.com/molecule.php?qn=546) |
|  |  |  |  |  |  | MOL005461 | [Doradexanthin](https://www.tcmsp-e.com/molecule.php?qn=5461) |
|  |  |  |  |  |  | MOL005463 | [Methylcimicifugoside_qt](https://www.tcmsp-e.com/molecule.php?qn=5463) |
|  |  |  |  |  |  | MOL005465 | [AIDS180907](https://www.tcmsp-e.com/molecule.php?qn=5465) |
|  |  |  |  |  |  | MOL000953 | [CLR](https://www.tcmsp-e.com/molecule.php?qn=953) |
|  | Zexie | Alisma plantago-aquatica subsp. orientale (Sam.) Sam. | Alismataceae | Alisma plantago-aquatica | Tuber | MOL000359 | sitosterol |
|  |  |  |  |  |  | MOL000830 | Alisol B |
|  |  |  |  |  |  | MOL000831 | Alisol B monoacetate |
|  |  |  |  |  |  | MOL000832 | alisol,b,23-acetate |
|  |  |  |  |  |  | MOL000849 | 16β-methoxyalisol B monoacetate |
|  |  |  |  |  |  | MOL000853 | alisol B |
|  |  |  |  |  |  | MOL000854 | alisol C |
|  |  |  |  |  |  | MOL000856 | alisol C monoacetate |
|  |  |  |  |  |  | MOL002464 | 1-Monolinolein |
|  |  |  |  |  |  | MOL000862 | [(1S,3R)-1-[(2R)-3,3-dimethyloxiran-2-yl]-3-[(5R,8S,9S,10S,11S,14R)-11-hydroxy-4,4,8,10,14-pentamethyl-3-oxo-1,2,5,6,7,9,11,12,15,16-decahydrocyclopenta[a]phenanthren-17-yl]butyl] acetate |
|  | fuling | Ophrys apifera Huds. | [Orchidaceae](https://powo.science.kew.org/taxon/urn:lsid:ipni.org:names:30000046-2) | Ophrys apifera | Sclerotium | MOL000291 | Poricoic acid B |
|  |  |  |  |  |  | MOL000290 | Poricoic acid A |
|  |  |  |  |  |  | MOL000273 | (2R)-2-[(3S,5R,10S,13R,14R,16R,17R)-3,16-dihydroxy-4,4,10,13,14-pentamethyl-2,3,5,6,12,15,16,17-octahydro-1H-cyclopenta[a]phenanthren-17-yl]-6-methylhept-5-enoic acid |
|  |  |  |  |  |  | MOL000280 | (2R)-2-[(3S,5R,10S,13R,14R,16R,17R)-3,16-dihydroxy-4,4,10,13,14-pentamethyl-2,3,5,6,12,15,16,17-octahydro-1H-cyclopenta[a]phenanthren-17-yl]-5-isopropyl-hex-5-enoic acid |
|  |  |  |  |  |  | MOL000289 | pachymic acid |
|  |  |  |  |  |  | MOL000276 | 7,9(11)-dehydropachymic acid |
|  |  |  |  |  |  | MOL000296 | hederagenin |
|  |  |  |  |  |  | MOL000279 | Cerevisterol |
|  |  |  |  |  |  | MOL000292 | poricoic acid C |
|  |  |  |  |  |  | MOL000285 | (2R)-2-[(5R,10S,13R,14R,16R,17R)-16-hydroxy-3-keto-4,4,10,13,14-pentamethyl-1,2,5,6,12,15,16,17-octahydrocyclopenta[a]phenanthren-17-yl]-5-isopropyl-hex-5-enoic acid |
|  |  |  |  |  |  | MOL000287 | 3beta-Hydroxy-24-methylene-8-lanostene-21-oic acid |
|  |  |  |  |  |  | MOL000275 | trametenolic acid |
|  |  |  |  |  |  | MOL000282 | ergosta-7,22E-dien-3beta-ol |
|  |  |  |  |  |  | MOL000300 | dehydroeburicoic acid |
|  | Mudanpi | Paeonia suffruticosa Andrews | Paeoniaceae | Paeonia suffruticosa | Root bark | MOL000359 | [sitosterol](https://www.tcmsp-e.com/molecule.php?qn=359) |
|  |  |  |  |  |  | MOL007384 | [paeonidanin_qt](https://www.tcmsp-e.com/molecule.php?qn=7384) |
|  |  |  |  |  |  | MOL007382 | [mudanpioside-h_qt 2](https://www.tcmsp-e.com/molecule.php?qn=7382) |
|  |  |  |  |  |  | MOL001925 | [paeoniflorin_qt](https://www.tcmsp-e.com/molecule.php?qn=1925) |
|  |  |  |  |  |  | MOL000211 | [Mairin](https://www.tcmsp-e.com/molecule.php?qn=211) |
|  |  |  |  |  |  | MOL007369 | [4-O-methylpaeoniflorin_qt](https://www.tcmsp-e.com/molecule.php?qn=7369) |
|  |  |  |  |  |  | MOL000098 | [quercetin](https://www.tcmsp-e.com/molecule.php?qn=98) |
|  |  |  |  |  |  | MOL000422 | [kaempferol](https://www.tcmsp-e.com/molecule.php?qn=422) |
